# Relationships between Neonatal Nutrition and Growth to 36 Weeks’ Corrected Age in ELBW Babies–Secondary Cohort Analysis from the Provide Trial

**DOI:** 10.3390/nu12030760

**Published:** 2020-03-13

**Authors:** Barbara E. Cormack, Yannan Jiang, Jane E. Harding, Caroline A. Crowther, Frank H. Bloomfield

**Affiliations:** 1 Liggins Institute, University of Auckland, 1142 Auckland, New Zealand; bcormack@me.com (B.E.C.); y.jiang@auckland.ac.nz (Y.J.); j.harding@auckland.ac.nz (J.E.H.); c.crowther@auckland.ac.nz (C.A.C.); 2 Newborn Services, Starship Child Health, Auckland City Hospital, 1142 Auckland, New Zealand

**Keywords:** growth, nutrition, parenteral nutrition, protein, amino acid, extremely low birthweight, preterm, premature, newborn, gestational age, infant

## Abstract

A key modifiable factor for improving neurodevelopment in extremely low birthweight (ELBW) babies may be improving growth, especially head growth, by optimising nutrition in the early neonatal period. We aimed to investigate relationships between nutrient intakes in the 4 weeks after birth, and growth from birth to 36 weeks’ corrected age (CA) in ELBW babies. We undertook a prospective cohort study of 434 participants enrolled in a randomised controlled trial (ProVIDe) in eight New Zealand and Australian neonatal intensive care units. Macronutrient intakes from birth to 4 weeks and weight, length and head circumference measurements from birth to 36 weeks’ CA were collected. From birth to 36 weeks’ CA, the median (IQR) z-score changes were: weight −0.48 (−1.09, 0.05); length −1.16 (−1.86, −0.43), and head circumference −0.82 (−1.51, −0.19). Changes in z-score to 4 weeks and 36 weeks’ CA were correlated with protein intake. Each 1 g·Kg^−1^·d^−1^ total protein intake in week 2 was associated with 0.26 z-score increase in head circumference at 36 weeks’ CA. Both nutritional intake and change in z-scores to 36 weeks’ CA differed widely amongst sites. Correlations between nutrition and growth, and differences in these amongst sites, indicate there may be potential to improve growth with enhanced nutrition practices.

## 1. Introduction

Extremely preterm birth has long-term effects on growth and neurodevelopment. Low nutritional stores at birth and rapid postnatal growth potential mean extremely low birthweight (ELBW; birthweight <1000 g) babies have very high nutrient requirements [1,2,3,4]. Despite best efforts to meet these high requirements, faltering growth remains common in ELBW babies [5] and occurs during a critical period of brain growth and neurodevelopment [6,7]. Suboptimal neurodevelopment affects 20–45% of preterm babies and up to 50% of ELBW babies [8]. The current recommendation for growth of ELBW babies is that it should match fetal growth rates [9], although evidence that this improves neurodevelopment is lacking [10,11]. While recent improvements to neonatal care and nutrition have made it possible for some neonatal units to match fetal growth rates [12,13,14,15], worldwide, many ELBW babies continue to have postnatal malnutrition, faltering growth and less than optimal neurodevelopment compared with their term-born peers [8].

There is a strong correlation between head circumference and brain volume [16], and head circumference growth and neurodevelopmental outcome, in preterm babies [17,18,19]. Enhanced nutrition and higher protein intakes in the first month after birth have been associated with improved head circumference and head circumference growth at discharge [13,20,21,22,23], and cognitive outcome in adolescence [16,24,25,26]. Therefore, a key modifiable factor for improving neurodevelopment in ELBW babies may be improved growth, especially head growth, through optimised nutritional intake in the early neonatal period. To date, meta-analyses of randomised controlled trials (RCTs) of higher versus lower protein intakes are inconclusive for growth and neurodevelopmental outcomes [10,27]; therefore, it remains uncertain how intakes of protein and other nutritional components influence growth and neurodevelopment for ELBW babies and, in particular, whether higher early protein intakes improve head circumference growth.

The ProVIDe trial randomised 434 ELBW babies in six New Zealand and two Australian level 3 neonatal intensive care units to receive either an additional 1 g.d^-1^ per day of intravenous amino acids or placebo in the first 5 days after birth in addition to standard nutritional support [28]. The primary outcome of the trial is survival free from neurodevelopmental disability at 2 years’ corrected age (CA), expected to be available in 2021. Baseline nutritional intakes were not mandated, meaning that participants in both the intervention and placebo groups received a range of nutrient intakes due to the differences in unit nutrition policies. We report a cohort analysis investigating the relationships between macronutrient intakes in the first 4 weeks after birth, and growth from birth to 4 weeks of age and 36 weeks’ CA.

We hypothesised that:

1. Intakes of macronutrients in the first 4 weeks after birth are associated with changes in weight, length and head circumference z scores
2. Relationships between nutritional intakes and growth are not different for girls and boys
3. Growth outcomes differ across hospital sites in accordance with nutrition practices

## 2. Materials and Methods

### 2.1. Study Population

This cohort comprises ELBW babies who participated in the ProVIDe trial (Australian New Zealand Clinical Trials Registry: ACTRN12612001084875), a multicentre, parallel, two-arm, double-blind, randomized, controlled trial. The Northern B Health and Disability Ethics Committee gave ethical approval for the study (No 13/NTB/84) and each participating site had institutional approval through local institutional review processes. Informed written consent was obtained for all participants from their parents or guardians. Participants were recruited between 29 April 2014 and 30 October 2018. The ProVIDe study protocol has been published elsewhere [28]. Briefly, in addition to standard nutritional support according to each participating unit’s policies, participants were randomized at 1:1 ratio to receive either 1 g per day of protein as amino acid solution (TrophAmine^®^, B Braun Medical, Irvine, CA, USA), or placebo (0.45% saline providing 0.9 mmol/day sodium) administered through the umbilical arterial catheter for the first 5 days after birth. The placebo was chosen as it is the most commonly used umbilical arterial fluid used in New Zealand and Australian neonatal units. Biochemical monitoring was as per unit policy / attending physician request and these data will be reported separately. Inclusion criteria were birthweight <1000 g and placement of an umbilical arterial catheter. Exclusion criteria were: admission to neonatal intensive care more than 24 h after birth; multiple births of more than 2 babies; known chromosomal or genetic abnormality; congenital disorder affecting growth; inborn error of metabolism, and danger of imminent death.

### 2.2. Data Collection

Nutritional intake from all actual intravenous and enteral products (including the study fluids) and weight data were collected prospectively and used to estimate mean daily intravenous, enteral and total intakes of energy (kcal·Kg^−1^·d^−1^), macronutrients (protein, fat and carbohydrates, g·Kg^−1^·d^−1^) and energy: protein ratio, and total fluid, enteral volume and breastmilk volume (mL·Kg^−1^·d^−1^) from birth until 4 weeks of age. Intakes on the day of birth and day of death were excluded as they did not represent a full 24 h intake. Intakes included fluids from medication and albumin infusions but not from other transfused blood products. Total nutrient intakes per day were divided by the birth weight until the birth weight was surpassed, then by the most recent weight. Full enteral feeds were defined as 150 mL·Kg^−1^·d^−1^ or when no further intravenous nutrition was given. Total protein intake was calculated from protein equivalent for intravenous amino acids plus enteral protein [29]. Nutrient intakes were calculated using recommended values [29], manufacturers’ composition data and estimates of expressed breastmilk composition [30] and separate donor milk values [31], combining data for weeks 1–2 and of weeks 2–4 for ease of analysis (Table 1). 

Weight, length and head circumference were measured at birth, 28 days, 8 weeks (± 7 days), 36 weeks’ CA, and discharge (both ± 10 days) by trained staff using a Harpenden (Holtain Ltd., Dyfed, Wales, UK) or similar neonatometer and non-stretch teflon measuring head tapes (Seca, Protec Solutions Ltd., Wellington, New Zealand) [32,33], and converted to z-scores using the Fenton growth charts [34]. For babies born at <23 weeks, z scores were obtained by linear extrapolation for head growth [35] and by decreasing rate of change for head circumference and length [34]. Growth velocity (GV) was calculated using: GV = [1000 × ln(Wn/W1)]/(Dn-D1) where W = weight (g) and D = day after birth on days *n* (end of time interval) and 1 (beginning of time interval) [36] and was treated as missing if the baby had no weight recorded on the first and last day of each week.

Optimal growth has been described as a z-score change from birth of between −0.8 and 0.8, to allow for the physiological postnatal weight loss due to contraction of extracellular water and some regression to genetically determined growth [37]. Expected growth was therefore defined as change in z-score on the Fenton growth charts [34] between −0.8 and 0.8 from birth to either 4 weeks or 36 weeks’ CA. Target growth was defined as change in z-score from birth to 36 weeks’ CA of between -0.8 and 0.8 in all three of weight, length and head circumference. For analyses to examine the possibility that higher enteral intakes are a marker for a “well” baby, a “well” baby was defined as one with no patent ductus arteriosus, necrotising enterocolitis, chronic lung disease or culture proven late sepsis.

### 2.3. Statistical Analysis

To calculate median nutrient intakes, only babies who had survived for at least 3 days of week 1 were included. For the other calculations all babies’ intakes were included. Analyses comparing different brands of intravenous amino acid solutions were adjusted for IV protein intake in week 1 to control for the study intervention.

Analyses were undertaken using SAS version 9.4 (SAS Institute Inc., Cary, NC, USA). Statistical tests were two-sided at a 5% significance level. Data are presented as median (range or interquartile range), mean (SD) and number (%). Two-sample t-test was used to compare the distribution of continuous variables between groups, and the chi-square test was used on categorical variables. Relationships between growth and nutrient and fluid intake were first tested using Spearman correlation coefficients, and then explored using linear and logistic regression models adjusted for hospital site, sex, gestational age at birth and birthweight z-score. For comparisons of growth amongst hospitals, the Tukey–Kramer adjustment for multiple comparisons was considered. 

## 3. Results

**Figure 1:**
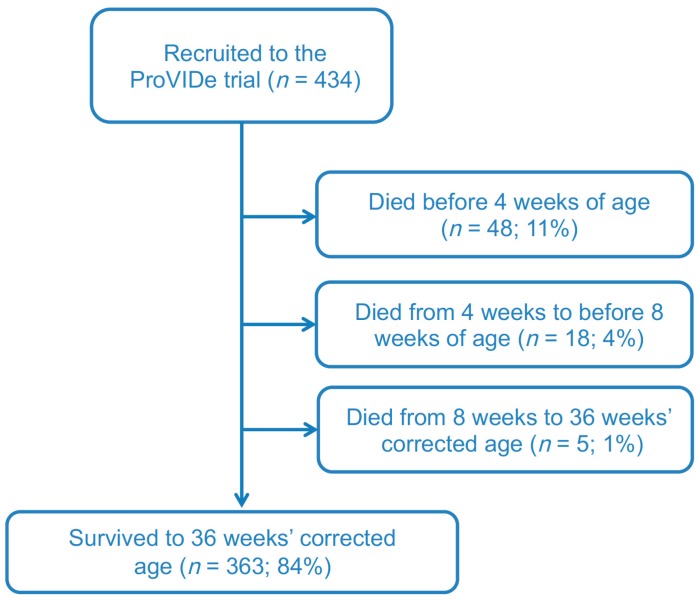
ProVIDe STROBE Flow Diagram.

Baseline data were collected from a total of 434 eligible ProVIDe babies at birth. No participants were withdrawn from the study. Of the 434 babies, 382 (88%) were recruited in New Zealand (NZ) and 52 (12%) in Australia. Of these, 388 survived to 4 weeks, 373 to 8 weeks, and 364 to 36 weeks’ CA and also had growth measurements within the window specified (Figure 1). 

Of the included babies, 49% (*n* = 212) were boys, 11% (*n* = 48) were SGA and 22% (*n* = 94) were twins (Table 2).

**Figure 2:**
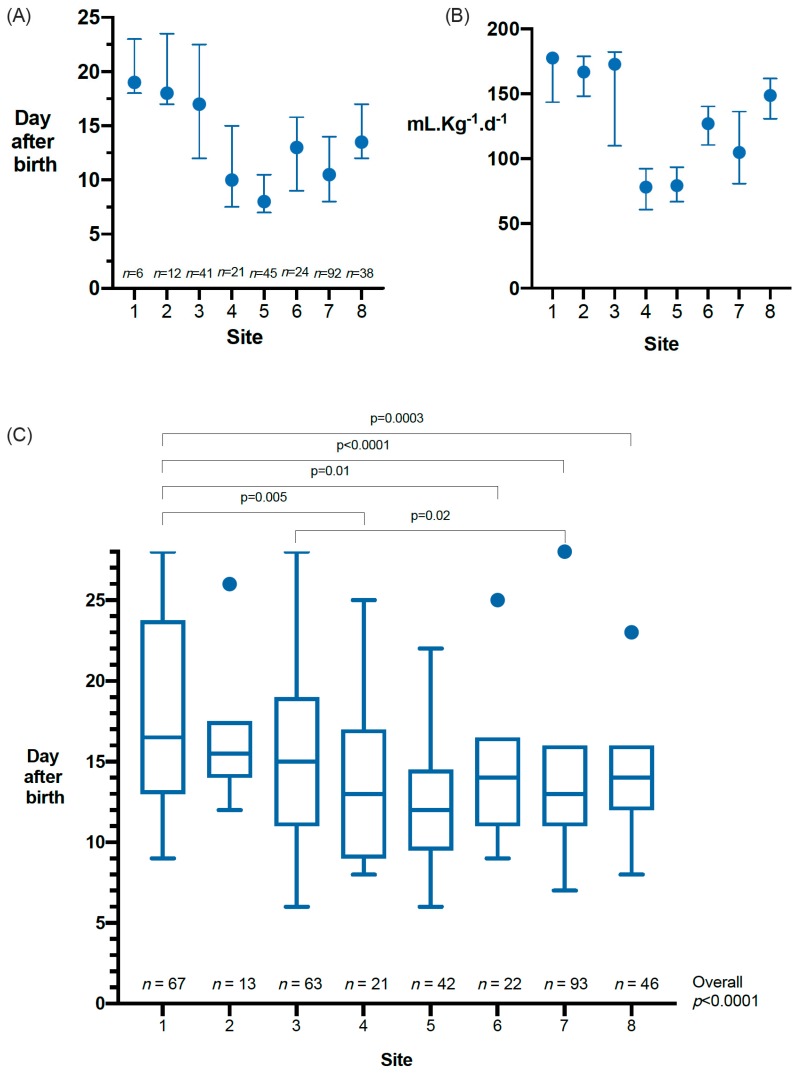
Enteral feeding and timing of breastmilk fortifier addition in participating sites. (**A**) Day after birth of the first fortified feed or preterm formula *n = 279.* (**B**) Feed volume in mL·Kg^−1^·d^−1^ at the time of the first fortified feed or preterm formula *n = 279.* (**C**) Days to full enteral feeding (defined as the day when no further intravenous nutrition was given or 150 mL·Kg^−1^·d^−1^ enteral feeds was reached). Bottom and the top of the box, the first and third quartiles; band inside the box, median; whiskers, 1.5 times the interquartile range, and circles, outliers. *n* = 367, 18 babies excluded (**C**) due to missing data on time from birth to full enteral feeds.

In week 1, 99.5% of babies had only expressed breastmilk as their enteral feed, reducing to 92%, 89% and 89% in weeks 2, 3 and 4 respectively; the remainder had some preterm or standard term formula. Breastmilk fortifiers were added at different time points in seven sites and not routinely used at one site (Figure 2A,B). The median (IQR) day of commencement of fortifier or preterm formula was 12 (9, 17) at a median (IQR) enteral feed volume of 113 (80, 153) mL·Kg^−1^·d^−1^. Full fortifier or preterm formula was not started during the 4 weeks after birth for 35% of babies (sites 1–8: 92%; 20%; 47%; 13%; 8%; 14%; 13%, and 30%). The median (IQR) age at achievement of full enteral feeds was 14 (11, 17) days and this differed by site (Figure 2C).

**Figure 3:**
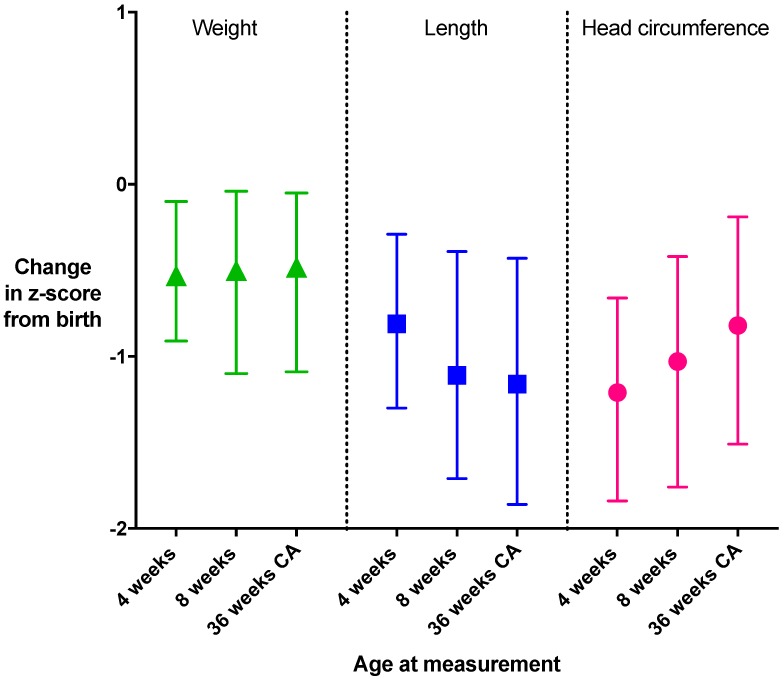
Change in z-score from birth for weight, length and head circumference. Change in z-score for the overall cohort at each time point from birth (median and interquartile range). *n*: 4 weeks, weight *n* = 344, length *n* = 323, head circumference *n* = 339; 8 weeks, weight *n* = 343, length *n* = 334, head circumference *n* = 339; 36 weeks’ corrected age, *n* = 369, length *n* = 346, head circumference *n* = 339. Expected growth (z-score change >-0.8 to <0.8) is indicated by the grey shaded area. CA, corrected age.

From birth to 36 weeks’ CA, the median (IQR) change in z-score for weight was −0.48 (−1.09, 0.05), for length −1.16 (−1.86, −0.43), and for head circumference −0.82 (−1.51, −0.19) (Figure 3).

**Figure 4:**
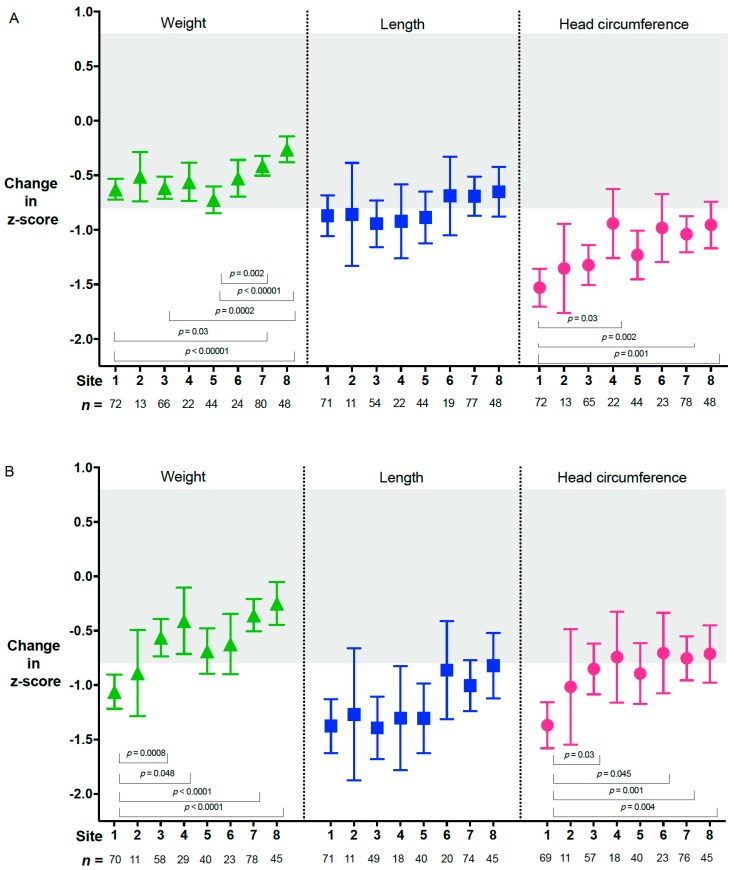
Change in z-score from birth for each site. Change in z-score from birth to 4 weeks (**A**) and 36 weeks’ corrected age (**B**) for each site, adjusted for site, sex, gestational age at birth and birthweight z-score. Data are median and interquartile range. Expected growth (z-score change >−0.8 to <0.8) is indicated by the grey shaded area. CA, corrected age.

These changes also varied amongst sites (Figure 4A,B). The greatest fall in z-scores for length and head circumference occurred in the first 4 weeks after birth (Figure 3) and, for length, persisted to 36 weeks’ CA (Figure 4B)

**Figure 5:**
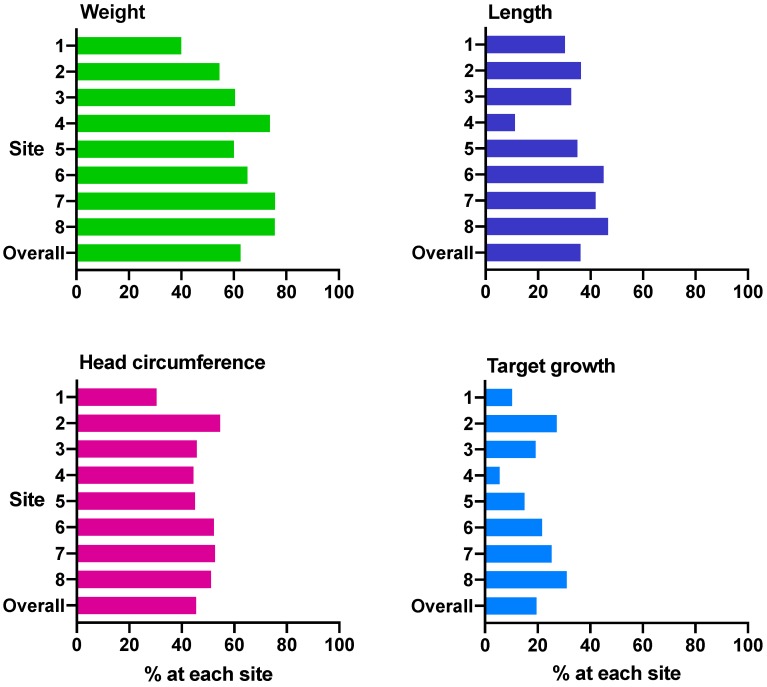
Percentage of babies at each site with expected growth from birth 36 weeks’ corrected age. (**A**) weight (*n* = 369), (**B**) length (*n* = 346), (**C**) head circumference (*n* = 339) and (**D**) target growth (*n* = 335). Expected growth is change in z-score from −0.8 to 0.8, target growth is change in z-score from −0.8 to 0.8 for all three of weight, length and head circumference.

SGA babies were more likely to achieve expected growth at 36 weeks’ CA than those who were appropriate for gestational age (AGA): weight 82.9% vs. 59.7% (*p* = 0.004), length 48.7% vs. 34.5% (*p* = 0.08), and head circumference 67.5% vs. 42.5% (*p* = 0.003). Overall, 19.6% of the cohort achieved target growth (Figure 5), and this proportion was similar in SGA and AGA babies (26.8% vs. 18.6%, *p* = 0.21).

**Figure 6:**
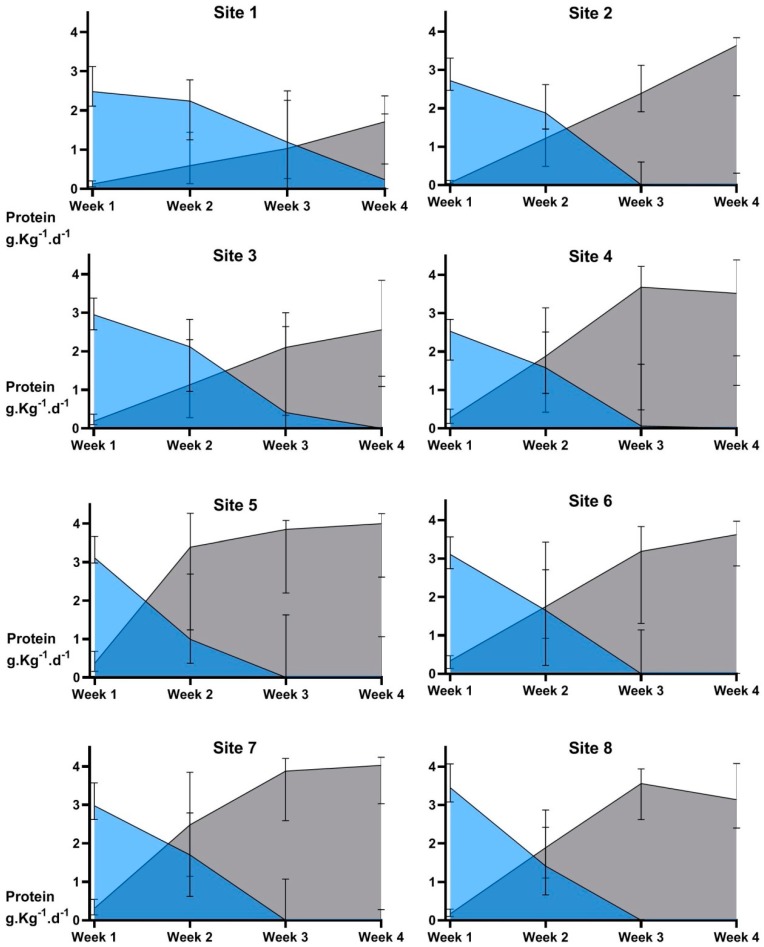
Site differences in intravenous and enteral protein intake over the first 4 weeks. Data are median and interquartile range, *n* = 367. Intravenous protein intakes (blue shading) were significantly different amongst sites in week 1 (*p* < 0.0001), week 2 (*p* = 0.04) and week 3 (*p* < 0.0001). Enteral protein intakes (grey shading) were significantly different amongst sites in each of weeks 1 to 4 (all *p* < 0.0001).

Nutrient intakes in the first 4 weeks are shown in Table 3. There were significant differences amongst sites for intravenous and enteral protein intake (Figure 6). Ammonia concentrations and their relationship with protein intake and postnatal age have been reported previously [38]. Three sites did not commence fortified feeds or give preterm formula until a mean of 17 days or more after birth, Figure 2A. These sites had the lowest median protein intakes (<3.5 g·Kg^−1^·d^−1^, Figure 6), and greatest fall in head circumference z-score at 4 weeks (Figure 4A). Intakes of intravenous and enteral fluid, breastmilk and macronutrients were not significantly different between boys and girls (data not shown) and there was no significant difference between the number of boys and girls classified as “well” (10.6 vs. 14.5%, *p* = 0.25). Achievement of expected growth for weight, length and head circumference at 36 weeks’ CA was similar for boys and girls, but fewer boys achieved target growth (15.3% vs. 23.6%, *p* = 0.06). Changes in z-scores to 36 weeks’ CA for weight, length and head circumference were not associated with the brand of amino acid solution in either adjusted or unadjusted analysis (data not shown).

In the adjusted linear regression, there were significant associations between nutrient intakes in the first 4 weeks and growth at 4 weeks of age and 36 weeks’ CA. Each 1 g·Kg^−1^·d^−1^ total protein in week 2 was associated with a 0.26 z-score increase in head circumference at 36 weeks’ CA (Supplementary Table S1).

**Figure 7:**
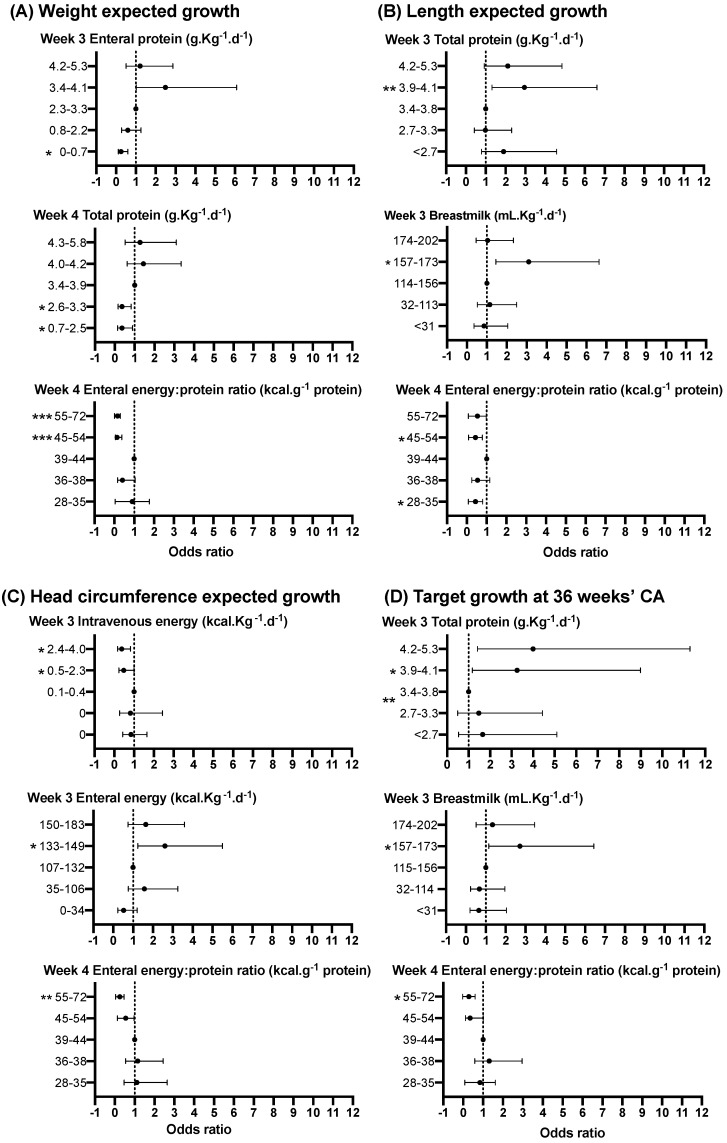
Relationship between selected nutrient intakes in each of the first 4 weeks and expected growth for weight (**A**), length (**B**) and head circumference (**C**), and target growth (**D**). Data are adjusted OR and 95% confidence interval (CI) of achieving expected or target growth for each quintile of intake, where quintile 3 is the referent. Significant *p* values are shown as *<0.05, **<0.01, ***<0.001. For full details, see Supplementary Table S2.

In the third week after birth, babies receiving mean total protein intakes in the highest quintile had 4-fold higher odds of achieving target growth compared to babies in the middle quintile (Figure 7D, Supplementary Table S2). Consistent with this, babies receiving enteral protein intakes in the lowest quintile had 80% lower odds of achieving target growth than babies receiving enteral protein intake in the middle quintile. Similarly, babies who had breastmilk volumes in the 4th quintile were more likely to achieve target growth than those in the middle quintile (Figure 7D, Supplementary Table S2).

The strongest positive associations with change in head circumference z-score at 36 weeks’ CA were for mean total protein intake in weeks 2, 3 and 4. In week 2, babies with a lower total protein intake than those in the middle quintile had less than half the odds of achieving expected head circumference growth from birth to 36 weeks’ CA (Supplementary Table S2). By week 3, intravenous protein intake was negatively associated with z-score change at 36 weeks’ CA for weight, length and head circumference (Supplementary Table S1). 

Babies in both high (4^th^ quintile 45–54 kcal·g^−1^) and low (1^st^ quintile 28–35 kcal·g^−1^) quintiles of enteral energy to protein ratio were less likely to achieve expected length growth at 36 weeks’ CA when compared to the middle quintile (39–44 kcal·g^−1^, Supplementary Table S2).

In adjusted analyses (but without adjusting for site), babies who commenced fully fortified breastmilk or preterm formula in the first 4 weeks were more likely to achieve expected growth for weight at 4 weeks (OR 1.9 CI 1.0–3.5 *p* = 0.04) and at 36 weeks’ CA (OR 4.9 CI 2.9-8.5 *p* = 0.0001), for head circumference at 4 weeks (OR 2.7 CI 1.5–4.7 *p* = 0.0006) and 36 weeks’ CA (OR 3.5 CI 2.0-5.9 *p* < 0.0001) and target growth (OR 3.0 CI 1.4–6.2 *p* = 0.004) compared with babies who had unfortified breastmilk or standard term formula for the first 28 days. The odds of achieving expected growth for weight at 36 weeks CA decreased by 10% for each day later that fortifier was started (OR 0.9 CI 0.9-1.0 *p* = 0.04). There was no significant association between the time or feed volume when fortifier or preterm formula was commenced and achieving expected growth for length, head circumference or target growth at 4 weeks or 36 weeks’ CA.

## 4. Discussion

This multicentre cohort analysis shows nutritional intake in the first 4 weeks is correlated with growth at both 4 weeks of age and 36 weeks’ CA. Protein is more strongly correlated with growth than other macronutrients and protein intake in week 2 is most strongly correlated with head circumference growth. Protein intake may fall significantly in the second week after birth during the transition from intravenous to enteral feeds, if breastmilk is unfortified [39]. For instance, unfortified breastmilk at 150 mL·Kg^−1^·d^−1^ supplies only 2 g·Kg^−1^·d^−1^ protein in contrast to 3.5 to 4.1 g·Kg^−1^·d^−1^ for fully fortified breastmilk.

Our results are consistent with a Cochrane meta-analysis (*n* = 315) of higher vs. lower intravenous amino acid intake which found improved head circumference growth from birth to NICU discharge in the higher protein intake group [10] but not with another meta-analysis of 5 different studies (*n* = 316) that included both low vs. high-dose and early vs. late intravenous amino acid administration in very low birthweight babies, and concluded there was no significant difference in mean head circumference at 36 weeks’ CA between amino acid intake groups [27]. The trials in these meta-analyses all had a relatively low sample size (<170), were single centre and mostly failed to achieve the planned amino acid intake difference between intervention and control groups. Three randomised controlled trials have reported worse head circumference growth in higher amino acid groups [40,41,42], but each of these trials had low energy: protein ratio (mean ≤25 kcal·g^−1^) in the first 5 days after birth. These three studies may indicate that higher protein intake in the presence of low energy intakes is detrimental for head growth. However, we did not find any significant correlations between growth and energy: protein ratio until week 3 when low total energy: protein ratio (18–32) was associated with worse head circumference growth and higher total protein intakes were associated with higher head circumference. Nutritional intakes varied significantly amongst sites as we did not mandate baseline nutritional intakes. Thus, site policies and practices, such as use and timing of initiation of fortifier, composition of standard solutions and timing of initiation and speed of advancement of enteral feeding all affected intake. However, the magnitude of these differences across sites reflect those reported in international surveys of nutrition practices [43,44]. Despite significantly different macronutrient intakes in week 1 within and amongst sites, we found no associations between nutritional intake in the first week after birth and growth at 4 weeks or 36 weeks’ CA. This suggests that nutritional intake in week 1 may be a less important determinant of later growth than in later periods and may help to explain the inconclusive findings in neonatal nutrition research focusing on nutritional intake in week 1.

Positive correlations for total and enteral protein in weeks 2, 3 and 4 and negative correlations for intravenous protein in weeks 3 and 4 with head circumference growth at 36 weeks’ CA in our cohort are consistent with previous findings that enteral protein is positively associated with larger total brain volume, and that longer duration of intravenous nutrition is associated with smaller total brain volume, cerebellar, basal ganglia and thalami volumes, as well as cortical grey matter volume [26]. In a previous study of babies born at <1500 g or <31 weeks’ gestation and followed up at 7 years, we found higher enteral protein, fat and carbohydrate intakes in the first 7 days, and volume of breastmilk intake in the first 14 days, were associated with reduced odds of neurodevelopmental impairment [45]. While we cannot exclude the possibility that babies who received intravenous nutrition for longer and had less enteral nutrition were more unwell, impacting upon their growth, the significant differences in nutritional intakes and growth amongst sites in this cohort suggest that “unwellness” is not the major cause of faltering growth.

In this cohort of babies for whom the clinical team elected to insert a UAC, one site achieved target growth in 31% of the babies, but another site achieved this in only 6%. The sites with the lowest median protein intakes and longest time to full enteral feeding also had the greatest fall in head circumference z-score at 4 weeks. These data suggest closer attention to early nutritional intakes may reduce the decreases in length and head circumference z-scores that occur in the first 4 weeks after birth. Higher enteral energy, protein and breastmilk volume in weeks 3 to 4 were all associated with better linear and head circumference growth and may be the key to achieving better growth in these groups. 

A potential weakness of this study is that the predominant use of expressed breastmilk in the first 4 weeks means that the enteral energy and macronutrient intakes from breastmilk were estimates. Growth measurements were taken at different sites by different people, although standardised neonatometers, non-stretch measuring head tapes and training were provided to all sites as part of the ProVIDe RCT. It is also possible that the recovery in head circumference growth we observed between 4 weeks and 36 weeks’ CA may be partly due to changes in head shape caused by a combination of CPAP head-gear (causing dolichocephaly) and the inability of preterm babies to voluntarily change head position (leading to plagiocephaly) [46].

Strengths of this study are that it is a multicentre cohort with detailed nutritional intake and growth data collected prospectively as part of an RCT using standardised data collection and growth measurement processes. While associations between nutrition and growth do not prove causation, the sample size is large in comparison with previously published cohort studies and meta-analyses in ELBW babies, allowing subgroup analysis by sex. The participants originate from eight different sites in two countries making the findings widely generalisable.

## 5. Conclusions

In ELBW babies receiving a range of protein intakes up to 5 g·Kg^−1^·d^−1^, faltering weight and head circumference growth primarily occurs in the first 4 weeks after birth, but length faltering continues up to 36 weeks’ CA, emphasising the importance of monitoring linear and head circumference growth as well as weight gain.

Positive correlations between enteral, and negative correlations between intravenous, protein intakes and weight, length and head circumference growth at 36 weeks’ CA suggest full enteral breastmilk feeding with sufficient protein from week 2 may improve growth. Differences in nutrition and growth outcomes amongst sites indicate there may be potential to improve growth with enhanced nutrition practices. Neurodevelopmental follow up of the cohort at 2 years CA is underway as part of the ProVIDe trial, and will allow further investigation of the impact of early nutritional intake on later growth and neurodevelopment.

## Figures and Tables

**Table 1:** Breastmilk composition used for calculations.

Per 100 mL	Protein (g)	Carbohydrate (g)	Fat (g)	Energy (kcal)
Mother’s expressed breastmilk—day 1–4	1.83	5.98	3.22	67.35
Mother’s expressed breastmilk—day 15–28	1.35	6.05	3.86	72.95
Donor breastmilk	0.90	6.60	4.00	66.00

**Table 2:** Baseline demographic and clinical characteristics of the cohort.

Characteristic	At birth
**For mother**:	
Age in years	31 (16, 44)
Caesarean section	234 (54)
Antenatal corticosteroids (any)	410 (94)
Maternal diabetes	24 (6)
**For baby:**	
Gestation (weeks)	25.7 (22.7, 31.5)
Birthweight (g)	777 (405, 998)
Birthweight z score	0.06 (−2.46, 2.64)
Birth length (cm)	33.0 (26.0, 39.0)
Birth length z score	−0.09 (−4.08, 2.40)
Birth head circumference (cm)	23.4 (18.3, 27.0)
Birth head circumference z score	0.05 (−3.34, 2.34)
Small-for-gestational-age	48 (11%)
Male	212 (49%)
Singleton	340 (78%)

Data are median (range) and *n* (%). For birthweight *n* = 434, length *n* = 413 and head circumference *n* = 431.

**Table 3:** Total, intravenous and enteral fluid and macronutrient intakes in the first 4 weeks.

Nutrition	Week 1	Fortnight 1	Month 1
*Fluid volumes (ml·Kg^−1^·d^−1^)*			
**Total**	139 (125, 151)	151 (143, 161)	158 (152, 167)
Intravenous	123 (106, 149)	105 (77, 133)	66 (44, 110)
Enteral	11 (5, 23)	44 (16, 73)	93 (46, 117)
Breastmilk	11 (5, 23)	42 (15, 72)	87 (38, 115)
*Macronutrients*			
**Total energy (kcal·Kg^−1^·d^−1^)**	76 (70, 83)	92 (83, 102)	109 (95, 122)
Intravenous	66 (58, 73)	57 (42, 70)	36 (24, 57)
Enteral	8 (4, 16)	31 (11, 56)	73 (36, 96)
**Total protein (g·Kg^−1^·d^−1^)**	3.34 (2.86, 3.76)	3.48 (3.01, 3.90)	3.50 (2.95, 3.92)
Intravenous	2.99 (2.50, 3.45)	2.40 (1.85, 2.40)	1.46 (1.04, 2.24)
Enteral	0.20 (0.01, 0.43)	0.88 (0.27, 1.66)	1.82 (0.75, 2.63)
**Total carbohydrate (g·Kg^−1^·d^−1^)**	10.33 (9.13, 11.79)	11.70 (10.88, 12.58)	12.62 (11.72, 13.56)
Intravenous	9.43 (7.97, 11.11)	8.13 (5.95, 10.51)	5.33 (3.51, 8.44)
Enteral	0.67 (0.32, 1.41)	2.90 (0.95, 5.34)	6.61 (3.10, 9.34)
**Total fat (g·Kg^−1^·d^−1^)**	2.57 (2.21, 3.01)	3.45 (2.84, 3.95)	4.56 (3.67, 5.16)
Intravenous	2.06 (1.73, 2.43)	1.76 (1.30, 1.76)	1.07 (0.73, 1.73)
Enteral	0.36 (0.17, 0.75)	1.46 (0.54, 2.46)	3.50 (1.70, 4.40)
**Total energy: protein ratio (kcals/g)**	23 (20, 28)	27 (24, 31)	32 (29, 36)
Intravenous	22 (19, 27)	24 (20, 29)	24 (21, 30)
Enteral	37 (37, 37)	37 (35, 38)	39 (36, 44)

Data are median (IQR). Data collected on Day 0 and day of death were excluded. Data were included if available for a minimum 3 days in assessment period. *n* = 425.

## Supplementary Materials

The following are available online at https://www.mdpi.com/2072-6643/12/3/760/s1, Table S1: Relationship between nutrient intakes in each of the first 4 weeks and change in z-score from birth; Table S2A: Relationship between selected nutrient intakes in each of the first 4 weeks and expected growth (weight, length and head circumference), and target growth; Table S2B: Quintiles of nutrient intakes in each of the first four weeks.
