# Supplementary material for: Relationships between Neonatal Nutrition and Growth to 36 Weeks’ Corrected Age in ELBW Babies–Secondary Cohort Analysis from the Provide Trial
Source: Nutrients. 2020 Mar 13;12(3):760. doi: 10.3390/nu12030760 (PMC7146349; doi:10.3390/nu12030760)
Supplement: Supplementary file 1 [file nutrients-12-00760-s001.zip › Nutrients Growth Supplementary Table 1.docx]

**Supplementary Table 1**Relationship between nutrient intakes in each of the first 4 weeks and change in z-score from birth

**Weight**

| **Predictor** | | | **Birth to 4 weeks** | | | | **Birth to 36 weeks’ CA** | | | | |
| --- | --- | --- | --- | --- | --- | --- | --- | --- | --- | --- | --- |
| **Fluid (per 100 mL.Kg^-1^.d^-1^)** | | | **Week 1** | **Week 2** | **Week 3** | **Week 4** | | **Week 1** | **Week 2** | **Week 3** | **Week 4** |
| Total | *B* coefficient | | -0.23 | -0.28 | -0.03 | -0.30 | | -0.35 | 0.26 | 0.58 | 0.69 |
|  | Lower CI | | -0.55 | -0.62 | -0.29 | -0.55 | | -0.92 | -0.31 | 0.15 | 0.26 |
|  | Upper CI | | 0.10 | 0.05 | 0.24 | -0.04 | | 0.23 | 0.82 | 1.02 | 1.13 |
|  | *p* value | | 0.18 | 0.09 | 0.84 | **0.02** | | 0.24 | 0.37 | **0.01** | **<0.01** |
| Intravenous | *B* coefficient | | -0.10 | -0.01 | 0.01 | 0.00 | | -0.27 | -0.20 | -0.34 | -0.36 |
|  | Lower CI | | -0.35 | -0.11 | -0.08 | -0.09 | | -0.67 | -0.35 | -0.47 | -0.51 |
|  | Upper CI | | 0.14 | 0.08 | 0.09 | 0.09 | | 0.14 | -0.05 | -0.20 | -0.21 |
|  | *p* value | | 0.39 | 0.75 | 0.89 | 0.95 | | 0.20 | **0.01** | **<0.0001** | **<0.0001** |
| Enteral | *B* coefficient | | -0.03 | -0.01 | -0.01 | -0.02 | | 0.17 | 0.19 | 0.32 | 0.32 |
|  | Lower CI | | -0.36 | -0.09 | -0.08 | -0.10 | | -0.38 | 0.05 | 0.20 | 0.20 |
|  | Upper CI | | 0.30 | 0.08 | 0.07 | 0.05 | | 0.71 | 0.34 | 0.45 | 0.45 |
|  | *p* value | | 0.85 | 0.89 | 0.85 | 0.53 | | 0.55 | **0.01** | **<0.0001** | **<0.0001** |
| Breastmilk | *B* coefficient | | -0.05 | -0.04 | -0.05 | -0.06 | | 0.16 | 0.15 | 0.21 | 0.17 |
|  | Lower CI | | -0.38 | -0.13 | -0.13 | -0.13 | | -0.38 | 0.00 | 0.09 | 0.05 |
|  | Upper CI | | 0.28 | 0.05 | 0.02 | 0.01 | | 0.71 | 0.29 | 0.33 | 0.29 |
|  | *p* value | | 0.77 | 0.39 | 0.14 | 0.10 | | 0.56 | **0.05** | **<0.001** | **<0.01** |
| **Energy (per 10 Kcal.Kg^-1^.d^-1^)** | | |  |  |  |  | |  |  |  |  |
| Total | *B* coefficient | | 0.03 | 0.01 | 0.01 | 0.00 | | 0.06 | 0.06 | 0.08 | 0.08 |
|  | Lower CI | | -0.03 | -0.01 | -0.01 | -0.02 | | -0.02 | 0.02 | 0.05 | 0.05 |
|  | Upper CI | | 0.08 | 0.04 | 0.03 | 0.02 | | 0.15 | 0.10 | 0.11 | 0.11 |
|  | *p* value | | 0.33 | 0.27 | 0.15 | 0.96 | | 0.15 | **<0.01** | **<0.0001** | **<0.0001** |
| Intravenous | *B* coefficient | | 0.03 | 0.00 | 0.01 | 0.01 | | 0.03 | -0.03 | -0.06 | -0.06 |
|  | Lower CI | | -0.02 | -0.01 | -0.01 | -0.01 | | -0.06 | -0.06 | -0.08 | -0.09 |
|  | Upper CI | | 0.09 | 0.02 | 0.02 | 0.02 | | 0.11 | 0.00 | -0.03 | -0.03 |
|  | *p* value | | 0.21 | 0.71 | 0.45 | 0.56 | | 0.53 | **0.03** | **<0.0001** | **<0.0001** |
| Enteral | *B* coefficient | | -0.01 | 0.00 | 0.00 | 0.00 | | 0.03 | 0.03 | 0.04 | 0.04 |
|  | Lower CI | | -0.05 | -0.01 | -0.01 | -0.01 | | -0.05 | 0.01 | 0.03 | 0.03 |
|  | Upper CI | | 0.04 | 0.01 | 0.01 | 0.01 | | 0.10 | 0.04 | 0.06 | 0.06 |
|  | *p* value | | 0.83 | 0.80 | 0.82 | 0.75 | | 0.50 | **0.01** | **<0.0001** | **<0.0001** |
| **Protein (per 1 g.Kg^-1^.d^-1^)** | | |  |  |  |  | |  |  |  |  |
| Total | *B* coefficient | | 0.11 | 0.10 | 0.13 | 0.08 | | 0.06 | 0.21 | 0.15 | 0.26 |
|  | Lower CI | | 0.03 | 0.02 | 0.06 | 0.01 | | -0.08 | 0.07 | 0.04 | 0.15 |
|  | Upper CI | | 0.19 | 0.19 | 0.19 | 0.14 | | 0.19 | 0.35 | 0.26 | 0.37 |
|  | *p* value | | **0.01** | **0.02** | **<0.001** | **0.02** | | 0.42 | **<0.01** | **0.01** | **<0.0001** |
| Intravenous | *B* coefficient | | 0.09 | 0.01 | 0.02 | 0.02 | | 0.02 | -0.06 | -0.14 | -0.14 |
|  | Lower CI | | 0.02 | -0.03 | -0.02 | -0.02 | | -0.10 | -0.13 | -0.21 | -0.20 |
|  | Upper CI | | 0.16 | 0.05 | 0.05 | 0.06 | | 0.14 | 0.00 | -0.08 | -0.07 |
|  | *p* value | | **0.01** | 0.67 | 0.41 | 0.45 | | 0.74 | 0.06 | **<0.0001** | **<0.0001** |
| Enteral | *B* coefficient | | -0.02 | 0.01 | 0.02 | 0.01 | | 0.10 | 0.08 | 0.16 | 0.16 |
|  | Lower CI | | -0.17 | -0.02 | -0.01 | -0.02 | | -0.14 | 0.03 | 0.10 | 0.11 |
|  | Upper CI | | 0.12 | 0.04 | 0.06 | 0.04 | | 0.34 | 0.14 | 0.21 | 0.21 |
|  | *p* value | | 0.75 | 0.56 | 0.24 | 0.63 | | 0.42 | **<0.01** | **<0.0001** | **<0.0001** |
| **Fat (per 1 g.Kg^-1^.d^-1^)** | | |  |  |  |  | |  |  |  |  |
| Intravenous | | B coefficient | 0.11 | 0.01 | 0.03 | 0.03 | | 0.15 | -0.09 | -0.16 | -0.19 |
|  | | Lower CI | -0.01 | -0.04 | -0.01 | -0.02 | | -0.06 | -0.17 | -0.23 | -0.27 |
|  | | Upper CI | 0.24 | 0.06 | 0.08 | 0.07 | | 0.36 | 0.00 | -0.08 | -0.11 |
|  | | p value | 0.08 | 0.57 | 0.13 | 0.31 | | 0.17 | **0.04** | **<.0001** | **<.0001** |
| Enteral | | *B* coefficient | -0.01 | 0.00 | 0.00 | -0.01 | | 0.05 | 0.06 | 0.08 | 0.08 |
|  | | Lower CI | -0.11 | -0.03 | -0.02 | -0.03 | | -0.12 | 0.02 | 0.05 | 0.05 |
|  | | Upper CI | 0.09 | 0.03 | 0.02 | 0.01 | | 0.22 | 0.10 | 0.11 | 0.11 |
|  | | *p* value | 0.88 | 0.99 | 0.89 | 0.55 | | 0.55 | **0.01** | **<0.0001** | **<0.0001** |

| **Carbohydrate (per 1 g.Kg^-1^.d^-1^)** | |  |  |  |  |  |  |  |  |
| --- | --- | --- | --- | --- | --- | --- | --- | --- | --- |
| Intravenous | *B* coefficient | 0.01 | 0.00 | 0.00 | 0.00 | 0.00 | -0.02 | -0.04 | -0.04 |
|  | Lower CI | -0.02 | -0.01 | -0.01 | -0.01 | -0.04 | -0.04 | -0.05 | -0.05 |
|  | Upper CI | 0.04 | 0.01 | 0.01 | 0.01 | 0.05 | 0.00 | -0.02 | -0.02 |
|  | *p* value | 0.65 | 0.93 | 0.86 | 0.75 | 0.84 | **0.03** | **<0.0001** | **<0.0001** |
| Enteral | *B* coefficient | 0.0 | 0.00 | 0.00 | 0.00 | 0.00 | 0.03 | 0.04 | 0.04 |
|  | Lower CI | -0.1 | -0.01 | -0.01 | -0.01 | -0.04 | 0.01 | 0.03 | 0.03 |
|  | Upper CI | 0.0 | 0.01 | 0.01 | 0.01 | 0.05 | 0.04 | 0.06 | 0.06 |
|  | *p* value | 0.8 | 0.55 | 0.46 | 0.89 | 0.84 | **<0.01** | **<0.0001** | **<0.0001** |
| **Energy:protein ratio  (per Kcal.g^-1^ protein)** | |  |  |  |  |  |  |  |  |
| Intravenous | *B* coefficient | 0.00 | 0.00 | 0.00 | 0.00 | -0.01 | -0.01 | 0.00 | -0.01 |
|  | Lower CI | -0.01 | -0.01 | 0.00 | -0.02 | -0.01 | -0.01 | 0.00 | -0.03 |
|  | Upper CI | 0.00 | 0.00 | 0.00 | 0.01 | 0.00 | 0.00 | 0.01 | 0.01 |
|  | *p* value | 0.11 | 0.52 | 0.89 | 0.52 | 0.25 | 0.10 | 0.61 | 0.41 |
| Enteral | *B* coefficient | 0.02 | -0.01 | -0.01 | -0.01 | -0.01 | -0.02 | -0.03 | -0.03 |
|  | Lower CI | -0.01 | -0.02 | -0.02 | -0.02 | -0.07 | -0.03 | -0.04 | -0.04 |
|  | Upper CI | 0.05 | 0.01 | -0.01 | 0.00 | 0.05 | 0.00 | -0.02 | -0.02 |
|  | *p* value | 0.16 | 0.29 | **<0.001** | **<0.01** | 0.74 | 0.10 | **<0.0001** | **<0.0001** |

**Length**

| **Predictor** | | **Birth to 4 weeks** | | | | **Birth to 36 weeks’ CA** | | | |
| --- | --- | --- | --- | --- | --- | --- | --- | --- | --- |
| **Fluid (per 100 mL.Kg^-1^.d^-1^)** | | **Week 1** | **Week 2** | **Week 3** | **Week 4** | **Week 1** | **Week 2** | **Week 3** | **Week 4** |
| Total | *B* coefficient | 0.46 | 0.17 | 0.27 | 0.42 | 0.26 | 0.00 | 0.53 | 0.44 |
|  | Lower CI | -0.20 | -0.52 | -0.26 | -0.09 | -0.65 | -0.92 | -0.14 | -0.26 |
|  | Upper CI | 1.13 | 0.85 | 0.80 | 0.94 | 1.18 | 0.92 | 1.20 | 1.14 |
|  | *p* value | 0.17 | 0.63 | 0.33 | 0.11 | 0.57 | 0.99 | 0.12 | 0.22 |
| Intravenous | *B* coefficient | 0.20 | -0.04 | -0.26 | -0.32 | -0.07 | -0.13 | -0.36 | -0.30 |
|  | Lower CI | -0.29 | -0.23 | -0.43 | -0.49 | -0.71 | -0.38 | -0.58 | -0.54 |
|  | Upper CI | 0.68 | 0.14 | -0.10 | -0.15 | 0.57 | 0.11 | -0.13 | -0.07 |
|  | *p* value | 0.42 | 0.63 | **<0.01** | **<0.001** | 0.83 | 0.29 | **<0.01** | **0.01** |
| Enteral | *B* coefficient | 0.09 | 0.05 | 0.23 | 0.27 | 0.37 | 0.12 | 0.34 | 0.26 |
|  | Lower CI | -0.59 | -0.12 | 0.08 | 0.13 | -0.51 | -0.11 | 0.14 | 0.06 |
|  | Upper CI | 0.76 | 0.23 | 0.39 | 0.42 | 1.26 | 0.35 | 0.54 | 0.46 |
|  | *p* value | 0.80 | 0.56 | **<0.01** | **<0.001** | 0.41 | 0.31 | **<0.01** | **0.01** |
| Breastmilk | *B* coefficient | 0.04 | 0.04 | 0.19 | 0.21 | 0.34 | 0.02 | 0.19 | 0.15 |
|  | Lower CI | -0.63 | -0.14 | 0.05 | 0.08 | -0.54 | -0.21 | 0.00 | -0.04 |
|  | Upper CI | 0.72 | 0.21 | 0.34 | 0.35 | 1.21 | 0.25 | 0.38 | 0.33 |
|  | *p* value | 0.90 | 0.67 | **0.01** | **<0.01** | 0.45 | 0.88 | 0.05 | 0.12 |
| **Energy (per 10 Kcal.Kg^-1^.d^-1^)** | |  |  |  |  |  |  |  |  |
| Total | *B* coefficient | 0.08 | 0.02 | 0.05 | 0.05 | 0.10 | 0.04 | 0.08 | 0.06 |
|  | Lower CI | -0.02 | -0.04 | 0.01 | 0.02 | -0.04 | -0.03 | 0.03 | 0.01 |
|  | Upper CI | 0.19 | 0.06 | 0.09 | 0.09 | 0.24 | 0.11 | 0.13 | 0.11 |
|  | *p* value | 0.12 | 0.56 | **0.01** | **<0.01** | 0.17 | 0.23 | **<0.01** | **0.01** |
| Intravenous | *B* coefficient | 0.07 | -0.01 | -0.04 | -0.05 | 0.02 | -0.01 | -0.06 | -0.04 |
|  | Lower CI | -0.04 | -0.04 | -0.07 | -0.08 | -0.11 | -0.06 | -0.10 | -0.08 |
|  | Upper CI | 0.18 | 0.03 | -0.01 | -0.02 | 0.16 | 0.03 | -0.02 | 0.00 |
|  | *p* value | 0.19 | 0.73 | **0.01** | **<0.001** | 0.73 | 0.54 | **0.01** | **0.05** |
| Enteral | *B* coefficient | 0.01 | 0.01 | 0.03 | 0.03 | 0.05 | 0.01 | 0.04 | 0.03 |
|  | Lower CI | -0.08 | -0.02 | 0.01 | 0.01 | -0.07 | -0.02 | 0.02 | 0.01 |
|  | Upper CI | 0.10 | 0.03 | 0.05 | 0.05 | 0.17 | 0.04 | 0.06 | 0.05 |
|  | *p* value | 0.80 | 0.63 | **0.01** | **<0.001** | 0.38 | 0.35 | **0.01** | **0.02** |
| **Protein (per 1 g.Kg^-1^.d^-1^)** | |  |  |  |  |  |  |  |  |
| Total | *B* coefficient | 0.16 | 0.08 | 0.06 | 0.05 | 0.12 | 0.22 | 0.13 | 0.17 |
|  | Lower CI | -0.01 | -0.09 | -0.07 | -0.09 | -0.09 | -0.01 | -0.04 | -0.01 |
|  | Upper CI | 0.32 | 0.25 | 0.20 | 0.18 | 0.33 | 0.45 | 0.31 | 0.35 |
|  | *p* value | 0.06 | 0.36 | 0.36 | 0.49 | 0.26 | 0.06 | 0.13 | 0.06 |

| Intravenous | *B* coefficient | | 0.12 | 0.01 | -0.10 | -0.13 | 0.05 | -0.01 | -0.13 | -0.08 |
| --- | --- | --- | --- | --- | --- | --- | --- | --- | --- | --- |
|  | Lower CI | | -0.03 | -0.07 | -0.17 | -0.21 | -0.14 | -0.11 | -0.24 | -0.19 |
|  | Upper CI | | 0.26 | 0.08 | -0.02 | -0.06 | 0.24 | 0.10 | -0.03 | 0.02 |
|  | *p* value | | 0.11 | 0.89 | **0.01** | **<0.001** | 0.58 | 0.93 | **0.01** | 0.13 |
| Enteral | *B* coefficient | | 0.03 | 0.01 | 0.09 | 0.10 | 0.19 | 0.04 | 0.14 | 0.10 |
|  | Lower CI | | -0.27 | -0.06 | 0.03 | 0.04 | -0.20 | -0.05 | 0.05 | 0.01 |
|  | Upper CI | | 0.33 | 0.08 | 0.16 | 0.17 | 0.57 | 0.13 | 0.24 | 0.19 |
|  | *p* value | | 0.83 | 0.81 | **<0.01** | **<0.01** | **<0.01** | 0.35 | 0.42 | **<0.01** |
| **Fat (per 1 g.Kg^-1^.d^-1^)** | | |  |  |  |  |  |  |  |  |
| Intravenous | | *B* coefficient | 0.23 | -0.03 | -0.11 | -0.15 | 0.17 | -0.05 | -0.16 | -0.13 |
|  | | Lower CI | -0.03 | -0.13 | -0.2 | -0.25 | -0.17 | -0.18 | -0.28 | -0.27 |
|  | | Upper CI | 0.48 | 0.07 | -0.03 | -0.06 | 0.50 | 0.08 | -0.04 | 0.00 |
|  | | *p* value | 0.08 | 0.61 | **0.01** | **<0.01** | 0.32 | 0.43 | **0.01** | **0.05** |
| Enteral | | *B* coefficient | 0.03 | 0.02 | 0.06 | 0.07 | 0.12 | 0.04 | 0.09 | 0.07 |
|  | | Lower CI | -0.18 | -0.03 | 0.02 | 0.03 | -0.16 | -0.03 | 0.03 | 0.01 |
|  | | Upper CI | 0.24 | 0.07 | 0.1 | 0.11 | 0.39 | 0.11 | 0.14 | 0.12 |
|  | | *p* value | 0.77 | 0.5 | **<0.01** | **<0.001** | 0.40 | 0.24 | **<0.01** | **0.01** |
| **Carbohydrate (per 1 g.Kg^-1^.d^-1^)** | | |  |  |  |  |  |  |  |  |
| Intravenous | | *B* coefficient | 0.02 | 0 | -0.03 | -0.03 | 0.00 | -0.01 | -0.04 | -0.03 |
|  | | Lower CI | -0.04 | -0.03 | -0.05 | -0.05 | -0.08 | -0.04 | -0.06 | -0.05 |
|  | | Upper CI | 0.07 | 0.02 | -0.01 | -0.01 | 0.07 | 0.02 | -0.01 | 0.00 |
|  | | *p* value | 0.6 | 0.7 | **<0.01** | **<0.001** | 0.94 | 0.54 | **<0.01** | **0.04** |
| Enteral | | *B* coefficient | 0.01 | 0 | 0.03 | 0.03 | 0.06 | 0.01 | 0.04 | 0.03 |
|  | | Lower CI | -0.08 | -0.02 | 0.01 | 0.01 | -0.07 | -0.02 | 0.02 | 0.01 |
|  | | Upper CI | 0.11 | 0.03 | 0.05 | 0.05 | 0.18 | 0.04 | 0.07 | 0.05 |
|  | | p value | 0.8 | 0.7 | **<0.01** | **<0.001** | 0.36 | 0.36 | **<0.001** | **0.01** |
| **Energy:protein ratio  (per Kcal.g^-1^ protein)** | | |  |  |  |  |  |  |  |  |
| Intravenous | | *B* coefficient | -0.01 | -0.01 | -0.01 | -0.01 | -0.01 | -0.01 | -0.01 | -0.03 |
|  | | Lower CI | -0.02 | -0.02 | -0.02 | -0.03 | -0.02 | -0.02 | -0.02 | -0.06 |
|  | | Upper CI | 0 | 0 | 0 | 0.02 | 0.01 | 0.00 | 0.00 | 0.00 |
|  | | *p* value | 0.08 | **0.05** | **0.01** | 0.55 | 0.29 | 0.07 | **0.01** | **0.04** |
| Enteral | | *B* coefficient | 0 | 0.01 | -0.01 | -0.01 | -0.06 | 0.01 | -0.02 | -0.02 |
|  | | Lower CI | -0.06 | -0.01 | -0.03 | -0.03 | -0.16 | -0.02 | -0.04 | -0.03 |
|  | | Upper CI | 0.06 | 0.04 | 0 | 0 | 0.03 | 0.04 | 0.00 | 0.00 |
|  | | *p* value | 0.99 | 0.21 | 0.07 | 0.08 | 0.20 | 0.40 | **0.02** | 0.07 |

**Head Circumference**

| **Predictor** | | **Birth to 4 weeks** | | | | | **Birth to 36 weeks’ CA** | | | |
| --- | --- | --- | --- | --- | --- | --- | --- | --- | --- | --- |
| **Fluid (per 100 mL.Kg^-1^.d^-1^)** | | **Week 1** | **Week 2** | **Week 3** | **Week 4** | **Week 1** | | **Week 2** | **Week 3** | **Week 4** |
| Total | *B* coefficient | -0.29 | -0.30 | -0.03 | -0.25 | -0.19 | | 0.64 | 0.55 | 0.59 |
|  | Lower CI | -0.88 | -0.92 | -0.52 | -0.72 | -0.96 | | -0.12 | -0.03 | 0.01 |
|  | Upper CI | 0.31 | 0.31 | 0.46 | 0.21 | 0.58 | | 1.41 | 1.14 | 1.18 |
|  | *p* value | 0.35 | 0.33 | 0.92 | 0.29 | 0.63 | | 0.10 | 0.06 | **0.05** |
| Intravenous | *B* coefficient | -0.33 | -0.11 | -0.10 | 0.08 | -0.06 | | -0.15 | -0.32 | -0.25 |
|  | Lower CI | -0.77 | -0.28 | -0.26 | -0.08 | -0.60 | | -0.36 | -0.51 | -0.46 |
|  | Upper CI | 0.11 | 0.05 | 0.05 | 0.24 | 0.49 | | 0.06 | -0.13 | -0.05 |
|  | *p* value | 0.15 | 0.19 | 0.20 | 0.35 | 0.84 | | 0.17 | **<0.001** | **0.02** |
| Enteral | *B* coefficient | 0.32 | 0.08 | 0.08 | -0.08 | -0.07 | | 0.17 | 0.31 | 0.24 |
|  | Lower CI | -0.29 | -0.08 | -0.06 | -0.22 | -0.81 | | -0.02 | 0.14 | 0.06 |
|  | Upper CI | 0.92 | 0.24 | 0.22 | 0.06 | 0.66 | | 0.37 | 0.48 | 0.41 |
|  | p value | 0.30 | 0.32 | 0.26 | 0.27 | 0.85 | | 0.08 | **<0.001** | **0.01** |
| Breastmilk | *B* coefficient | 0.31 | 0.05 | 0.00 | -0.13 | -0.05 | | 0.14 | 0.22 | 0.10 |
|  | Lower CI | -0.29 | -0.11 | -0.13 | -0.26 | -0.79 | | -0.05 | 0.06 | -0.06 |
|  | Upper CI | 0.91 | 0.21 | 0.13 | -0.01 | 0.68 | | 0.34 | 0.38 | 0.26 |
|  | *p* value | 0.31 | 0.52 | 0.97 | **0.03** | 0.89 | | 0.16 | **0.01** | 0.22 |

| **Energy (per 10 Kcal. Kg^-1^.d^-1^)** | | |  |  |  |  |  |  |  |  |
| --- | --- | --- | --- | --- | --- | --- | --- | --- | --- | --- |
| Total | *B* coefficient | | 0.04 | 0.05 | 0.04 | -0.01 | 0.06 | 0.09 | 0.09 | 0.06 |
|  | Lower CI | | -0.06 | 0.00 | 0.00 | -0.04 | -0.06 | 0.03 | 0.05 | 0.02 |
|  | Upper CI | | 0.13 | 0.09 | 0.07 | 0.02 | 0.17 | 0.14 | 0.13 | 0.10 |
|  | *p* value | | 0.47 | **0.04** | **0.03** | 0.56 | 0.34 | **<0.01** | **<.0001** | **<0.01** |
| Intravenous | *B* coefficient | | -0.03 | -0.01 | -0.01 | 0.02 | 0.06 | -0.02 | -0.05 | -0.04 |
|  | Lower CI | | -0.12 | -0.04 | -0.04 | -0.01 | -0.05 | -0.05 | -0.08 | -0.08 |
|  | Upper CI | | 0.07 | 0.02 | 0.02 | 0.05 | 0.18 | 0.02 | -0.02 | 0.00 |
|  | *p* value | | 0.59 | 0.59 | 0.56 | 0.15 | 0.28 | 0.44 | **<0.01** | **0.03** |
| Enteral | *B* coefficient | | 0.05 | 0.01 | 0.01 | -0.01 | -0.01 | 0.02 | 0.04 | 0.03 |
|  | Lower CI | | -0.04 | -0.01 | -0.01 | -0.03 | -0.11 | 0.00 | 0.02 | 0.01 |
|  | Upper CI | | 0.13 | 0.03 | 0.03 | 0.01 | 0.10 | 0.05 | 0.06 | 0.05 |
|  | *p* value | | 0.28 | 0.19 | 0.17 | 0.26 | 0.91 | 0.06 | **<0.001** | **<0.01** |
| **Protein (per 1 g.Kg^-1^.d^-1^)** | | |  |  |  |  |  |  |  |  |
| Total | *B* coefficient | | -0.02 | 0.13 | 0.17 | 0.09 | 0.05 | 0.26 | 0.16 | 0.25 |
|  | Lower CI | | -0.17 | -0.02 | 0.05 | -0.03 | -0.13 | 0.07 | 0.01 | 0.10 |
|  | Upper CI | | 0.13 | 0.29 | 0.29 | 0.21 | 0.23 | 0.46 | 0.30 | 0.40 |
|  | *p* value | | 0.82 | 0.10 | **0.01** | 0.15 | 0.59 | **0.01** | **0.04** | **<0.001** |
| Intravenous | *B* coefficient | | -0.05 | -0.03 | -0.02 | 0.06 | 0.04 | -0.04 | -0.14 | -0.09 |
|  | Lower CI | | -0.18 | -0.10 | -0.09 | -0.01 | -0.12 | -0.13 | -0.22 | -0.19 |
|  | Upper CI | | 0.08 | 0.04 | 0.05 | 0.13 | 0.20 | 0.05 | -0.05 | 0.00 |
|  | *p* value | | 0.46 | 0.40 | 0.52 | 0.10 | 0.63 | 0.36 | **<0.01** | **0.05** |
| Enteral | *B* coefficient | | 0.15 | 0.05 | 0.06 | -0.02 | 0.00 | 0.07 | 0.16 | 0.13 |
|  | Lower CI | | -0.12 | -0.02 | 0.00 | -0.08 | -0.33 | -0.01 | 0.08 | 0.05 |
|  | Upper CI | | 0.42 | 0.11 | 0.13 | 0.04 | 0.33 | 0.15 | 0.23 | 0.20 |
|  | *p* value | | 0.28 | 0.16 | **0.04** | 0.51 | 0.99 | 0.07 | **<0.0001** | **<0.001** |
| **Fat (per 1 g.Kg^-1^.d^-1^)** | | |  |  |  |  |  |  |  |  |
| Intravenous | | *B* coefficient | 0.02 | 0.01 | 0.01 | 0.07 | 0.10 | -0.01 | -0.11 | -0.13 |
|  | | Lower CI | -0.21 | -0.08 | -0.08 | -0.02 | -0.18 | -0.12 | -0.21 | -0.24 |
|  | | Upper CI | 0.26 | 0.10 | 0.09 | 0.16 | 0.38 | 0.10 | -0.01 | -0.02 |
|  | | *p* value | 0.85 | 0.88 | 0.89 | 0.11 | 0.48 | 0.85 | **0.03** | **0.03** |
| Enteral | | *B* coefficient | 0.10 | 0.03 | 0.02 | -0.02 | -0.03 | 0.05 | 0.08 | 0.06 |
|  | | Lower CI | -0.09 | -0.02 | -0.01 | -0.05 | -0.25 | 0.00 | 0.04 | 0.02 |
|  | | Upper CI | 0.29 | 0.07 | 0.06 | 0.02 | 0.20 | 0.11 | 0.13 | 0.11 |
|  | | *p* value | 0.29 | 0.26 | 0.23 | 0.29 | 0.83 | 0.07 | **<0.001** | **0.01** |
| **Carbohydrate (per 1 g.Kg^-1^.d^-1^)** | | |  |  |  |  |  |  |  |  |
| Intravenous | | *B* coefficient | -0.02 | -0.01 | -0.01 | 0.01 | 0.04 | -0.01 | -0.03 | -0.03 |
|  | | Lower CI | -0.07 | -0.03 | -0.03 | -0.01 | -0.03 | -0.04 | -0.06 | -0.05 |
|  | | Upper CI | 0.03 | 0.01 | 0.01 | 0.03 | 0.10 | 0.01 | -0.01 | 0.00 |
|  | | *p* value | 0.45 | 0.33 | 0.32 | 0.24 | 0.24 | 0.26 | **<0.01** | **0.03** |
| Enteral | | *B* coefficient | 0.05 | 0.02 | 0.01 | -0.01 | 0.00 | 0.02 | 0.04 | 0.03 |
|  | | Lower CI | -0.04 | 0.00 | 0.00 | -0.02 | -0.11 | 0.00 | 0.02 | 0.01 |
|  | | Upper CI | 0.13 | 0.03 | 0.03 | 0.01 | 0.10 | 0.05 | 0.06 | 0.05 |
|  | | *p* value | 0.26 | 0.12 | 0.09 | 0.33 | 0.96 | 0.06 | **<0.001** | **<0.01** |
| **Energy:protein ratio  (per Kcal.g^-1^ protein)** | | |  |  |  |  |  |  |  |  |
| Intravenous | | *B* coefficient | 0.00 | 0.00 | 0.00 | -0.01 | 0.00 | 0.00 | 0.00 | -0.02 |
|  | | Lower CI | -0.01 | -0.01 | 0.00 | -0.03 | -0.01 | -0.01 | 0.00 | -0.05 |
|  | | Upper CI | 0.01 | 0.01 | 0.01 | 0.01 | 0.01 | 0.01 | 0.01 | 0.00 |
|  | | *p* value | 0.73 | 0.60 | 0.61 | 0.42 | 0.91 | 0.60 | 0.42 | 0.09 |
| Enteral | | *B* coefficient | 0.01 | -0.01 | -0.02 | -0.01 | -0.04 | -0.01 | -0.03 | -0.03 |
|  | | Lower CI | -0.05 | -0.03 | -0.03 | -0.02 | -0.12 | -0.04 | -0.05 | -0.04 |
|  | | Upper CI | 0.07 | 0.01 | -0.01 | 0.01 | 0.05 | 0.01 | -0.01 | -0.01 |
|  | | *p* value | 0.74 | 0.24 | **<0.01** | 0.40 | 0.40 | 0.29 | **<0.001** | **<0.001** |

#### Data are linear regression analyses adjusted for sex, site, gestational age at birth, birthweight z-score. Significant correlations are shown in bold.
