# Supplementary material for: Relationships between Neonatal Nutrition and Growth to 36 Weeks’ Corrected Age in ELBW Babies–Secondary Cohort Analysis from the Provide Trial
Source: Nutrients. 2020 Mar 13;12(3):760. doi: 10.3390/nu12030760 (PMC7146349; doi:10.3390/nu12030760)
Supplement: Supplementary file 1 [file nutrients-12-00760-s001.zip › Nutrients Growth Supplementary Table 2.docx]

**Supplementary table 2A** Relationship between selected nutrient intakes in each of the first 4 weeks and expected growth (weight, length and head circumference), and target growth

| **Weight expected growth** | | | **Birth to 4 weeks** | | | | **Birth to 36 weeks corrected age** | | | |
| --- | --- | --- | --- | --- | --- | --- | --- | --- | --- | --- |
| **Predictor** | **Quintile** | **Value** | **Week 1** | **Week 2** | **Week 3** | **Week 4** | **Week 1** | **Week 2** | **Week 3** | **Week 4** |
| **Fluid** | | |  |  |  |  |  |  |  |  |
| **Total** | 1 vs 3 | Odds ratio | 1.09 | 2.17 | 0.78 | 0.95 | 1.09 | 0.92 | 0.51 | 0.79 |
|  |  | Lower CI | 0.37 | 0.78 | 0.29 | 0.37 | 0.42 | 0.40 | 0.23 | 0.37 |
|  |  | Upper CI | 3.22 | 6.03 | 2.10 | 2.47 | 2.81 | 2.13 | 1.14 | 1.69 |
|  |  | p value | 0.88 | 0.14 | 0.62 | 0.92 | 0.86 | 0.84 | 0.10 | 0.54 |
|  | 2 vs 3 | Odds ratio | 1.09 | 0.93 | 0.63 | 0.46 | 1.67 | 0.94 | 0.75 | 1.57 |
|  |  | Lower CI | 0.38 | 0.35 | 0.24 | 0.17 | 0.72 | 0.41 | 0.34 | 0.71 |
|  |  | Upper CI | 3.09 | 2.47 | 1.69 | 1.27 | 3.87 | 2.15 | 1.70 | 3.49 |
|  |  | p value | 0.87 | 0.88 | 0.36 | 0.13 | 0.23 | 0.88 | 0.49 | 0.27 |
|  | 4 vs 3 | Odds ratio | 0.90 | 0.88 | 1.24 | 0.43 | 0.95 | 1.69 | 0.90 | 0.92 |
|  |  | Lower CI | 0.37 | 0.35 | 0.45 | 0.16 | 0.46 | 0.77 | 0.39 | 0.41 |
|  |  | Upper CI | 2.21 | 2.22 | 3.37 | 1.18 | 1.99 | 3.68 | 2.05 | 2.05 |
|  |  | p value | 0.82 | 0.78 | 0.68 | 0.10 | 0.90 | 0.19 | 0.80 | 0.84 |
|  | 5 vs 3 | Odds ratio | 0.96 | 1.41 | 0.80 | 0.62 | 0.67 | 1.49 | 1.38 | 2.98 |
|  |  | Lower CI | 0.37 | 0.52 | 0.31 | 0.23 | 0.29 | 0.65 | 0.62 | 1.25 |
|  |  | Upper CI | 2.47 | 3.79 | 2.03 | 1.69 | 1.51 | 3.41 | 3.10 | 7.10 |
|  |  | p value | 0.93 | 0.50 | 0.63 | 0.35 | 0.33 | 0.34 | 0.43 | **0.01** |
| **IV** | 1 vs 3 | Odds ratio | 1.39 | 1.14 | 3.70 | - | 0.93 | 0.74 | 1.34 | - |
|  |  | Lower CI | 0.48 | 0.43 | 1.44 | - | 0.40 | 0.33 | 0.61 | - |
|  |  | Upper CI | 4.06 | 3.02 | 9.50 | - | 2.16 | 1.66 | 2.97 | - |
|  |  | p value | 0.55 | 0.79 | 0.01 | - | 0.86 | 0.47 | 0.47 | - |
|  | 2 vs 3 | Odds ratio | 1.16 | 0.79 | 6.10 | 1.09 | 1.48 | 1.45 | 0.84 | 1.04 |
|  |  | Lower CI | 0.44 | 0.31 | 1.78 | 0.44 | 0.65 | 0.64 | 0.33 | 0.46 |
|  |  | Upper CI | 3.07 | 1.98 | 20.83 | 2.66 | 3.37 | 3.26 | 2.13 | 2.35 |
|  |  | p value | 0.77 | 0.61 | **<0.01** | 0.86 | 0.35 | 0.37 | 0.71 | 0.93 |
|  | 4 vs 3 | Odds ratio | 1.48 | 1.17 | 1.40 | 1.09 | 0.91 | 0.83 | 0.40 | 0.63 |
|  |  | Lower CI | 0.58 | 0.45 | 0.56 | 0.42 | 0.42 | 0.37 | 0.18 | 0.26 |
|  |  | Upper CI | 3.82 | 3.09 | 3.53 | 2.88 | 2.00 | 1.90 | 0.88 | 1.51 |
|  |  | p value | 0.42 | 0.75 | 0.47 | 0.86 | 0.82 | 0.66 | **0.02** | 0.30 |
|  | 5 vs 3 | Odds ratio | 0.76 | 1.14 | 4.16 | 1.48 | 0.59 | 0.47 | 0.33 | 0.25 |
|  |  | Lower CI | 0.27 | 0.43 | 1.58 | 0.56 | 0.25 | 0.20 | 0.14 | 0.10 |
|  |  | Upper CI | 2.08 | 3.05 | 10.95 | 3.92 | 1.38 | 1.11 | 0.77 | 0.61 |
|  |  | p value | 0.59 | 0.80 | **<0.01** | 0.43 | 0.22 | 0.08 | **0.01** | **<0.01** |
| **Enteral** | 1 vs 3 | Odds ratio | 0.91 | 1.42 | 1.93 | 1.76 | 1.12 | 0.42 | 0.24 | 0.28 |
|  |  | Lower CI | 0.34 | 0.52 | 0.70 | 0.66 | 0.49 | 0.18 | 0.10 | 0.12 |
|  |  | Upper CI | 2.43 | 3.90 | 5.32 | 4.72 | 2.56 | 0.96 | 0.57 | 0.66 |
|  |  | p value | 0.85 | 0.50 | 0.20 | 0.26 | 0.80 | **0.04** | **<0.01** | **<0.01** |
|  | 2 vs 3 | Odds ratio | 0.71 | 0.41 | 1.14 | 1.67 | 0.87 | 1.08 | 0.32 | 0.61 |
|  |  | Lower CI | 0.27 | 0.16 | 0.44 | 0.62 | 0.39 | 0.48 | 0.14 | 0.27 |
|  |  | Upper CI | 1.82 | 1.04 | 2.94 | 4.50 | 1.91 | 2.43 | 0.72 | 1.36 |
|  |  | p value | 0.47 | 0.06 | 0.79 | 0.31 | 0.72 | 0.85 | **0.01** | 0.22 |
|  | 4 vs 3 | Odds ratio | 0.99 | 0.42 | 1.17 | 1.18 | 0.76 | 0.94 | 0.67 | 0.76 |
|  |  | Lower CI | 0.37 | 0.16 | 0.45 | 0.46 | 0.35 | 0.43 | 0.30 | 0.34 |
|  |  | Upper CI | 2.62 | 1.10 | 3.03 | 3.03 | 1.69 | 2.06 | 1.50 | 1.69 |
|  |  | p value | 0.98 | 0.08 | 0.75 | 0.73 | 0.51 | 0.87 | 0.32 | 0.50 |
|  | 5 vs 3 | Odds ratio | 0.89 | 0.68 | 2.22 | 1.63 | 0.85 | 0.95 | 0.81 | 1.30 |
|  |  | Lower CI | 0.34 | 0.25 | 0.79 | 0.56 | 0.39 | 0.42 | 0.34 | 0.53 |
|  |  | Upper CI | 2.35 | 1.88 | 6.25 | 4.75 | 1.87 | 2.13 | 1.94 | 3.20 |
|  |  | p value | 0.82 | 0.45 | 0.13 | 0.37 | 0.69 | 0.89 | 0.64 | 0.57 |
| **Breastmilk** | 1 vs 3 | Odds ratio | 0.99 | 1.93 | 2.54 | 2.08 | 1.15 | 0.50 | 0.46 | 0.58 |
|  |  | Lower CI | 0.37 | 0.71 | 0.95 | 0.79 | 0.50 | 0.22 | 0.20 | 0.26 |
|  |  | Upper CI | 2.65 | 5.30 | 6.83 | 5.46 | 2.64 | 1.13 | 1.04 | 1.30 |
|  |  | p value | 0.99 | 0.20 | 0.06 | 0.14 | 0.74 | 0.10 | 0.06 | 0.19 |
|  | 2 vs 3 | Odds ratio | 0.76 | 0.56 | 1.09 | 1.31 | 0.94 | 0.98 | 0.77 | 0.78 |
|  |  | Lower CI | 0.29 | 0.22 | 0.43 | 0.52 | 0.43 | 0.44 | 0.36 | 0.36 |
|  |  | Upper CI | 1.98 | 1.40 | 2.75 | 3.34 | 2.07 | 2.17 | 1.66 | 1.69 |
|  |  | p value | 0.58 | 0.21 | 0.85 | 0.57 | 0.88 | 0.96 | 0.50 | 0.53 |
|  | 4 vs 3 | Odds ratio | 1.13 | 0.65 | 1.44 | 1.91 | 0.81 | 0.88 | 1.53 | 1.34 |
|  |  | Lower CI | 0.43 | 0.25 | 0.54 | 0.71 | 0.37 | 0.40 | 0.67 | 0.60 |
|  |  | Upper CI | 3.00 | 1.65 | 3.84 | 5.15 | 1.79 | 1.93 | 3.49 | 2.99 |
|  |  | p value | 0.80 | 0.36 | 0.47 | 0.20 | 0.60 | 0.76 | 0.31 | 0.47 |
|  | 5 vs 3 | Odds ratio | 0.97 | 0.65 | 1.51 | 1.28 | 0.89 | 0.80 | 1.00 | 1.00 |
|  |  | Lower CI | 0.37 | 0.24 | 0.58 | 0.48 | 0.40 | 0.36 | 0.45 | 0.44 |
|  |  | Upper CI | 2.54 | 1.78 | 3.95 | 3.41 | 1.96 | 1.81 | 2.24 | 2.23 |
|  |  | p value | 0.95 | 0.40 | 0.40 | 0.63 | 0.77 | 0.60 | 1.00 | 0.99 |

| **Weight expected growth** | | |  | | | |  | | | |
| --- | --- | --- | --- | --- | --- | --- | --- | --- | --- | --- |
| **Energy** | | |  |  |  |  |  |  |  |  |
| **Total** | 1 vs 3 | Odds ratio | 0.63 | 1.92 | 0.93 | 2.28 | 1.06 | 0.53 | 0.26 | 0.23 |
|  |  | Lower CI | 0.22 | 0.77 | 0.35 | 0.86 | 0.40 | 0.24 | 0.12 | 0.10 |
|  |  | Upper CI | 1.85 | 4.79 | 2.51 | 6.06 | 2.84 | 1.17 | 0.59 | 0.53 |
|  |  | p value | 0.40 | 0.16 | 0.89 | 0.10 | 0.90 | 0.12 | **<0.01** | **<0.01** |
|  | 2 vs 3 | Odds ratio | 2.61 | 0.82 | 1.01 | 4.18 | 0.94 | 0.48 | 0.49 | 0.42 |
|  |  | Lower CI | 0.96 | 0.31 | 0.41 | 1.54 | 0.41 | 0.21 | 0.23 | 0.19 |
|  |  | Upper CI | 7.05 | 2.14 | 2.51 | 11.38 | 2.12 | 1.09 | 1.06 | 0.93 |
|  |  | p value | 0.06 | 0.68 | 0.99 | **0.01** | 0.87 | 0.08 | 0.07 | **0.03** |
|  | 4 vs 3 | Odds ratio | 2.10 | 1.24 | 1.41 | 2.37 | 0.60 | 1.44 | 1.37 | 1.16 |
|  |  | Lower CI | 0.79 | 0.49 | 0.52 | 0.90 | 0.27 | 0.63 | 0.58 | 0.49 |
|  |  | Upper CI | 5.57 | 3.15 | 3.86 | 6.26 | 1.31 | 3.29 | 3.22 | 2.73 |
|  |  | p value | 0.14 | 0.65 | 0.50 | 0.08 | 0.20 | 0.39 | 0.47 | 0.74 |
|  | 5 vs 3 | Odds ratio | 2.12 | 1.58 | 1.12 | 2.93 | 0.62 | 0.88 | 0.81 | 0.94 |
|  |  | Lower CI | 0.74 | 0.58 | 0.39 | 0.98 | 0.27 | 0.37 | 0.34 | 0.38 |
|  |  | Upper CI | 6.11 | 4.30 | 3.21 | 8.71 | 1.43 | 2.07 | 1.94 | 2.32 |
|  |  | p value | 0.16 | 0.37 | 0.84 | 0.05 | 0.26 | 0.76 | 0.64 | 0.89 |
| **IV** | 1 vs 3 | Odds ratio | 0.74 | 0.68 | 2.96 | - | 0.75 | 1.13 | 1.45 | - |
|  |  | Lower CI | 0.24 | 0.25 | 1.22 | - | 0.30 | 0.51 | 0.69 | - |
|  |  | Upper CI | 2.27 | 1.83 | 7.22 | - | 1.87 | 2.51 | 3.04 | - |
|  |  | p value | 0.60 | 0.44 | **0.02** | - | 0.54 | 0.77 | 0.33 | - |
|  | 2 vs 3 | Odds ratio | 0.92 | 0.35 | 10.06 | 0.97 | 1.22 | 1.35 | 1.34 | 0.76 |
|  |  | Lower CI | 0.34 | 0.13 | 1.42 | 0.35 | 0.53 | 0.62 | 0.39 | 0.28 |
|  |  | Upper CI | 2.50 | 0.93 | 71.06 | 2.70 | 2.78 | 2.94 | 4.66 | 2.03 |
|  |  | p value | 0.87 | **0.03** | **0.02** | 0.96 | 0.64 | 0.44 | 0.64 | 0.58 |
|  | 4 vs 3 | Odds ratio | 0.94 | 0.37 | 1.44 | 1.07 | 0.91 | 0.82 | 0.62 | 0.56 |
|  |  | Lower CI | 0.35 | 0.14 | 0.58 | 0.36 | 0.38 | 0.37 | 0.28 | 0.20 |
|  |  | Upper CI | 2.57 | 0.97 | 3.58 | 3.21 | 2.16 | 1.81 | 1.33 | 1.58 |
|  |  | p value | 0.90 | **0.04** | 0.43 | 0.90 | 0.83 | 0.62 | 0.22 | 0.27 |
|  | 5 vs 3 | Odds ratio | 1.27 | 0.82 | 3.56 | 1.53 | 0.65 | 0.91 | 0.39 | 0.18 |
|  |  | Lower CI | 0.44 | 0.29 | 1.37 | 0.51 | 0.26 | 0.39 | 0.18 | 0.06 |
|  |  | Upper CI | 3.72 | 2.31 | 9.24 | 4.57 | 1.60 | 2.10 | 0.88 | 0.53 |
|  |  | p value | 0.66 | 0.71 | **0.01** | 0.45 | 0.35 | 0.82 | **0.02** | **<0.01** |
| **Enteral** | 1 vs 3 | Odds ratio | 1.03 | 1.64 | 1.36 | 1.69 | 1.09 | 0.50 | 0.33 | 0.21 |
|  |  | Lower CI | 0.39 | 0.60 | 0.50 | 0.67 | 0.48 | 0.22 | 0.14 | 0.09 |
|  |  | Upper CI | 2.72 | 4.51 | 3.69 | 4.29 | 2.51 | 1.14 | 0.77 | 0.47 |
|  |  | p value | 0.96 | 0.34 | 0.54 | 0.27 | 0.83 | 0.10 | **0.01** | **<0.001** |
|  | 2 vs 3 | Odds ratio | 0.79 | 0.42 | 0.65 | 1.30 | 0.85 | 1.41 | 0.51 | 0.41 |
|  |  | Lower CI | 0.31 | 0.16 | 0.26 | 0.51 | 0.38 | 0.63 | 0.24 | 0.19 |
|  |  | Upper CI | 2.04 | 1.10 | 1.62 | 3.27 | 1.87 | 3.17 | 1.11 | 0.90 |
|  |  | p value | 0.63 | 0.08 | 0.35 | 0.58 | 0.68 | 0.41 | 0.09 | **0.03** |
|  | 4 vs 3 | Odds ratio | 1.30 | 0.57 | 2.02 | 1.44 | 0.74 | 1.59 | 2.33 | 0.93 |
|  |  | Lower CI | 0.49 | 0.22 | 0.72 | 0.57 | 0.34 | 0.72 | 0.97 | 0.40 |
|  |  | Upper CI | 3.46 | 1.50 | 5.68 | 3.66 | 1.64 | 3.53 | 5.59 | 2.15 |
|  |  | p value | 0.59 | 0.25 | 0.18 | 0.44 | 0.46 | 0.25 | 0.06 | 0.87 |
|  | 5 vs 3 | Odds ratio | 0.97 | 0.82 | 0.96 | 1.83 | 0.82 | 1.05 | 1.07 | 0.83 |
|  |  | Lower CI | 0.37 | 0.30 | 0.34 | 0.65 | 0.37 | 0.46 | 0.45 | 0.34 |
|  |  | Upper CI | 2.54 | 2.27 | 2.77 | 5.15 | 1.80 | 2.38 | 2.55 | 2.04 |
|  |  | p value | 0.95 | 0.70 | 0.95 | 0.25 | 0.61 | 0.91 | 0.88 | 0.68 |
| **Weight expected growth** | | |  | | | |  | | | |
| **Protein** | | |  |  |  |  |  |  |  |  |
| **Total** | 1 vs 3 | Odds ratio | 0.65 | 0.74 | 0.20 | 0.79 | 1.29 | 0.58 | 0.62 | 0.36 |
|  |  | Lower CI | 0.25 | 0.25 | 0.07 | 0.27 | 0.58 | 0.24 | 0.27 | 0.14 |
|  |  | Upper CI | 1.69 | 2.18 | 0.61 | 2.31 | 2.91 | 1.39 | 1.45 | 0.89 |
|  |  | p value | 0.37 | 0.58 | **<0.01** | 0.67 | 0.53 | 0.22 | 0.27 | **0.03** |
|  | 2 vs 3 | Odds ratio | 0.76 | 0.59 | 0.56 | 1.82 | 1.78 | 0.51 | 1.29 | 0.36 |
|  |  | Lower CI | 0.29 | 0.21 | 0.20 | 0.69 | 0.80 | 0.23 | 0.59 | 0.16 |
|  |  | Upper CI | 1.96 | 1.68 | 1.56 | 4.81 | 3.95 | 1.16 | 2.85 | 0.82 |
|  |  | p value | 0.56 | 0.33 | 0.26 | 0.22 | 0.16 | 0.11 | 0.52 | **0.02** |
|  | 4 vs 3 | Odds ratio | 1.47 | 0.70 | 0.87 | 1.11 | 1.71 | 2.02 | 2.13 | 1.44 |
|  |  | Lower CI | 0.55 | 0.24 | 0.31 | 0.43 | 0.78 | 0.81 | 0.92 | 0.62 |
|  |  | Upper CI | 3.93 | 2.04 | 2.41 | 2.89 | 3.71 | 5.03 | 4.92 | 3.35 |
|  |  | p value | 0.44 | 0.52 | 0.78 | 0.83 | 0.18 | 0.13 | 0.08 | 0.40 |
|  | 5 vs 3 | Odds ratio | 1.27 | 0.67 | 0.99 | 1.55 | 1.99 | 1.24 | 1.41 | 1.27 |
|  |  | Lower CI | 0.44 | 0.21 | 0.34 | 0.57 | 0.86 | 0.48 | 0.60 | 0.52 |
|  |  | Upper CI | 3.66 | 2.15 | 2.91 | 4.24 | 4.64 | 3.22 | 3.28 | 3.10 |
|  |  | p value | 0.66 | 0.50 | 0.99 | 0.39 | 0.11 | 0.66 | 0.43 | 0.60 |

| **IV** | 1 vs 3 | Odds ratio | 0.57 | 1.03 | - | - | 0.76 | 0.81 | - | - |
| --- | --- | --- | --- | --- | --- | --- | --- | --- | --- | --- |
|  |  | Lower CI | 0.21 | 0.38 | - | - | 0.34 | 0.35 | - | - |
|  |  | Upper CI | 1.58 | 2.83 | - | - | 1.70 | 1.88 | - | - |
|  |  | p value | 0.28 | 0.95 | - | - | 0.50 | 0.63 | - | - |
|  | 2 vs 3 | Odds ratio | 0.36 | 0.56 | - | - | 0.68 | 0.57 | - | - |
|  |  | Lower CI | 0.14 | 0.22 | - | - | 0.31 | 0.26 | - | - |
|  |  | Upper CI | 0.97 | 1.43 | - | - | 1.51 | 1.26 | - | - |
|  |  | p value | **0.04** | 0.23 | - | - | 0.34 | 0.17 | - | - |
|  | 4 vs 3 | Odds ratio | 1.41 | 0.75 | - | - | 0.60 | 0.64 | - | - |
|  |  | Lower CI | 0.52 | 0.30 | - | - | 0.27 | 0.29 | - | - |
|  |  | Upper CI | 3.84 | 1.88 | - | - | 1.31 | 1.42 | - | - |
|  |  | p value | 0.50 | 0.54 | - | - | 0.20 | 0.27 | - | - |
|  | 5 vs 3 | Odds ratio | 0.72 | 0.99 | - | - | 0.93 | 0.43 | - | - |
|  |  | Lower CI | 0.25 | 0.38 | - | - | 0.39 | 0.19 | - | - |
|  |  | Upper CI | 2.10 | 2.62 | - | - | 2.21 | 0.99 | - | - |
|  |  | p value | 0.54 | 0.99 | - | - | 0.86 | **0.05** | - | - |
| **Enteral** | 1 vs 3 | Odds ratio | 0.93 | 2.43 | 1.18 | 1.20 | 1.23 | 0.55 | 0.25 | 0.20 |
|  |  | Lower CI | 0.35 | 0.88 | 0.42 | 0.46 | 0.54 | 0.24 | 0.11 | 0.08 |
|  |  | Upper CI | 2.48 | 6.70 | 3.28 | 3.15 | 2.81 | 1.23 | 0.59 | 0.46 |
|  |  | p value | 0.88 | 0.09 | 0.75 | 0.71 | 0.63 | 0.15 | **<0.01** | **<0.001** |
|  | 2 vs 3 | Odds ratio | 0.71 | 0.74 | 0.40 | 0.85 | 0.96 | 1.55 | 0.59 | 0.53 |
|  |  | Lower CI | 0.28 | 0.30 | 0.16 | 0.33 | 0.44 | 0.70 | 0.28 | 0.23 |
|  |  | Upper CI | 1.85 | 1.85 | 1.02 | 2.20 | 2.12 | 3.42 | 1.26 | 1.19 |
|  |  | p value | 0.49 | 0.52 | 0.05 | 0.74 | 0.93 | 0.28 | 0.17 | 0.12 |
|  | 4 vs 3 | Odds ratio | 1.11 | 1.12 | 0.95 | 0.73 | 0.84 | 1.70 | 2.49 | 1.34 |
|  |  | Lower CI | 0.42 | 0.42 | 0.34 | 0.28 | 0.38 | 0.76 | 1.02 | 0.58 |
|  |  | Upper CI | 2.94 | 2.98 | 2.67 | 1.90 | 1.85 | 3.82 | 6.09 | 3.13 |
|  |  | p value | 0.84 | 0.82 | 0.92 | 0.52 | 0.67 | 0.20 | **0.05** | 0.50 |
|  | 5 vs 3 | Odds ratio | 0.83 | 1.20 | 1.26 | 1.34 | 1.05 | 1.63 | 1.21 | 1.16 |
|  |  | Lower CI | 0.31 | 0.45 | 0.43 | 0.48 | 0.47 | 0.71 | 0.51 | 0.48 |
|  |  | Upper CI | 2.17 | 3.22 | 3.69 | 3.73 | 2.31 | 3.72 | 2.87 | 2.82 |
|  |  | p value | 0.70 | 0.72 | 0.68 | 0.57 | 0.91 | 0.25 | 0.67 | 0.74 |
| **Weight expected growth** | | |  | | | |  | | | |
| **Fat** | | |  |  |  |  |  |  |  |  |
| **IV** | 1 vs 3 | Odds ratio | 0.56 | 0.80 | - | - | 0.81 | 1.47 | - | - |
|  |  | Lower CI | 0.18 | 0.30 | - | - | 0.34 | 0.66 | - | - |
|  |  | Upper CI | 1.78 | 2.16 | - | - | 1.97 | 3.28 | - | - |
|  |  | p value | 0.32 | 0.66 | - | - | 0.65 | 0.35 | - | - |
|  | 2 vs 3 | Odds ratio | 0.87 | 0.47 | - | - | 1.88 | 1.26 | - | - |
|  |  | Lower CI | 0.26 | 0.18 | - | - | 0.74 | 0.59 | - | - |
|  |  | Upper CI | 2.87 | 1.22 | - | - | 4.74 | 2.68 | - | - |
|  |  | p value | 0.81 | 0.12 | - | - | 0.18 | 0.55 | - | - |
|  | 4 vs 3 | Odds ratio | 1.01 | 1.59 | - | - | 1.00 | 1.58 | - | - |
|  |  | Lower CI | 0.39 | 0.57 | - | - | 0.45 | 0.68 | - | - |
|  |  | Upper CI | 2.62 | 4.45 | - | - | 2.24 | 3.65 | - | - |
|  |  | p value | 0.99 | 0.38 | - | - | 1.00 | 0.29 | - | - |
|  | 5 vs 3 | Odds ratio | 1.77 | 0.45 | - | - | 0.91 | 1.07 | - | - |
|  |  | Lower CI | 0.60 | 0.17 | - | - | 0.37 | 0.47 | - | - |
|  |  | Upper CI | 5.26 | 1.24 | - | - | 2.24 | 2.44 | - | - |
|  |  | p value | 0.30 | 0.12 | - | - | 0.84 | 0.88 | - | - |
| **Enteral** | 1 vs 3 | Odds ratio | - | 1.51 | 1.94 | 1.58 | - | 0.36 | 0.20 | 0.30 |
|  |  | Lower CI | - | 0.55 | 0.70 | 0.59 | - | 0.16 | 0.08 | 0.13 |
|  |  | Upper CI | - | 4.18 | 5.43 | 4.28 | - | 0.83 | 0.50 | 0.69 |
|  |  | p value | - | 0.42 | 0.21 | 0.37 | - | **0.02** | **<0.01** | **<0.01** |
|  | 2 vs 3 | Odds ratio | - | 0.39 | 1.21 | 1.37 | - | 0.96 | 0.25 | 0.60 |
|  |  | Lower CI | - | 0.15 | 0.46 | 0.51 | - | 0.43 | 0.11 | 0.27 |
|  |  | Upper CI | - | 1.01 | 3.18 | 3.71 | - | 2.16 | 0.58 | 1.35 |
|  |  | p value | - | 0.05 | 0.69 | 0.54 | - | 0.92 | **<0.01** | 0.22 |
|  | 4 vs 3 | Odds ratio | - | 0.53 | 1.06 | 0.91 | - | 1.03 | 0.52 | 0.77 |
|  |  | Lower CI | - | 0.20 | 0.41 | 0.34 | - | 0.46 | 0.23 | 0.35 |
|  |  | Upper CI | - | 1.38 | 2.73 | 2.42 | - | 2.28 | 1.17 | 1.73 |
|  |  | p value | - | 0.19 | 0.90 | 0.86 | - | 0.95 | 0.11 | 0.53 |
|  | 5 vs 3 | Odds ratio | - | 0.68 | 2.25 | 1.91 | - | 0.73 | 0.83 | 1.69 |
|  |  | Lower CI | - | 0.25 | 0.79 | 0.64 | - | 0.32 | 0.33 | 0.68 |
|  |  | Upper CI | - | 1.87 | 6.39 | 5.66 | - | 1.64 | 2.04 | 4.24 |
|  |  | p value | - | 0.46 | 0.13 | 0.25 | - | 0.44 | 0.68 | 0.26 |

| **Weight expected growth** | | |  | | | |  | | | |
| --- | --- | --- | --- | --- | --- | --- | --- | --- | --- | --- |
| **Carbohydrate** | | |  |  |  |  |  |  |  |  |
| **IV** | 1 vs 3 | Odds ratio | 0.70 | 0.68 | - | - | 0.62 | 0.88 | - | - |
|  |  | Lower CI | 0.25 | 0.24 | - | - | 0.26 | 0.39 | - | - |
|  |  | Upper CI | 1.96 | 1.89 | - | - | 1.49 | 1.98 | - | - |
|  |  | p value | 0.50 | 0.46 | - | - | 0.29 | 0.75 | - | - |
|  | 2 vs 3 | Odds ratio | 1.02 | 0.56 | - | - | 0.84 | 1.45 | - | - |
|  |  | Lower CI | 0.37 | 0.21 | - | - | 0.36 | 0.65 | - | - |
|  |  | Upper CI | 2.80 | 1.46 | - | - | 1.96 | 3.25 | - | - |
|  |  | p value | 0.97 | 0.23 | - | - | 0.69 | 0.37 | - | - |
|  | 4 vs 3 | Odds ratio | 0.76 | 0.32 | - | - | 0.48 | 0.79 | - | - |
|  |  | Lower CI | 0.26 | 0.12 | - | - | 0.19 | 0.35 | - | - |
|  |  | Upper CI | 2.20 | 0.86 | - | - | 1.21 | 1.81 | - | - |
|  |  | p value | 0.61 | **0.02** | - | - | 0.12 | 0.58 | - | - |
|  | 5 vs 3 | Odds ratio | 0.60 | 0.99 | - | - | 0.55 | 0.72 | - | - |
|  |  | Lower CI | 0.20 | 0.35 | - | - | 0.21 | 0.30 | - | - |
|  |  | Upper CI | 1.85 | 2.83 | - | - | 1.41 | 1.72 | - | - |
|  |  | p value | 0.38 | 0.99 | - | - | 0.21 | 0.46 | - | - |
| **Enteral** | 1 vs 3 | Odds ratio | - | 1.72 | 1.26 | 1.67 | - | 0.43 | 0.26 | 0.17 |
|  |  | Lower CI | - | 0.63 | 0.45 | 0.63 | - | 0.19 | 0.11 | 0.07 |
|  |  | Upper CI | - | 4.70 | 3.50 | 4.40 | - | 0.96 | 0.60 | 0.40 |
|  |  | p value | - | 0.29 | 0.66 | 0.30 | - | **0.04** | **<0.01** | **<0.0001** |
|  | 2 vs 3 | Odds ratio | - | 0.46 | 0.56 | 0.98 | - | 1.20 | 0.50 | 0.39 |
|  |  | Lower CI | - | 0.18 | 0.22 | 0.38 | - | 0.54 | 0.23 | 0.17 |
|  |  | Upper CI | - | 1.17 | 1.40 | 2.52 | - | 2.66 | 1.09 | 0.90 |
|  |  | p value | - | 0.10 | 0.21 | 0.96 | - | 0.65 | 0.08 | **0.03** |
|  | 4 vs 3 | Odds ratio | - | 0.73 | 1.15 | 1.45 | - | 1.51 | 1.97 | 0.98 |
|  |  | Lower CI | - | 0.27 | 0.41 | 0.56 | - | 0.68 | 0.80 | 0.41 |
|  |  | Upper CI | - | 1.94 | 3.19 | 3.78 | - | 3.37 | 4.82 | 2.31 |
|  |  | p value | - | 0.52 | 0.79 | 0.45 | - | 0.31 | 0.14 | 0.96 |
|  | 5 vs 3 | Odds ratio | - | 0.76 | 1.09 | 1.81 | - | 1.27 | 1.14 | 0.72 |
|  |  | Lower CI | - | 0.29 | 0.37 | 0.63 | - | 0.55 | 0.47 | 0.29 |
|  |  | Upper CI | - | 2.04 | 3.23 | 5.23 | - | 2.89 | 2.79 | 1.80 |
|  |  | p value | - | 0.59 | 0.88 | 0.27 | - | 0.58 | 0.78 | 0.48 |
| **Weight expected growth** | | |  | | | |  | | | |
| **Energy:protein ratio** | | |  |  |  |  |  |  |  |  |
| **Total** | 1 vs 3 | Odds ratio | 1.93 | 0.63 | 1.97 | 1.04 | 3.17 | 0.51 | 0.44 | 0.73 |
|  |  | Lower CI | 0.64 | 0.22 | 0.70 | 0.35 | 1.26 | 0.22 | 0.19 | 0.30 |
|  |  | Upper CI | 5.86 | 1.81 | 5.54 | 3.11 | 7.98 | 1.21 | 1.00 | 1.77 |
|  |  | p value | 0.24 | 0.39 | 0.20 | 0.95 | **0.01** | 0.13 | 0.05 | 0.49 |
|  | 2 vs 3 | Odds ratio | 1.21 | 0.78 | 1.42 | 0.85 | 2.24 | 0.81 | 0.44 | 1.05 |
|  |  | Lower CI | 0.43 | 0.29 | 0.45 | 0.26 | 0.97 | 0.36 | 0.18 | 0.41 |
|  |  | Upper CI | 3.43 | 2.11 | 4.47 | 2.72 | 5.18 | 1.80 | 1.08 | 2.71 |
|  |  | p value | 0.72 | 0.62 | 0.55 | 0.78 | 0.06 | 0.61 | 0.07 | 0.91 |
|  | 4 vs 3 | Odds ratio | 1.71 | 0.49 | 1.42 | 0.88 | 1.33 | 0.95 | 1.04 | 1.90 |
|  |  | Lower CI | 0.66 | 0.19 | 0.53 | 0.30 | 0.62 | 0.43 | 0.45 | 0.76 |
|  |  | Upper CI | 4.41 | 1.24 | 3.82 | 2.61 | 2.89 | 2.08 | 2.37 | 4.74 |
|  |  | p value | 0.27 | 0.13 | 0.49 | 0.82 | 0.47 | 0.89 | 0.93 | 0.17 |
|  | 5 vs 3 | Odds ratio | 0.87 | 0.46 | 0.28 | 0.33 | 1.24 | 0.86 | 0.50 | 0.64 |
|  |  | Lower CI | 0.33 | 0.18 | 0.10 | 0.10 | 0.56 | 0.38 | 0.21 | 0.25 |
|  |  | Upper CI | 2.27 | 1.21 | 0.82 | 1.05 | 2.75 | 1.96 | 1.20 | 1.60 |
|  |  | p value | 0.77 | 0.11 | **0.02** | 0.06 | 0.60 | 0.72 | 0.12 | 0.34 |
| **IV** | 1 vs 3 | Odds ratio | 1.85 | 0.08 | - | - | 2.50 | 1.20 | - | - |
|  |  | Lower CI | 0.57 | 0.02 | - | - | 0.97 | 0.45 | - | - |
|  |  | Upper CI | 6.03 | 0.32 | - | - | 6.48 | 3.20 | - | - |
|  |  | p value | 0.31 | **<0.001** | - | - | 0.06 | 0.72 | - | - |
|  | 2 vs 3 | Odds ratio | 1.01 | 0.29 | - | - | 2.25 | 0.98 | - | - |
|  |  | Lower CI | 0.36 | 0.09 | - | - | 0.98 | 0.40 | - | - |
|  |  | Upper CI | 2.82 | 0.96 | - | - | 5.15 | 2.37 | - | - |
|  |  | p value | 0.98 | **0.04** | - | - | 0.05 | 0.96 | - | - |
|  | 4 vs 3 | Odds ratio | 1.36 | 0.81 | - | - | 1.56 | 1.06 | - | - |
|  |  | Lower CI | 0.50 | 0.26 | - | - | 0.69 | 0.46 | - | - |
|  |  | Upper CI | 3.68 | 2.53 | - | - | 3.53 | 2.45 | - | - |
|  |  | p value | 0.55 | 0.72 | - | - | 0.29 | 0.90 | - | - |
|  | 5 vs 3 | Odds ratio | 0.76 | 0.17 | - | - | 1.26 | 1.06 | - | - |
|  |  | Lower CI | 0.27 | 0.05 | - | - | 0.55 | 0.46 | - | - |
|  |  | Upper CI | 2.14 | 0.51 | - | - | 2.92 | 2.46 | - | - |
|  |  | p value | 0.60 | **<0.01** | - | - | 0.59 | 0.89 | - | - |

| **Enteral** | 1 vs 3 | Odds ratio | - | 1.22 | 0.67 | 1.16 | - | 1.48 | 2.37 | 0.62 |
| --- | --- | --- | --- | --- | --- | --- | --- | --- | --- | --- |
|  |  | Lower CI | - | 0.38 | 0.23 | 0.39 | - | 0.58 | 0.94 | 0.21 |
|  |  | Upper CI | - | 3.95 | 1.95 | 3.53 | - | 3.79 | 6.00 | 1.87 |
|  |  | p value | - | 0.74 | 0.46 | 0.79 | - | 0.41 | 0.07 | 0.40 |
|  | 2 vs 3 | Odds ratio | - | 2.47 | 1.00 | 1.13 | - | 2.28 | 2.42 | 0.41 |
|  |  | Lower CI | - | 0.83 | 0.34 | 0.41 | - | 0.92 | 1.00 | 0.16 |
|  |  | Upper CI | - | 7.34 | 2.95 | 3.10 | - | 5.65 | 5.86 | 1.05 |
|  |  | p value | - | 0.10 | 1.00 | 0.81 | - | 0.07 | 0.05 | 0.06 |
|  | 4 vs 3 | Odds ratio | - | 0.89 | 0.44 | 0.78 | - | 0.80 | 0.61 | 0.14 |
|  |  | Lower CI | - | 0.33 | 0.16 | 0.29 | - | 0.35 | 0.27 | 0.05 |
|  |  | Upper CI | - | 2.40 | 1.22 | 2.10 | - | 1.83 | 1.41 | 0.38 |
|  |  | p value | - | 0.81 | 0.11 | 0.62 | - | 0.60 | 0.25 | **<0.0001** |
|  | 5 vs 3 | Odds ratio | - | 1.95 | 0.28 | 0.72 | - | 1.01 | 0.47 | 0.11 |
|  |  | Lower CI | - | 0.71 | 0.08 | 0.24 | - | 0.45 | 0.18 | 0.04 |
|  |  | Upper CI | - | 5.34 | 0.96 | 2.14 | - | 2.27 | 1.24 | 0.32 |
|  |  | p value | - | 0.19 | **0.04** | 0.56 | - | 0.98 | 0.13 | **<0.0001** |
| **Length expected growth** | | | **Birth to 4 weeks** | | | | **Birth to 36 weeks corrected age** | | | |
| **Predictor** | **Quintile** | **Value** | **Week 1** | **Week 2** | **Week 3** | **Week 4** | **Week 1** | **Week 2** | **Week 3** | **Week 4** |
| **Fluid** | | |  |  |  |  |  |  |  |  |
| **Total** | 1 vs 3 | Odds ratio | 1.58 | 1.67 | 0.95 | 0.71 | 1.51 | 1.49 | 0.53 | 0.72 |
|  |  | Lower CI | 0.68 | 0.79 | 0.46 | 0.34 | 0.60 | 0.65 | 0.25 | 0.33 |
|  |  | Upper CI | 3.67 | 3.52 | 1.95 | 1.49 | 3.82 | 3.44 | 1.15 | 1.55 |
|  |  | p value | 0.29 | 0.18 | 0.88 | 0.37 | 0.38 | 0.35 | 0.11 | 0.40 |
|  | 2 vs 3 | Odds ratio | 2.08 | 1.14 | 0.79 | 1.19 | 1.49 | 0.76 | 0.40 | 0.69 |
|  |  | Lower CI | 0.99 | 0.55 | 0.39 | 0.58 | 0.69 | 0.34 | 0.19 | 0.32 |
|  |  | Upper CI | 4.36 | 2.36 | 1.60 | 2.41 | 3.25 | 1.73 | 0.86 | 1.46 |
|  |  | p value | 0.05 | 0.73 | 0.51 | 0.64 | 0.31 | 0.52 | **0.02** | 0.33 |
|  | 4 vs 3 | Odds ratio | 1.51 | 1.28 | 1.12 | 1.88 | 1.49 | 0.97 | 0.50 | 1.34 |
|  |  | Lower CI | 0.77 | 0.62 | 0.54 | 0.90 | 0.72 | 0.45 | 0.22 | 0.62 |
|  |  | Upper CI | 2.96 | 2.62 | 2.32 | 3.89 | 3.09 | 2.08 | 1.11 | 2.90 |
|  |  | p value | 0.23 | 0.51 | 0.76 | 0.09 | 0.28 | 0.93 | 0.09 | 0.46 |
|  | 5 vs 3 | Odds ratio | 1.51 | 0.85 | 1.42 | 1.54 | 0.88 | 0.89 | 0.67 | 1.15 |
|  |  | Lower CI | 0.69 | 0.41 | 0.70 | 0.74 | 0.35 | 0.39 | 0.31 | 0.52 |
|  |  | Upper CI | 3.32 | 1.77 | 2.89 | 3.20 | 2.23 | 1.99 | 1.44 | 2.54 |
|  |  | p value | 0.30 | 0.66 | 0.33 | 0.25 | 0.78 | 0.77 | 0.31 | 0.73 |
| **IV** | 1 vs 3 | Odds ratio | 0.58 | 0.89 | 1.93 | - | 0.70 | 0.98 | 1.04 | - |
|  |  | Lower CI | 0.27 | 0.42 | 0.98 | - | 0.31 | 0.44 | 0.52 | - |
|  |  | Upper CI | 1.26 | 1.87 | 3.82 | - | 1.58 | 2.14 | 2.09 | - |
|  |  | p value | 0.17 | 0.75 | 0.06 | - | 0.40 | 0.95 | 0.92 | - |
|  | 2 vs 3 | Odds ratio | 0.76 | 1.17 | 0.86 | 1.75 | 0.68 | 1.33 | 0.73 | 0.90 |
|  |  | Lower CI | 0.37 | 0.58 | 0.36 | 0.85 | 0.32 | 0.63 | 0.30 | 0.41 |
|  |  | Upper CI | 1.56 | 2.35 | 2.03 | 3.60 | 1.44 | 2.78 | 1.79 | 1.95 |
|  |  | p value | 0.46 | 0.66 | 0.73 | 0.13 | 0.32 | 0.46 | 0.49 | 0.78 |
|  | 4 vs 3 | Odds ratio | 0.59 | 1.21 | 0.91 | 1.04 | 0.58 | 1.27 | 0.65 | 0.71 |
|  |  | Lower CI | 0.29 | 0.58 | 0.45 | 0.47 | 0.27 | 0.57 | 0.30 | 0.30 |
|  |  | Upper CI | 1.19 | 2.54 | 1.84 | 2.29 | 1.25 | 2.85 | 1.40 | 1.69 |
|  |  | p value | 0.14 | 0.61 | 0.78 | 0.92 | 0.16 | 0.56 | 0.27 | 0.44 |
|  | 5 vs 3 | Odds ratio | 0.62 | 0.70 | 0.77 | 0.62 | 0.53 | 0.88 | 0.46 | 0.49 |
|  |  | Lower CI | 0.28 | 0.33 | 0.36 | 0.28 | 0.22 | 0.37 | 0.19 | 0.19 |
|  |  | Upper CI | 1.37 | 1.49 | 1.64 | 1.40 | 1.27 | 2.07 | 1.10 | 1.21 |
|  |  | p value | 0.24 | 0.36 |  | 0.25 | 0.15 | 0.76 | 0.08 | 0.12 |
| **Enteral** | 1 vs 3 | Odds ratio | 0.95 | 1.08 | 0.49 | 0.53 | 0.90 | 0.80 | 0.73 | 0.98 |
|  |  | Lower CI | 0.45 | 0.52 | 0.22 | 0.25 | 0.40 | 0.35 | 0.30 | 0.41 |
|  |  | Upper CI | 1.98 | 2.24 | 1.09 | 1.12 | 2.04 | 1.83 | 1.77 | 2.34 |
|  |  | p value | 0.88 | 0.84 | 0.08 | 0.09 | 0.81 | 0.60 | 0.48 | 0.97 |
|  | 2 vs 3 | Odds ratio | 1.28 | 0.83 | 0.56 | 0.80 | 1.10 | 0.97 | 0.83 | 1.82 |
|  |  | Lower CI | 0.63 | 0.41 | 0.27 | 0.39 | 0.52 | 0.45 | 0.38 | 0.82 |
|  |  | Upper CI | 2.58 | 1.67 | 1.14 | 1.64 | 2.35 | 2.10 | 1.82 | 4.06 |
|  |  | p value | 0.49 | 0.60 | 0.11 | 0.53 | 0.80 | 0.94 | 0.65 | 0.14 |
|  | 4 vs 3 | Odds ratio | 1.21 | 0.95 | 0.98 | 1.41 | 1.35 | 0.82 | 2.44 | 2.62 |
|  |  | Lower CI | 0.59 | 0.47 | 0.49 | 0.69 | 0.63 | 0.39 | 1.16 | 1.22 |
|  |  | Upper CI | 2.47 | 1.91 | 1.99 | 2.85 | 2.93 | 1.71 | 5.11 | 5.61 |
|  |  | p value | 0.61 | 0.89 | 0.96 | 0.35 | 0.44 | 0.59 | **0.02** | **0.01** |
|  | 5 vs 3 | Odds ratio | 1.03 | 0.95 | 0.81 | 1.44 | 0.92 | 1.08 | 0.99 | 2.18 |
|  |  | Lower CI | 0.51 | 0.46 | 0.38 | 0.67 | 0.42 | 0.50 | 0.45 | 0.96 |
|  |  | Upper CI | 2.10 | 1.97 | 1.76 | 3.11 | 1.99 | 2.33 | 2.21 | 4.95 |
|  |  | p value | 0.94 | 0.89 | 0.60 | 0.35 | 0.83 | 0.85 | 0.99 | 0.06 |

| **Breastmilk** | 1 vs 3 | Odds ratio | 0.93 | 1.09 | 0.62 | 0.50 | 0.94 | 0.78 | 0.86 | 0.92 |
| --- | --- | --- | --- | --- | --- | --- | --- | --- | --- | --- |
|  |  | Lower CI | 0.45 | 0.53 | 0.29 | 0.24 | 0.42 | 0.35 | 0.36 | 0.40 |
|  |  | Upper CI | 1.95 | 2.24 | 1.34 | 1.06 | 2.13 | 1.75 | 2.05 | 2.13 |
|  |  | p value | 0.85 | 0.82 | 0.22 | 0.07 | 0.88 | 0.54 | 0.73 | 0.84 |
|  | 2 vs 3 | Odds ratio | 1.14 | 0.60 | 0.88 | 0.88 | 1.06 | 0.94 | 1.15 | 1.26 |
|  |  | Lower CI | 0.57 | 0.30 | 0.43 | 0.44 | 0.50 | 0.43 | 0.53 | 0.57 |
|  |  | Upper CI | 2.30 | 1.22 | 1.78 | 1.79 | 2.26 | 2.01 | 2.49 | 2.77 |
|  |  | p value | 0.71 | 0.16 | 0.72 | 0.73 | 0.88 | 0.86 | 0.73 | 0.57 |
|  | 4 vs 3 | Odds ratio | 1.05 | 0.72 | 1.73 | 1.48 | 1.34 | 0.80 | 3.11 | 2.17 |
|  |  | Lower CI | 0.51 | 0.35 | 0.84 | 0.74 | 0.62 | 0.38 | 1.46 | 1.03 |
|  |  | Upper CI | 2.14 | 1.45 | 3.54 | 2.97 | 2.91 | 1.67 | 6.64 | 4.54 |
|  |  | p value | 0.90 | 0.35 | 0.14 | 0.27 | 0.46 | 0.55 | **<0.01** | **0.04** |
|  | 5 vs 3 | Odds ratio | 0.96 | 0.91 | 0.99 | 1.41 | 0.91 | 0.90 | 1.04 | 1.56 |
|  |  | Lower CI | 0.47 | 0.44 | 0.47 | 0.66 | 0.42 | 0.42 | 0.46 | 0.70 |
|  |  | Upper CI | 1.95 | 1.90 | 2.07 | 2.99 | 1.98 | 1.93 | 2.33 | 3.47 |
|  |  | p value | 0.91 | 0.81 | 0.98 | 0.38 | 0.81 | 0.78 | 0.93 | 0.28 |
| **Length expected growth** | | |  | | | |  | | | |
| **Energy** | | |  |  |  |  |  |  |  |  |
| **Total** | 1 vs 3 | Odds ratio | 0.74 | 0.96 | 0.51 | 0.38 | 0.73 | 0.66 | 0.97 | 0.78 |
|  |  | Lower CI | 0.33 | 0.47 | 0.24 | 0.18 | 0.29 | 0.30 | 0.43 | 0.34 |
|  |  | Upper CI | 1.70 | 1.94 | 1.09 | 0.81 | 1.81 | 1.46 | 2.17 | 1.75 |
|  |  | p value | 0.48 | 0.90 | 0.08 | **0.01** | 0.49 | 0.30 | 0.94 | 0.54 |
|  | 2 vs 3 | Odds ratio | 0.63 | 0.94 | 0.58 | 1.18 | 0.70 | 0.91 | 0.87 | 1.54 |
|  |  | Lower CI | 0.31 | 0.47 | 0.29 | 0.58 | 0.32 | 0.43 | 0.40 | 0.71 |
|  |  | Upper CI | 1.26 | 1.87 | 1.14 | 2.41 | 1.51 | 1.93 | 1.87 | 3.35 |
|  |  | p value | 0.19 | 0.86 | 0.11 | 0.64 | 0.36 | 0.80 | 0.72 | 0.28 |
|  | 4 vs 3 | Odds ratio | 0.60 | 0.85 | 0.81 | 0.92 | 0.63 | 0.48 | 1.67 | 1.84 |
|  |  | Lower CI | 0.30 | 0.42 | 0.40 | 0.45 | 0.30 | 0.22 | 0.78 | 0.86 |
|  |  | Upper CI | 1.20 | 1.73 | 1.66 | 1.87 | 1.32 | 1.06 | 3.57 | 3.93 |
|  |  | p value | 0.15 | 0.65 | 0.56 | 0.81 | 0.22 | 0.07 | 0.18 | 0.12 |
|  | 5 vs 3 | Odds ratio | 0.89 | 0.94 | 0.97 | 1.26 | 0.52 | 1.11 | 1.79 | 1.37 |
|  |  | Lower CI | 0.43 | 0.44 | 0.45 | 0.58 | 0.23 | 0.50 | 0.81 | 0.60 |
|  |  | Upper CI | 1.87 | 1.98 | 2.08 | 2.74 | 1.18 | 2.47 | 3.93 | 3.13 |
|  |  | p value | 0.76 | 0.86 | 0.93 | 0.56 | 0.12 | 0.81 | 0.15 | 0.45 |
| **IV** | 1 vs 3 | Odds ratio | 0.53 | 0.69 | 1.86 | 0.84 | 0.94 | 1.13 | 1.46 | 0.78 |
|  |  | Lower CI | 0.23 | 0.33 | 0.95 | 0.40 | 0.40 | 0.52 | 0.73 | 0.34 |
|  |  | Upper CI | 1.23 | 1.45 | 3.62 | 1.76 | 2.24 | 2.47 | 2.94 | 1.75 |
|  |  | p value | 0.14 | 0.33 | 0.07 | 0.65 | 0.89 | 0.75 | 0.28 | 0.54 |
|  | 2 vs 3 | Odds ratio | 0.81 | 1.41 | 0.74 | 1.25 | 0.49 | 1.10 | 1.02 | 0.97 |
|  |  | Lower CI | 0.40 | 0.70 | 0.24 | 0.53 | 0.22 | 0.53 | 0.33 | 0.37 |
|  |  | Upper CI | 1.65 | 2.87 | 2.22 | 2.96 | 1.06 | 2.28 | 3.15 | 2.56 |
|  |  | p value | 0.57 | 0.34 | 0.59 | 0.61 | 0.07 | 0.81 | 0.97 | 0.95 |
|  | 4 vs 3 | Odds ratio | 0.70 | 0.99 | 1.05 | 0.60 | 0.87 | 1.10 | 1.11 | 0.67 |
|  |  | Lower CI | 0.34 | 0.49 | 0.52 | 0.23 | 0.39 | 0.51 | 0.51 | 0.23 |
|  |  | Upper CI | 1.44 | 2.01 | 2.15 | 1.52 | 1.97 | 2.40 | 2.41 | 1.94 |
|  |  | p value | 0.33 | 0.98 | 0.88 | 0.28 | 0.74 | 0.81 | 0.79 | 0.46 |
|  | 5 vs 3 | Odds ratio | 0.63 | 0.87 | 0.92 | 0.57 | 0.67 | 0.84 | 0.68 | 0.61 |
|  |  | Lower CI | 0.29 | 0.42 | 0.44 | 0.22 | 0.28 | 0.37 | 0.29 | 0.21 |
|  |  | Upper CI | 1.38 | 1.82 | 1.93 | 1.44 | 1.59 | 1.94 | 1.60 | 1.79 |
|  |  | p value | 0.25 | 0.72 | 0.82 | 0.23 | 0.36 | 0.69 | 0.38 | 0.37 |
| **Enteral** | 1 vs 3 | Odds ratio | 0.91 | 0.84 | 0.46 | 0.50 | 0.85 | 0.96 | 0.71 | 0.67 |
|  |  | Lower CI | 0.43 | 0.40 | 0.21 | 0.24 | 0.38 | 0.41 | 0.30 | 0.30 |
|  |  | Upper CI | 1.89 | 1.76 | 1.01 | 1.03 | 1.91 | 2.23 | 1.68 | 1.52 |
|  |  | p value | 0.79 | 0.65 | 0.05 | 0.06 | 0.69 | 0.92 | 0.44 | 0.34 |
|  | 2 vs 3 | Odds ratio | 1.23 | 0.70 | 0.63 | 0.58 | 1.04 | 1.43 | 0.85 | 0.89 |
|  |  | Lower CI | 0.61 | 0.35 | 0.31 | 0.28 | 0.49 | 0.65 | 0.39 | 0.41 |
|  |  | Upper CI | 2.48 | 1.40 | 1.29 | 1.18 | 2.21 | 3.13 | 1.85 | 1.91 |
|  |  | p value | 0.57 | 0.31 | 0.21 | 0.13 | 0.92 | 0.37 | 0.68 | 0.76 |
|  | 4 vs 3 | Odds ratio | 1.12 | 0.82 | 1.13 | 1.31 | 1.29 | 1.34 | 1.75 | 1.40 |
|  |  | Lower CI | 0.55 | 0.40 | 0.55 | 0.65 | 0.60 | 0.64 | 0.82 | 0.67 |
|  |  | Upper CI | 2.31 | 1.66 | 2.31 | 2.65 | 2.80 | 2.81 | 3.71 | 2.92 |
|  |  | p value | 0.75 | 0.58 | 0.75 | 0.45 | 0.51 | 0.44 | 0.15 | 0.38 |
|  | 5 vs 3 | Odds ratio | 0.97 | 0.61 | 0.87 | 1.30 | 0.79 | 1.41 | 1.62 | 1.34 |
|  |  | Lower CI | 0.47 | 0.29 | 0.40 | 0.61 | 0.36 | 0.63 | 0.73 | 0.60 |
|  |  | Upper CI | 1.97 | 1.29 | 1.91 | 2.78 | 1.72 | 3.14 | 3.62 | 3.02 |
|  |  | p value | 0.93 | 0.19 | 0.73 | 0.50 | 0.55 | 0.41 | 0.24 | 0.48 |

| **Length expected growth** | | |  | | | | | | |  | | | | | | | |
| --- | --- | --- | --- | --- | --- | --- | --- | --- | --- | --- | --- | --- | --- | --- | --- | --- | --- |
| **Protein** | | |  |  | |  | |  | |  | |  | |  | |  | |
| **Total** | 1 vs 3 | Odds ratio | 0.75 | | 0.81 | | 1.44 | | 0.64 | | 0.83 | | 1.14 | | 1.90 | | 0.53 |
|  |  | Lower CI | 0.35 | | 0.36 | | 0.64 | | 0.27 | | 0.36 | | 0.47 | | 0.79 | | 0.21 |
|  |  | Upper CI | 1.62 | | 1.81 | | 3.24 | | 1.49 | | 1.90 | | 2.77 | | 4.57 | | 1.33 |
|  |  | p value | 0.46 | | 0.60 | | 0.38 | | 0.30 | | 0.66 | | 0.78 | | 0.15 | | 0.18 |
|  | 2 vs 3 | Odds ratio | 0.75 | | 0.64 | | 1.58 | | 0.79 | | 0.80 | | 0.72 | | 0.98 | | 0.66 |
|  |  | Lower CI | 0.36 | | 0.29 | | 0.74 | | 0.38 | | 0.36 | | 0.31 | | 0.42 | | 0.29 |
|  |  | Upper CI | 1.55 | | 1.37 | | 3.37 | | 1.67 | | 1.77 | | 1.66 | | 2.31 | | 1.48 |
|  |  | p value | 0.44 | | 0.25 | | 0.23 | | 0.54 | | 0.58 | | 0.44 | | 0.97 | | 0.31 |
|  | 4 vs 3 | Odds ratio | 0.86 | | 0.51 | | 2.94 | | 1.18 | | 1.00 | | 1.29 | | 2.95 | | 0.63 |
|  |  | Lower CI | 0.42 | | 0.24 | | 1.38 | | 0.57 | | 0.48 | | 0.59 | | 1.32 | | 0.29 |
|  |  | Upper CI | 1.74 | | 1.07 | | 6.25 | | 2.43 | | 2.08 | | 2.82 | | 6.61 | | 1.35 |
|  |  | p value | 0.67 | | 0.07 | | **0.01** | | 0.65 | | 0.99 | | 0.52 | | **0.01** | | 0.23 |
|  | 5 vs 3 | Odds ratio | 1.06 | | 0.77 | | 1.93 | | 0.98 | | 0.95 | | 1.28 | | 2.11 | | 0.97 |
|  |  | Lower CI | 0.51 | | 0.33 | | 0.89 | | 0.47 | | 0.43 | | 0.53 | | 0.92 | | 0.44 |
|  |  | Upper CI | 2.23 | | 1.75 | | 4.18 | | 2.07 | | 2.07 | | 3.07 | | 4.84 | | 2.16 |
|  |  | p value | 0.88 | | 0.53 | | 0.10 | | 0.96 | | 0.89 | | 0.58 | | 0.08 | | 0.94 |
| **IV** | 1 vs 3 | Odds ratio | 1.06 | | 0.65 | | - | | - | | 1.22 | | 0.88 | | - | | - |
|  |  | Lower CI | 0.49 | | 0.31 | | - | | - | | 0.55 | | 0.40 | | - | | - |
|  |  | Upper CI | 2.30 | | 1.36 | | - | | - | | 2.71 | | 1.91 | | - | | - |
|  |  | p value | 0.88 | | 0.25 | | - | | - | | 0.63 | | 0.74 | | - | | - |
|  | 2 vs 3 | Odds ratio | 0.56 | | 0.57 | | - | | - | | 0.61 | | 0.86 | | - | | - |
|  |  | Lower CI | 0.27 | | 0.27 | | - | | - | | 0.28 | | 0.40 | | - | | - |
|  |  | Upper CI | 1.18 | | 1.17 | | - | | - | | 1.37 | | 1.82 | | - | | - |
|  |  | p value | 0.13 | | 0.12 | | - | | - | | 0.23 | | 0.69 | | - | | - |
|  | 4 vs 3 | Odds ratio | 0.89 | | 0.56 | | - | | - | | 0.99 | | 0.77 | | - | | - |
|  |  | Lower CI | 0.44 | | 0.27 | | - | | - | | 0.47 | | 0.36 | | - | | - |
|  |  | Upper CI | 1.81 | | 1.13 | | - | | - | | 2.08 | | 1.68 | | - | | - |
|  |  | p value | 0.75 | | 0.11 | | - | | - | | 0.98 | | 0.52 | | - | | - |
|  | 5 vs 3 | Odds ratio | 1.13 | | 0.68 | | - | | - | | 1.16 | | 0.85 | | - | | - |
|  |  | Lower CI | 0.53 | | 0.33 | | - | | - | | 0.53 | | 0.39 | | - | | - |
|  |  | Upper CI | 2.40 | | 1.38 | | - | | - | | 2.55 | | 1.85 | | - | | - |
|  |  | p value | 0.75 | | 0.28 | | - | | - | | 0.71 | | 0.67 | | - | | - |
| **Enteral** | 1 vs 3 | Odds ratio | 0.90 | | 0.84 | | 0.48 | | 0.45 | | 0.91 | | 0.86 | | 0.84 | | 0.53 |
|  |  | Lower CI | 0.43 | | 0.41 | | 0.22 | | 0.21 | | 0.40 | | 0.38 | | 0.36 | | 0.22 |
|  |  | Upper CI | 1.88 | | 1.74 | | 1.04 | | 0.96 | | 2.05 | | 1.93 | | 1.99 | | 1.25 |
|  |  | p value | 0.78 | | 0.64 | | 0.06 | | **0.04** | | 0.82 | | 0.71 | | 0.70 | | 0.15 |
|  | 2 vs 3 | Odds ratio | 1.15 | | 0.71 | | 0.77 | | 0.53 | | 1.13 | | 1.25 | | 1.02 | | 0.83 |
|  |  | Lower CI | 0.57 | | 0.35 | | 0.38 | | 0.24 | | 0.53 | | 0.58 | | 0.47 | | 0.37 |
|  |  | Upper CI | 2.31 | | 1.43 | | 1.54 | | 1.13 | | 2.41 | | 2.69 | | 2.18 | | 1.88 |
|  |  | p value | 0.70 | | 0.34 | | 0.46 | | 0.10 | | 0.75 | | 0.57 | | 0.97 | | 0.66 |
|  | 4 vs 3 | Odds ratio | 1.30 | | 0.64 | | 0.89 | | 1.17 | | 1.38 | | 1.04 | | 1.46 | | 1.26 |
|  |  | Lower CI | 0.64 | | 0.32 | | 0.43 | | 0.57 | | 0.64 | | 0.49 | | 0.68 | | 0.60 |
|  |  | Upper CI | 2.66 | | 1.31 | | 1.84 | | 2.41 | | 2.98 | | 2.20 | | 3.10 | | 2.65 |
|  |  | p value | 0.47 | | 0.23 | | 0.75 | | 0.67 | | 0.41 | | 0.92 | | 0.33 | | 0.55 |
|  | 5 vs 3 | Odds ratio | 0.87 | | 0.73 | | 0.93 | | 0.83 | | 0.89 | | 1.08 | | 1.80 | | 1.18 |
|  |  | Lower CI | 0.43 | | 0.35 | | 0.43 | | 0.38 | | 0.41 | | 0.49 | | 0.81 | | 0.53 |
|  |  | Upper CI | 1.79 | | 1.55 | | 2.00 | | 1.78 | | 1.94 | | 2.39 | | 4.03 | | 2.65 |
|  |  | p value | 0.71 | | 0.42 | | 0.86 | | 0.63 | | 0.77 | | 0.85 | | 0.15 | | 0.68 |
| **Length expected growth** | | |  | | | | | | | |  | | | | | | |
| **Fat** | | |  | |  | |  | |  | |  | |  | |  | |  |
| **IV** | 1 vs 3 | Odds ratio | 2.02 | | 0.69 | | - | | - | | 1.02 | | 0.91 | | - | | - |
|  |  | Lower CI | 0.88 | | 0.33 | | - | | - | | 0.43 | | 0.42 | | - | | - |
|  |  | Upper CI | 4.64 | | 1.44 | | - | | - | | 2.39 | | 2.02 | | - | | - |
|  |  | p value | 0.10 | | 0.32 | | - | | - | | 0.97 | | 0.82 | | - | | - |
|  | 2 vs 3 | Odds ratio | 1.82 | | 2.01 | | - | | - | | 1.32 | | 1.30 | | - | | - |
|  |  | Lower CI | 0.78 | | 0.98 | | - | | - | | 0.57 | | 0.63 | | - | | - |
|  |  | Upper CI | 4.23 | | 4.15 | | - | | - | | 3.09 | | 2.68 | | - | | - |
|  |  | p value | 0.17 | | 0.06 | | - | | - | | 0.52 | | 0.48 | | - | | - |
|  | 4 vs 3 | Odds ratio | 1.76 | | 1.31 | | - | | - | | 1.12 | | 1.29 | | - | | - |
|  |  | Lower CI | 0.84 | | 0.65 | | - | | - | | 0.51 | | 0.59 | | - | | - |
|  |  | Upper CI | 3.69 | | 2.67 | | - | | - | | 2.42 | | 2.86 | | - | | - |
|  |  | p value | 0.13 | | 0.45 | | - | | - | | 0.78 | | 0.53 | | - | | - |
|  | 5 vs 3 | Odds ratio | 2.09 | | 0.93 | | - | | - | | 0.85 | | 0.93 | | - | | - |
|  |  | Lower CI | 0.90 | | 0.43 | | - | | - | | 0.33 | | 0.39 | | - | | - |
|  |  | Upper CI | 4.83 | | 2.04 | | - | | - | | 2.15 | | 2.22 | | - | | - |
|  |  | p value | 0.08 | | 0.86 | | - | | - | | 0.72 | | 0.88 | | - | | - |

| **Enteral** | 1 vs 3 | Odds ratio | - | 0.91 | 0.49 | 0.46 | - | 0.70 | 0.66 | 0.81 |
| --- | --- | --- | --- | --- | --- | --- | --- | --- | --- | --- |
|  |  | Lower CI | - | 0.43 | 0.22 | 0.22 | - | 0.30 | 0.27 | 0.35 |
|  |  | Upper CI | - | 1.91 | 1.10 | 0.97 | - | 1.62 | 1.60 | 1.89 |
|  |  | p value | - | 0.80 | 0.08 | **0.04** | - | 0.41 | 0.36 | 0.62 |
|  | 2 vs 3 | Odds ratio | - | 0.82 | 0.54 | 0.60 | - | 0.91 | 0.68 | 1.54 |
|  |  | Lower CI | - | 0.41 | 0.26 | 0.29 | - | 0.42 | 0.31 | 0.70 |
|  |  | Upper CI | - | 1.65 | 1.10 | 1.25 | - | 1.97 | 1.48 | 3.38 |
|  |  | p value | - | 0.58 | 0.09 | 0.17 | - | 0.81 | 0.33 | 0.28 |
|  | 4 vs 3 | Odds ratio | - | 0.82 | 1.03 | 1.29 | - | 0.85 | 2.01 | 1.70 |
|  |  | Lower CI | - | 0.41 | 0.51 | 0.63 | - | 0.41 | 0.97 | 0.80 |
|  |  | Upper CI | - | 1.66 | 2.09 | 2.64 | - | 1.76 | 4.16 | 3.62 |
|  |  | p value | - | 0.58 | 0.93 | 0.49 | - | 0.65 | 0.06 | 0.17 |
|  | 5 vs 3 | Odds ratio | - | 0.89 | 0.83 | 1.17 | - | 0.94 | 0.96 | 1.92 |
|  |  | Lower CI | - | 0.43 | 0.38 | 0.54 | - | 0.43 | 0.43 | 0.86 |
|  |  | Upper CI | - | 1.85 | 1.81 | 2.54 | - | 2.04 | 2.14 | 4.27 |
|  |  | p value | - | 0.75 | 0.64 | 0.68 | - | 0.87 | 0.91 | 0.11 |
| **Length expected growth** | | |  | | | |  | | | |
| **Carbohydrate** | | |  |  |  |  |  |  |  |  |
| **IV** | 1 vs 3 | Odds ratio | 0.57 | 0.71 | - | - | 0.57 | 0.86 | - | - |
|  |  | Lower CI | 0.27 | 0.33 | - | - | 0.25 | 0.39 | - | - |
|  |  | Upper CI | 1.23 | 1.52 | - | - | 1.30 | 1.88 | - | - |
|  |  | p value | 0.15 | 0.38 | - | - | 0.18 | 0.70 | - | - |
|  | 2 vs 3 | Odds ratio | 0.47 | 1.08 | - | - | 0.59 | 0.99 | - | - |
|  |  | Lower CI | 0.23 | 0.53 | - | - | 0.27 | 0.47 | - | - |
|  |  | Upper CI | 0.99 | 2.20 | - | - | 1.28 | 2.08 | - | - |
|  |  | p value | **0.05** | 0.83 | - | - | 0.18 | 0.98 | - | - |
|  | 4 vs 3 | Odds ratio | 0.85 | 1.00 | - | - | 0.33 | 0.93 | - | - |
|  |  | Lower CI | 0.38 | 0.48 | - | - | 0.13 | 0.42 | - | - |
|  |  | Upper CI | 1.93 | 2.07 | - | - | 0.82 | 2.06 | - | - |
|  |  | p value | 0.70 | 1.00 | - | - | **0.02** | 0.87 | - | - |
|  | 5 vs 3 | Odds ratio | 0.64 | 0.65 | - | - | 0.65 | 0.69 | - | - |
|  |  | Lower CI | 0.27 | 0.30 | - | - | 0.26 | 0.29 | - | - |
|  |  | Upper CI | 1.51 | 1.41 | - | - | 1.63 | 1.63 | - | - |
|  |  | p value | 0.30 | 0.28 | - | - | 0.35 | 0.40 | - | - |
| **Enteral** | 1 vs 3 | Odds ratio | - | 0.84 | 0.52 | 0.45 | - | 0.78 | 0.89 | 0.50 |
|  |  | Lower CI | - | 0.41 | 0.24 | 0.21 | - | 0.35 | 0.38 | 0.22 |
|  |  | Upper CI | - | 1.74 | 1.13 | 0.94 | - | 1.76 | 2.10 | 1.17 |
|  |  | p value | - | 0.64 | 0.10 | **0.03** | - | 0.55 | 0.79 | 0.11 |
|  | 2 vs 3 | Odds ratio | - | 0.77 | 0.83 | 0.48 | - | 1.03 | 1.07 | 0.85 |
|  |  | Lower CI | - | 0.39 | 0.41 | 0.23 | - | 0.48 | 0.50 | 0.38 |
|  |  | Upper CI | - | 1.54 | 1.67 | 1.03 | - | 2.22 | 2.31 | 1.90 |
|  |  | p value | - | 0.46 | 0.61 | 0.06 | - | 0.94 | 0.86 | 0.70 |
|  | 4 vs 3 | Odds ratio | - | 0.86 | 1.06 | 0.96 | - | 1.02 | 1.35 | 1.00 |
|  |  | Lower CI | - | 0.43 | 0.51 | 0.47 | - | 0.49 | 0.62 | 0.48 |
|  |  | Upper CI | - | 1.74 | 2.20 | 1.94 | - | 2.13 | 2.97 | 2.10 |
|  |  | p value | - | 0.68 | 0.88 | 0.90 | - | 0.95 | 0.45 | 1.00 |
|  | 5 vs 3 | Odds ratio | - | 0.70 | 1.07 | 1.27 | - | 1.02 | 2.25 | 1.18 |
|  |  | Lower CI | - | 0.34 | 0.49 | 0.58 | - | 0.46 | 0.99 | 0.52 |
|  |  | Upper CI | - | 1.48 | 2.33 | 2.79 | - | 2.25 | 5.11 | 2.65 |
|  |  | p value | - | 0.35 | 0.87 | 0.55 | - | 0.96 | 0.05 | 0.69 |
| **Length expected growth** | | |  | | | |  | | | |
| **Energy:protein ratio** | | |  |  |  |  |  |  |  |  |
| **Total** | 1 vs 3 | Odds ratio | 1.23 | 1.40 | 0.93 | 0.29 | 1.29 | 1.31 | 0.82 | 0.74 |
|  |  | Lower CI | 0.57 | 0.66 | 0.43 | 0.13 | 0.58 | 0.58 | 0.37 | 0.32 |
|  |  | Upper CI | 2.67 | 2.95 | 1.98 | 0.65 | 2.88 | 2.95 | 1.83 | 1.72 |
|  |  | p value | 0.60 | 0.38 | 0.84 | **<0.01** | 0.53 | 0.52 | 0.63 | 0.49 |
|  | 2 vs 3 | Odds ratio | 0.85 | 1.78 | 0.81 | 0.65 | 0.81 | 1.44 | 0.59 | 0.90 |
|  |  | Lower CI | 0.41 | 0.86 | 0.37 | 0.29 | 0.37 | 0.67 | 0.25 | 0.38 |
|  |  | Upper CI | 1.79 | 3.69 | 1.76 | 1.45 | 1.80 | 3.11 | 1.38 | 2.13 |
|  |  | p value | 0.68 | 0.12 | 0.60 | 0.29 | 0.61 | 0.36 | 0.22 | 0.81 |
|  | 4 vs 3 | Odds ratio | 0.61 | 0.97 | 1.20 | 0.69 | 0.76 | 1.14 | 0.87 | 1.24 |
|  |  | Lower CI | 0.30 | 0.46 | 0.58 | 0.31 | 0.35 | 0.51 | 0.41 | 0.55 |
|  |  | Upper CI | 1.25 | 2.03 | 2.48 | 1.53 | 1.63 | 2.52 | 1.84 | 2.83 |
|  |  | p value | 0.18 | 0.93 | 0.62 | 0.36 | 0.48 | 0.75 | 0.71 | 0.60 |
|  | 5 vs 3 | Odds ratio | 1.10 | 1.65 | 0.94 | 0.65 | 1.15 | 1.49 | 1.13 | 0.84 |
|  |  | Lower CI | 0.51 | 0.77 | 0.42 | 0.28 | 0.50 | 0.65 | 0.50 | 0.34 |
|  |  | Upper CI | 2.36 | 3.52 | 2.09 | 1.55 | 2.66 | 3.43 | 2.58 | 2.06 |
|  |  | p value | 0.80 | 0.20 | 0.87 | 0.33 | 0.75 | 0.35 | 0.77 | 0.70 |

| **IV** | 1 vs 3 | Odds ratio | 0.70 | 1.71 | - | - | 1.45 | 1.12 | - | - |
| --- | --- | --- | --- | --- | --- | --- | --- | --- | --- | --- |
|  |  | Lower CI | 0.31 | 0.68 | - | - | 0.64 | 0.46 | - | - |
|  |  | Upper CI | 1.57 | 4.29 | - | - | 3.28 | 2.77 | - | - |
|  |  | p value | 0.39 | 0.25 | - | - | 0.38 | 0.80 | - | - |
|  | 2 vs 3 | Odds ratio | 0.60 | 2.36 | - | - | 0.72 | 0.80 | - | - |
|  |  | Lower CI | 0.29 | 1.05 | - | - | 0.33 | 0.35 | - | - |
|  |  | Upper CI | 1.23 | 5.29 | - | - | 1.56 | 1.83 | - | - |
|  |  | p value | 0.16 | **0.04** | - | - | 0.40 | 0.60 | - | - |
|  | 4 vs 3 | Odds ratio | 0.34 | 1.26 | - | - | 0.72 | 0.50 | - | - |
|  |  | Lower CI | 0.16 | 0.55 | - | - | 0.33 | 0.21 | - | - |
|  |  | Upper CI | 0.71 | 2.89 | - | - | 1.57 | 1.20 | - | - |
|  |  | p value | **<0.01** | 0.59 | - | - | 0.41 | 0.12 | - | - |
|  | 5 vs 3 | Odds ratio | 0.56 | 1.58 | - | - | 0.91 | 0.87 | - | - |
|  |  | Lower CI | 0.25 | 0.72 | - | - | 0.38 | 0.38 | - | - |
|  |  | Upper CI | 1.25 | 3.47 | - | - | 2.18 | 1.99 | - | - |
|  |  | p value | 0.15 | 0.26 | - | - | 0.84 | 0.74 | - | - |
| **Enteral** | 1 vs 3 | Odds ratio | - | 0.55 | 0.84 | 0.78 | - | 0.97 | 1.17 | 0.34 |
|  |  | Lower CI | - | 0.24 | 0.40 | 0.35 | - | 0.40 | 0.52 | 0.14 |
|  |  | Upper CI | - | 1.28 | 1.80 | 1.74 | - | 2.36 | 2.64 | 0.84 |
|  |  | p value | - | 0.16 | 0.66 | 0.54 | - | 0.95 | 0.70 | **0.02** |
|  | 2 vs 3 | Odds ratio | - | 0.81 | 0.85 | 1.08 | - | 1.02 | 1.44 | 0.54 |
|  |  | Lower CI | - | 0.38 | 0.40 | 0.51 | - | 0.46 | 0.66 | 0.25 |
|  |  | Upper CI | - | 1.72 | 1.81 | 2.29 | - | 2.27 | 3.16 | 1.16 |
|  |  | p value | - | 0.58 | 0.67 | 0.85 | - | 0.96 | 0.36 | 0.11 |
|  | 4 vs 3 | Odds ratio | - | 0.79 | 0.61 | 0.67 | - | 0.85 | 0.73 | 0.34 |
|  |  | Lower CI | - | 0.37 | 0.28 | 0.30 | - | 0.37 | 0.31 | 0.14 |
|  |  | Upper CI | - | 1.70 | 1.33 | 1.48 | - | 1.94 | 1.75 | 0.82 |
|  |  | p value | - | 0.55 | 0.21 | 0.32 | - | 0.70 | 0.48 | **0.02** |
|  | 5 vs 3 | Odds ratio | - | 0.62 | 0.92 | 0.62 | - | 0.77 | 0.66 | 0.40 |
|  |  | Lower CI | - | 0.30 | 0.37 | 0.26 | - | 0.34 | 0.25 | 0.16 |
|  |  | Upper CI | - | 1.30 | 2.25 | 1.47 | - | 1.71 | 1.80 | 1.03 |
|  |  | p value | - | 0.21 | 0.85 | 0.28 | - | 0.52 | 0.42 | 0.06 |

| **Head circumference expected growth** | | | **Birth to 4 weeks** | | | | **Birth to 36 weeks corrected age** | | | |
| --- | --- | --- | --- | --- | --- | --- | --- | --- | --- | --- |
| **Predictor** | **Quintile** | **Value** | **Week 1** | **Week 2** | **Week 3** | **Week 4** | **Week 1** | **Week 2** | **Week 3** | **Week 4** |
| **Fluid** | | |  |  |  |  |  |  |  |  |
| **Total** | 1 vs 3 | Odds ratio | 1.66 | 1.64 | 1.59 | 2.02 | 0.77 | 0.73 | 0.52 | 0.51 |
|  |  | Lower CI | 0.70 | 0.73 | 0.72 | 0.93 | 0.32 | 0.32 | 0.24 | 0.24 |
|  |  | Upper CI | 3.94 | 3.69 | 3.49 | 4.39 | 1.85 | 1.66 | 1.11 | 1.07 |
|  |  | p value | 0.25 | 0.23 | 0.25 | 0.08 | 0.56 | 0.45 | 0.09 | 0.08 |
|  | 2 vs 3 | Odds ratio | 0.93 | 1.48 | 0.95 | 1.92 | 1.34 | 1.03 | 0.92 | 1.63 |
|  |  | Lower CI | 0.43 | 0.67 | 0.44 | 0.89 | 0.64 | 0.48 | 0.45 | 0.80 |
|  |  | Upper CI | 2.01 | 3.28 | 2.05 | 4.18 | 2.80 | 2.22 | 1.91 | 3.32 |
|  |  | p value | 0.85 | 0.34 | 0.89 | 0.10 | 0.44 | 0.94 | 0.83 | 0.18 |
|  | 4 vs 3 | Odds ratio | 0.73 | 1.46 | 0.98 | 1.29 | 0.65 | 1.43 | 0.78 | 0.54 |
|  |  | Lower CI | 0.35 | 0.65 | 0.44 | 0.56 | 0.33 | 0.70 | 0.36 | 0.25 |
|  |  | Upper CI | 1.56 | 3.28 | 2.20 | 2.94 | 1.31 | 2.95 | 1.66 | 1.15 |
|  |  | p value | 0.42 | 0.36 | 0.96 | 0.55 | 0.23 | 0.33 | 0.51 | 0.11 |
|  | 5 vs 3 | Odds ratio | 0.76 | 1.08 | 1.23 | 0.99 | 0.72 | 1.29 | 0.93 | 1.10 |
|  |  | Lower CI | 0.31 | 0.46 | 0.56 | 0.42 | 0.33 | 0.60 | 0.45 | 0.52 |
|  |  | Upper CI | 1.84 | 2.52 | 2.70 | 2.33 | 1.61 | 2.79 | 1.93 | 2.33 |
|  |  | p value | 0.54 | 0.86 | 0.61 | 0.99 | 0.43 | 0.52 | 0.85 | 0.81 |
| **IV** | 1 vs 3 | Odds ratio | 1.61 | 1.75 | 1.28 | - | 0.59 | 0.59 | 1.21 | - |
|  |  | Lower CI | 0.73 | 0.81 | 0.62 | - | 0.27 | 0.28 | 0.61 | - |
|  |  | Upper CI | 3.55 | 3.80 | 2.64 | - | 1.28 | 1.26 | 2.41 | - |
|  |  | p value | 0.24 | 0.15 | 0.50 | - | 0.18 | 0.17 | 0.59 | - |
|  | 2 vs 3 | Odds ratio | 0.72 | 1.34 | 1.51 | 0.95 | 0.80 | 0.75 | 2.24 | 0.81 |
|  |  | Lower CI | 0.34 | 0.64 | 0.61 | 0.43 | 0.39 | 0.37 | 0.93 | 0.39 |
|  |  | Upper CI | 1.53 | 2.82 | 3.76 | 2.07 | 1.64 | 1.54 | 5.42 | 1.69 |
|  |  | p value | 0.39 | 0.44 | 0.38 | 0.89 | 0.53 | 0.44 | 0.07 | 0.58 |
|  | 4 vs 3 | Odds ratio | 0.53 | 1.47 | 0.54 | 1.01 | 0.66 | 0.83 | 0.73 | 0.46 |
|  |  | Lower CI | 0.24 | 0.66 | 0.24 | 0.43 | 0.32 | 0.39 | 0.35 | 0.20 |
|  |  | Upper CI | 1.18 | 3.28 | 1.24 | 2.39 | 1.38 | 1.78 | 1.52 | 1.03 |
|  |  | p value | 0.12 | 0.35 | 0.15 | 0.98 | 0.27 | 0.63 | 0.40 | 0.06 |
|  | 5 vs 3 | Odds ratio | 0.71 | 1.05 | 1.24 | 1.03 | 0.61 | 0.35 | 0.42 | 0.22 |
|  |  | Lower CI | 0.29 | 0.45 | 0.53 | 0.43 | 0.27 | 0.15 | 0.19 | 0.09 |
|  |  | Upper CI | 1.70 | 2.47 | 2.88 | 2.46 | 1.36 | 0.80 | 0.95 | 0.52 |
|  |  | p value | 0.44 | 0.91 | 0.62 | 0.95 | 0.22 | **0.01** | **0.04** | **<0.01** |

| **Enteral** | 1 vs 3 | Odds ratio | 1.05 | 1.18 | 0.46 | 1.19 | 0.79 | 0.75 | 0.22 | 0.22 |
| --- | --- | --- | --- | --- | --- | --- | --- | --- | --- | --- |
|  |  | Lower CI | 0.45 | 0.52 | 0.19 | 0.54 | 0.36 | 0.35 | 0.09 | 0.10 |
|  |  | Upper CI | 2.45 | 2.71 | 1.13 | 2.60 | 1.72 | 1.64 | 0.52 | 0.49 |
|  |  | p value | 0.92 | 0.69 | 0.09 | 0.66 | 0.55 | 0.48 | **<0.01** | **<0.001** |
|  | 2 vs 3 | Odds ratio | 1.64 | 1.06 | 0.53 | 1.00 | 1.64 | 0.97 | 0.49 | 0.36 |
|  |  | Lower CI | 0.76 | 0.49 | 0.25 | 0.46 | 0.79 | 0.46 | 0.23 | 0.17 |
|  |  | Upper CI | 3.54 | 2.29 | 1.14 | 2.15 | 3.41 | 2.06 | 1.02 | 0.77 |
|  |  | p value | 0.21 | 0.88 | 0.11 | 0.99 | 0.18 | 0.94 | 0.06 | **0.01** |
|  | 4 vs 3 | Odds ratio | 1.28 | 1.08 | 0.92 | 0.73 | 1.15 | 1.23 | 0.83 | 0.38 |
|  |  | Lower CI | 0.60 | 0.52 | 0.44 | 0.35 | 0.55 | 0.61 | 0.40 | 0.19 |
|  |  | Upper CI | 2.75 | 2.26 | 1.89 | 1.55 | 2.38 | 2.51 | 1.69 | 0.79 |
|  |  | p value | 0.52 | 0.84 | 0.81 | 0.42 | 0.71 | 0.56 | 0.60 | **0.01** |
|  | 5 vs 3 | Odds ratio | 1.16 | 1.46 | 0.52 | 0.63 | 0.73 | 1.11 | 0.64 | 0.60 |
|  |  | Lower CI | 0.55 | 0.68 | 0.23 | 0.27 | 0.35 | 0.52 | 0.30 | 0.27 |
|  |  | Upper CI | 2.46 | 3.16 | 1.19 | 1.46 | 1.51 | 2.33 | 1.37 | 1.33 |
|  |  | p value | 0.70 | 0.33 | 0.12 | 0.28 | 0.39 | 0.79 | 0.25 | 0.21 |
| **Breastmilk** | 1 vs 3 | Odds ratio | 0.98 | 1.45 | 0.86 | 3.09 | 0.88 | 0.71 | 0.52 | 0.56 |
|  |  | Lower CI | 0.42 | 0.65 | 0.36 | 1.35 | 0.40 | 0.33 | 0.23 | 0.26 |
|  |  | Upper CI | 2.31 | 3.24 | 2.01 | 7.03 | 1.91 | 1.52 | 1.16 | 1.20 |
|  |  | p value | 0.97 | 0.37 | 0.72 | **0.01** | 0.74 | 0.37 | 0.11 | 0.14 |
|  | 2 vs 3 | Odds ratio | 1.63 | 0.97 | 0.66 | 2.23 | 1.60 | 0.87 | 1.08 | 0.59 |
|  |  | Lower CI | 0.76 | 0.44 | 0.31 | 0.99 | 0.77 | 0.42 | 0.52 | 0.28 |
|  |  | Upper CI | 3.50 | 2.11 | 1.42 | 5.02 | 3.31 | 1.82 | 2.23 | 1.24 |
|  |  | p value | 0.21 | 0.93 | 0.29 | 0.05 | 0.21 | 0.71 | 0.83 | 0.16 |
|  | 4 vs 3 | Odds ratio | 1.29 | 1.09 | 0.83 | 1.83 | 1.30 | 1.10 | 1.74 | 1.02 |
|  |  | Lower CI | 0.60 | 0.52 | 0.40 | 0.85 | 0.63 | 0.54 | 0.84 | 0.50 |
|  |  | Upper CI | 2.78 | 2.27 | 1.75 | 3.91 | 2.71 | 2.23 | 3.61 | 2.06 |
|  |  | p value | 0.51 | 0.82 | 0.63 | 0.12 | 0.48 | 0.79 | 0.14 | 0.96 |
|  | 5 vs 3 | Odds ratio | 1.15 | 1.30 | 0.63 | 1.65 | 0.77 | 0.88 | 1.51 | 1.15 |
|  |  | Lower CI | 0.54 | 0.60 | 0.28 | 0.69 | 0.37 | 0.42 | 0.71 | 0.55 |
|  |  | Upper CI | 2.45 | 2.82 | 1.40 | 4.00 | 1.59 | 1.85 | 3.20 | 2.43 |
|  |  | p value | 0.71 | 0.50 | 0.26 | 0.26 | 0.48 | 0.74 | 0.29 | 0.71 |
| **Head circumference expected growth** | | |  | | | |  | | | |
| **Energy** | | |  |  |  |  |  |  |  |  |
| **Total** | 1 vs 3 | Odds ratio | 0.64 | 0.93 | 0.42 | 1.58 | 0.46 | 0.78 | 0.30 | 0.29 |
|  |  | Lower CI | 0.26 | 0.41 | 0.17 | 0.71 | 0.19 | 0.37 | 0.13 | 0.14 |
|  |  | Upper CI | 1.56 | 2.07 | 1.05 | 3.51 | 1.14 | 1.68 | 0.69 | 0.64 |
|  |  | p value | 0.32 | 0.85 | 0.06 | 0.26 | 0.09 | 0.53 | **<0.01** | **<0.01** |
|  | 2 vs 3 | Odds ratio | 0.66 | 1.20 | 0.89 | 1.99 | 0.77 | 1.06 | 1.20 | 0.54 |
|  |  | Lower CI | 0.31 | 0.55 | 0.42 | 0.88 | 0.36 | 0.50 | 0.60 | 0.26 |
|  |  | Upper CI | 1.40 | 2.65 | 1.89 | 4.49 | 1.63 | 2.27 | 2.42 | 1.13 |
|  |  | p value | 0.28 | 0.65 | 0.77 | 0.10 | 0.49 | 0.88 | 0.61 | 0.10 |
|  | 4 vs 3 | Odds ratio | 0.65 | 1.34 | 0.80 | 1.49 | 0.53 | 1.56 | 1.31 | 0.86 |
|  |  | Lower CI | 0.30 | 0.61 | 0.38 | 0.70 | 0.26 | 0.75 | 0.64 | 0.42 |
|  |  | Upper CI | 1.39 | 2.93 | 1.70 | 3.20 | 1.10 | 3.27 | 2.71 | 1.78 |
|  |  | p value | 0.26 | 0.47 | 0.56 | 0.30 | 0.09 | 0.24 | 0.46 | 0.69 |
|  | 5 vs 3 | Odds ratio | 0.67 | 1.62 | 1.21 | 0.91 | 0.54 | 1.59 | 1.26 | 1.04 |
|  |  | Lower CI | 0.30 | 0.74 | 0.55 | 0.39 | 0.25 | 0.73 | 0.59 | 0.48 |
|  |  | Upper CI | 1.52 | 3.56 | 2.66 | 2.13 | 1.18 | 3.50 | 2.71 | 2.29 |
|  |  | p value | 0.34 | 0.23 | 0.64 | 0.83 | 0.12 | 0.25 | 0.55 | 0.91 |
| **IV** | 1 vs 3 | Odds ratio | 1.57 | 2.05 | 1.64 | - | 0.95 | 1.02 | 0.84 | - |
|  |  | Lower CI | 0.66 | 0.95 | 0.80 | - | 0.41 | 0.49 | 0.43 | - |
|  |  | Upper CI | 3.77 | 4.41 | 3.38 | - | 2.21 | 2.14 | 1.65 | - |
|  |  | p value | 0.31 | 0.07 | 0.18 | - | 0.91 | 0.96 | 0.62 | - |
|  | 2 vs 3 | Odds ratio | 1.69 | 1.35 | 1.94 | 0.80 | 0.47 | 1.36 | 0.81 | 0.39 |
|  |  | Lower CI | 0.79 | 0.64 | 0.63 | 0.33 | 0.22 | 0.68 | 0.27 | 0.16 |
|  |  | Upper CI | 3.60 | 2.87 | 5.96 | 1.98 | 0.99 | 2.74 | 2.43 | 0.98 |
|  |  | p value | 0.18 | 0.44 | 0.25 | 0.63 | **0.05** | 0.39 | 0.71 | **0.04** |
|  | 4 vs 3 | Odds ratio | 1.39 | 1.03 | 0.68 | 0.62 | 0.64 | 1.30 | 0.48 | 0.23 |
|  |  | Lower CI | 0.62 | 0.46 | 0.30 | 0.23 | 0.29 | 0.62 | 0.23 | 0.08 |
|  |  | Upper CI | 3.14 | 2.34 | 1.57 | 1.68 | 1.41 | 2.73 | 0.99 | 0.61 |
|  |  | p value | 0.43 | 0.94 | 0.36 | 0.34 | 0.26 | 0.50 | **0.05** | **<0.01** |
|  | 5 vs 3 | Odds ratio | 1.41 | 2.05 | 1.31 | 1.23 | 0.85 | 1.27 | 0.37 | 0.13 |
|  |  | Lower CI | 0.56 | 0.89 | 0.56 | 0.47 | 0.37 | 0.58 | 0.17 | 0.05 |
|  |  | Upper CI | 3.54 | 4.70 | 3.09 | 3.22 | 1.95 | 2.78 | 0.81 | 0.35 |
|  |  | p value | 0.46 | 0.09 | 0.54 | 0.68 | 0.70 | 0.56 | **0.01** | **<0.0001** |

| **Enteral** | 1 vs 3 | Odds ratio | 1.03 | 1.23 | 0.86 | 1.44 | 0.73 | 0.71 | 0.52 | 0.31 |
| --- | --- | --- | --- | --- | --- | --- | --- | --- | --- | --- |
|  |  | Lower CI | 0.44 | 0.54 | 0.34 | 0.66 | 0.34 | 0.33 | 0.23 | 0.15 |
|  |  | Upper CI | 2.41 | 2.82 | 2.17 | 3.17 | 1.59 | 1.54 | 1.20 | 0.68 |
|  |  | p value | 0.94 | 0.63 | 0.74 | 0.36 | 0.43 | 0.39 | 0.13 | **<0.01** |
|  | 2 vs 3 | Odds ratio | 1.62 | 0.93 | 1.22 | 1.10 | 1.53 | 0.84 | 1.57 | 0.49 |
|  |  | Lower CI | 0.75 | 0.43 | 0.55 | 0.49 | 0.74 | 0.40 | 0.75 | 0.23 |
|  |  | Upper CI | 3.49 | 2.03 | 2.74 | 2.45 | 3.17 | 1.78 | 3.25 | 1.01 |
|  |  | p value | 0.22 | 0.86 | 0.63 | 0.82 | 0.25 | 0.65 | 0.23 | 0.05 |
|  | 4 vs 3 | Odds ratio | 1.26 | 1.07 | 1.75 | 1.25 | 1.01 | 1.20 | 2.60 | 0.84 |
|  |  | Lower CI | 0.59 | 0.51 | 0.82 | 0.60 | 0.49 | 0.59 | 1.23 | 0.41 |
|  |  | Upper CI | 2.70 | 2.27 | 3.77 | 2.63 | 2.09 | 2.44 | 5.50 | 1.71 |
|  |  | p value | 0.55 | 0.86 | 0.15 | 0.55 | 0.99 | 0.61 | **0.01** | 0.63 |
|  | 5 vs 3 | Odds ratio | 1.13 | 1.47 | 1.71 | 0.76 | 0.66 | 0.83 | 1.64 | 0.97 |
|  |  | Lower CI | 0.53 | 0.68 | 0.75 | 0.33 | 0.32 | 0.39 | 0.75 | 0.45 |
|  |  | Upper CI | 2.41 | 3.19 | 3.90 | 1.74 | 1.37 | 1.78 | 3.59 | 2.11 |
|  |  | p value | 0.75 | 0.33 | 0.21 | 0.51 | 0.27 | 0.63 | 0.22 | 0.94 |
| **Head circumference expected growth** | | |  | | | |  | | | |
| **Protein** | | |  |  |  |  |  |  |  |  |
| **Total** | 1 vs 3 | Odds ratio | 0.56 | 0.58 | 0.28 | 0.66 | 1.10 | 0.40 | 1.67 | 0.58 |
|  |  | Lower CI | 0.22 | 0.25 | 0.10 | 0.25 | 0.51 | 0.17 | 0.74 | 0.24 |
|  |  | Upper CI | 1.46 | 1.36 | 0.78 | 1.73 | 2.39 | 0.92 | 3.77 | 1.39 |
|  |  | p value | 0.23 | 0.21 | **0.01** | 0.39 | 0.81 | **0.03** | 0.22 | 0.22 |
|  | 2 vs 3 | Odds ratio | 0.91 | 0.19 | 0.78 | 0.99 | 1.05 | 0.36 | 1.47 | 0.63 |
|  |  | Lower CI | 0.42 | 0.07 | 0.35 | 0.46 | 0.50 | 0.16 | 0.69 | 0.29 |
|  |  | Upper CI | 1.98 | 0.50 | 1.71 | 2.13 | 2.21 | 0.79 | 3.12 | 1.36 |
|  |  | p value | 0.81 | **<0.01** | 0.53 | 0.97 | 0.90 | **0.01** | 0.31 | 0.24 |
|  | 4 vs 3 | Odds ratio | 1.43 | 0.65 | 1.34 | 1.09 | 0.82 | 0.95 | 2.24 | 1.89 |
|  |  | Lower CI | 0.68 | 0.31 | 0.64 | 0.52 | 0.40 | 0.45 | 1.05 | 0.90 |
|  |  | Upper CI | 3.01 | 1.35 | 2.83 | 2.26 | 1.70 | 2.04 | 4.78 | 3.96 |
|  |  | p value | 0.35 | 0.25 | 0.44 | 0.83 | 0.60 | 0.90 | **0.04** | 0.09 |
|  | 5 vs 3 | Odds ratio | 1.23 | 0.78 | 1.92 | 0.84 | 1.15 | 0.88 | 1.74 | 1.58 |
|  |  | Lower CI | 0.57 | 0.34 | 0.88 | 0.39 | 0.54 | 0.38 | 0.80 | 0.73 |
|  |  | Upper CI | 2.66 | 1.78 | 4.18 | 1.82 | 2.45 | 2.02 | 3.77 | 3.41 |
|  |  | p value | 0.60 | 0.55 | 0.10 | 0.66 | 0.73 | 0.76 | 0.16 | 0.24 |
| **IV** | 1 vs 3 | Odds ratio | 0.85 | 1.70 | - | - | 1.15 | 0.84 | - | - |
|  |  | Lower CI | 0.36 | 0.79 | - | - | 0.54 | 0.40 | - | - |
|  |  | Upper CI | 2.04 | 3.68 | - | - | 2.45 | 1.78 | - | - |
|  |  | p value | 0.72 | 0.18 | - | - | 0.72 | 0.65 | - | - |
|  | 2 vs 3 | Odds ratio | 0.74 | 0.92 | - | - | 0.85 | 0.70 | - | - |
|  |  | Lower CI | 0.33 | 0.42 | - | - | 0.41 | 0.34 | - | - |
|  |  | Upper CI | 1.68 | 2.00 | - | - | 1.78 | 1.43 | - | - |
|  |  | p value | 0.47 | 0.83 | - | - | 0.66 | 0.32 | - | - |
|  | 4 vs 3 | Odds ratio | 1.65 | 1.02 | - | - | 1.13 | 0.61 | - | - |
|  |  | Lower CI | 0.78 | 0.45 | - | - | 0.55 | 0.29 | - | - |
|  |  | Upper CI | 3.50 | 2.32 | - | - | 2.34 | 1.29 | - | - |
|  |  | p value | 0.19 | 0.96 | - | - | 0.74 | 0.20 | - | - |
|  | 5 vs 3 | Odds ratio | 1.10 | 1.24 | - | - | 1.11 | 0.79 | - | - |
|  |  | Lower CI | 0.50 | 0.57 | - | - | 0.52 | 0.38 | - | - |
|  |  | Upper CI | 2.42 | 2.67 | - | - | 2.40 | 1.65 | - | - |
|  |  | p value | 0.82 | 0.59 | - | - | 0.79 | 0.52 | - | - |
| **Enteral** | 1 vs 3 | Odds ratio | 1.05 | 1.38 | 1.00 | 1.17 | 0.74 | 0.82 | 0.31 | 0.32 |
|  |  | Lower CI | 0.45 | 0.60 | 0.41 | 0.53 | 0.34 | 0.38 | 0.13 | 0.14 |
|  |  | Upper CI | 2.46 | 3.16 | 2.41 | 2.58 | 1.60 | 1.76 | 0.70 | 0.73 |
|  |  | p value | 0.91 | 0.45 | 1.00 | 0.69 | 0.44 | 0.61 | **0.01** | **0.01** |
|  | 2 vs 3 | Odds ratio | 1.64 | 1.20 | 0.67 | 0.86 | 1.57 | 1.05 | 0.80 | 0.62 |
|  |  | Lower CI | 0.76 | 0.55 | 0.29 | 0.37 | 0.76 | 0.50 | 0.39 | 0.28 |
|  |  | Upper CI | 3.55 | 2.64 | 1.52 | 2.01 | 3.24 | 2.20 | 1.65 | 1.35 |
|  |  | p value | 0.21 | 0.64 | 0.34 | 0.73 | 0.23 | 0.91 | 0.55 | 0.23 |
|  | 4 vs 3 | Odds ratio | 1.29 | 1.34 | 1.67 | 1.08 | 0.89 | 1.19 | 1.45 | 1.85 |
|  |  | Lower CI | 0.60 | 0.63 | 0.79 | 0.52 | 0.43 | 0.58 | 0.69 | 0.89 |
|  |  | Upper CI | 2.75 | 2.85 | 3.55 | 2.26 | 1.84 | 2.45 | 3.04 | 3.85 |
|  |  | p value | 0.52 | 0.46 | 0.18 | 0.83 | 0.75 | 0.63 | 0.32 | 0.10 |
|  | 5 vs 3 | Odds ratio | 1.18 | 1.82 | 1.67 | 0.87 | 0.78 | 1.26 | 1.10 | 1.33 |
|  |  | Lower CI | 0.55 | 0.83 | 0.75 | 0.39 | 0.38 | 0.59 | 0.51 | 0.62 |
|  |  | Upper CI | 2.52 | 4.02 | 3.70 | 1.94 | 1.62 | 2.73 | 2.39 | 2.87 |
|  |  | p value | 0.67 | 0.14 | 0.21 | 0.73 | 0.50 | 0.55 | 0.81 | 0.46 |

| **Head circumference expected growth** | | |  | | | |  | | | |
| --- | --- | --- | --- | --- | --- | --- | --- | --- | --- | --- |
| **Fat** | | |  |  |  |  |  |  |  |  |
| **IV** | 1 vs 3 | Odds ratio | 1.42 | 1.56 | - | - | 1.49 | 0.99 | - | - |
|  |  | Lower CI | 0.59 | 0.73 | - | - | 0.65 | 0.47 | - | - |
|  |  | Upper CI | 3.40 | 3.32 | - | - | 3.41 | 2.08 | - | - |
|  |  | p value | 0.43 | 0.26 | - | - | 0.34 | 0.98 | - | - |
|  | 2 vs 3 | Odds ratio | 1.57 | 1.07 | - | - | 1.65 | 1.00 | - | - |
|  |  | Lower CI | 0.66 | 0.50 | - | - | 0.72 | 0.50 | - | - |
|  |  | Upper CI | 3.74 | 2.29 | - | - | 3.78 | 2.01 | - | - |
|  |  | p value | 0.30 | 0.87 | - | - | 0.24 | 1.00 | - | - |
|  | 4 vs 3 | Odds ratio | 1.16 | 0.94 | - | - | 0.89 | 1.23 | - | - |
|  |  | Lower CI | 0.53 | 0.42 | - | - | 0.42 | 0.57 | - | - |
|  |  | Upper CI | 2.53 | 2.07 | - | - | 1.90 | 2.65 | - | - |
|  |  | p value | 0.71 | 0.87 | - | - | 0.77 | 0.60 | - | - |
|  | 5 vs 3 | Odds ratio | 0.83 | 1.95 | - | - | 1.01 | 1.20 | - | - |
|  |  | Lower CI | 0.31 | 0.77 | - | - | 0.43 | 0.54 | - | - |
|  |  | Upper CI | 2.22 | 4.99 | - | - | 2.35 | 2.67 | - | - |
|  |  | p value | 0.72 | 0.16 | - | - | 0.99 | 0.65 | - | - |
| **Enteral** | 1 vs 3 | Odds ratio | - | 1.14 | 0.46 | 1.37 | - | 0.63 | 0.18 | 0.30 |
|  |  | Lower CI | - | 0.50 | 0.18 | 0.63 | - | 0.29 | 0.08 | 0.13 |
|  |  | Upper CI | - | 2.62 | 1.13 | 3.02 | - | 1.37 | 0.43 | 0.66 |
|  |  | p value | - | 0.75 | 0.09 | 0.43 | - | 0.25 | **<0.0001** | **<0.01** |
|  | 2 vs 3 | Odds ratio | - | 0.73 | 0.53 | 1.04 | - | 0.75 | 0.37 | 0.50 |
|  |  | Lower CI | - | 0.34 | 0.25 | 0.47 | - | 0.36 | 0.17 | 0.24 |
|  |  | Upper CI | - | 1.59 | 1.16 | 2.26 | - | 1.59 | 0.77 | 1.06 |
|  |  | p value | - | 0.43 | 0.11 | 0.93 | - | 0.46 | **0.01** | 0.07 |
|  | 4 vs 3 | Odds ratio | - | 1.05 | 0.93 | 1.00 | - | 1.08 | 0.66 | 0.58 |
|  |  | Lower CI | - | 0.51 | 0.45 | 0.47 | - | 0.53 | 0.32 | 0.28 |
|  |  | Upper CI | - | 2.18 | 1.93 | 2.12 | - | 2.18 | 1.34 | 1.18 |
|  |  | p value | - | 0.89 | 0.85 | 0.99 | - | 0.84 | 0.25 | 0.13 |
|  | 5 vs 3 | Odds ratio | - | 1.12 | 0.51 | 0.85 | - | 0.85 | 0.50 | 1.05 |
|  |  | Lower CI | - | 0.52 | 0.22 | 0.37 | - | 0.40 | 0.23 | 0.48 |
|  |  | Upper CI | - | 2.40 | 1.17 | 1.95 | - | 1.79 | 1.08 | 2.30 |
|  |  | p value | - | 0.77 | 0.11 | 0.70 | - | 0.66 | 0.08 | 0.90 |
| **Head circumference** | | |  | | | |  | | | |
| **Carbohydrate** | | |  |  |  |  |  |  |  |  |
| **IV** | 1 vs 3 | Odds ratio | 1.86 | 2.20 | - | - | 0.59 | 0.60 | - | - |
|  |  | Lower CI | 0.83 | 1.02 | - | - | 0.27 | 0.28 | - | - |
|  |  | Upper CI | 4.16 | 4.78 | - | - | 1.31 | 1.27 | - | - |
|  |  | p value | 0.13 | **0.05** | - | - | 0.19 | 0.18 | - | - |
|  | 2 vs 3 | Odds ratio | 2.54 | 1.11 | - | - | 1.18 | 0.69 | - | - |
|  |  | Lower CI | 1.19 | 0.52 | - | - | 0.56 | 0.34 | - | - |
|  |  | Upper CI | 5.42 | 2.38 | - | - | 2.46 | 1.40 | - | - |
|  |  | p value | **0.02** | 0.79 | - | - | 0.66 | 0.30 | - | - |
|  | 4 vs 3 | Odds ratio | 1.95 | 1.09 | - | - | 0.88 | 0.66 | - | - |
|  |  | Lower CI | 0.78 | 0.49 | - | - | 0.38 | 0.31 | - | - |
|  |  | Upper CI | 4.88 | 2.46 | - | - | 2.05 | 1.43 | - | - |
|  |  | p value | 0.16 | 0.83 | - | - | 0.76 | 0.29 | - | - |
|  | 5 vs 3 | Odds ratio | 2.27 | 1.87 | - | - | 1.24 | 0.64 | - | - |
|  |  | Lower CI | 0.83 | 0.80 | - | - | 0.51 | 0.29 | - | - |
|  |  | Upper CI | 6.26 | 4.36 | - | - | 2.99 | 1.42 | - | - |
|  |  | p value | 0.11 | 0.15 | - | - | 0.64 | 0.27 | - | - |
| **Enteral** | 1 vs 3 | Odds ratio | - | 1.30 | 0.76 | 1.36 | - | 0.71 | 0.31 | 0.29 |
|  |  | Lower CI | - | 0.57 | 0.31 | 0.62 | - | 0.33 | 0.14 | 0.13 |
|  |  | Upper CI | - | 2.95 | 1.87 | 3.00 | - | 1.51 | 0.71 | 0.65 |
|  |  | p value | - | 0.54 | 0.54 | 0.44 | - | 0.37 | **0.01** | **<0.01** |
|  | 2 vs 3 | Odds ratio | - | 0.96 | 0.95 | 0.89 | - | 0.96 | 0.86 | 0.56 |
|  |  | Lower CI | - | 0.44 | 0.43 | 0.38 | - | 0.46 | 0.42 | 0.26 |
|  |  | Upper CI | - | 2.10 | 2.09 | 2.09 | - | 2.01 | 1.75 | 1.22 |
|  |  | p value | - | 0.92 | 0.90 | 0.79 | - | 0.91 | 0.68 | 0.15 |
|  | 4 vs 3 | Odds ratio | - | 1.18 | 1.39 | 1.22 | - | 1.17 | 1.29 | 1.29 |
|  |  | Lower CI | - | 0.56 | 0.65 | 0.59 | - | 0.58 | 0.61 | 0.63 |
|  |  | Upper CI | - | 2.49 | 2.99 | 2.54 | - | 2.36 | 2.73 | 2.65 |
|  |  | p value | - | 0.67 | 0.40 | 0.59 | - | 0.67 | 0.51 | 0.48 |
|  | 5 vs 3 | Odds ratio | - | 1.80 | 1.56 | 0.78 | - | 1.21 | 1.14 | 0.94 |
|  |  | Lower CI | - | 0.83 | 0.70 | 0.34 | - | 0.57 | 0.52 | 0.43 |
|  |  | Upper CI | - | 3.89 | 3.51 | 1.80 | - | 2.58 | 2.49 | 2.06 |
|  |  | p value | - | 0.14 | 0.28 | 0.57 | - | 0.63 | 0.75 | 0.88 |

| **Head circumference expected growth** | | |  | | | | | | |  | | | | | | | |
| --- | --- | --- | --- | --- | --- | --- | --- | --- | --- | --- | --- | --- | --- | --- | --- | --- | --- |
| **Energy:protein ratio** | | |  |  | |  | |  | |  | |  | |  | |  | |
| **Total** | 1 vs 3 | Odds ratio | 0.67 | | 0.64 | | 1.15 | | 2.28 | | 0.88 | | 1.26 | | 0.41 | | 0.84 |
|  |  | Lower CI | 0.31 | | 0.30 | | 0.52 | | 1.03 | | 0.40 | | 0.57 | | 0.19 | | 0.38 |
|  |  | Upper CI | 1.48 | | 1.40 | | 2.52 | | 5.04 | | 1.95 | | 2.76 | | 0.90 | | 1.87 |
|  |  | p value | 0.33 | | 0.26 | | 0.73 | | **0.04** | | 0.76 | | 0.56 | | **0.03** | | 0.68 |
|  | 2 vs 3 | Odds ratio | 0.65 | | 0.89 | | 1.13 | | 1.35 | | 0.57 | | 1.99 | | 0.61 | | 1.55 |
|  |  | Lower CI | 0.30 | | 0.42 | | 0.50 | | 0.60 | | 0.27 | | 0.95 | | 0.27 | | 0.67 |
|  |  | Upper CI | 1.39 | | 1.89 | | 2.54 | | 3.07 | | 1.23 | | 4.18 | | 1.37 | | 3.56 |
|  |  | p value | 0.26 | | 0.77 | | 0.77 | | 0.47 | | 0.15 | | 0.07 | | 0.23 | | 0.30 |
|  | 4 vs 3 | Odds ratio | 0.39 | | 0.88 | | 1.13 | | 1.06 | | 0.90 | | 1.65 | | 0.96 | | 1.04 |
|  |  | Lower CI | 0.18 | | 0.41 | | 0.54 | | 0.46 | | 0.44 | | 0.79 | | 0.47 | | 0.47 |
|  |  | Upper CI | 0.88 | | 1.91 | | 2.34 | | 2.44 | | 1.85 | | 3.47 | | 1.95 | | 2.29 |
|  |  | p value | **0.02** | | 0.75 | | 0.75 | | 0.88 | | 0.77 | | 0.18 | | 0.90 | | 0.92 |
|  | 5 vs 3 | Odds ratio | 0.45 | | 0.41 | | 0.32 | | 0.59 | | 0.72 | | 1.74 | | 0.66 | | 0.79 |
|  |  | Lower CI | 0.19 | | 0.17 | | 0.12 | | 0.22 | | 0.33 | | 0.79 | | 0.30 | | 0.34 |
|  |  | Upper CI | 1.09 | | 1.00 | | 0.86 | | 1.57 | | 1.55 | | 3.85 | | 1.46 | | 1.84 |
|  |  | p value | 0.08 | | 0.05 | | **0.02** | | 0.29 | | 0.40 | | 0.17 | | 0.30 | | 0.58 |
| **IV** | 1 vs 3 | Odds ratio | 1.04 | | 1.48 | | - | | - | | 1.27 | | 1.54 | | - | | - |
|  |  | Lower CI | 0.47 | | 0.60 | | - | | - | | 0.56 | | 0.65 | | - | | - |
|  |  | Upper CI | 2.30 | | 3.67 | | - | | - | | 2.86 | | 3.66 | | - | | - |
|  |  | p value | 0.93 | | 0.40 | | - | | - | | 0.57 | | 0.33 | | - | | - |
|  | 2 vs 3 | Odds ratio | 0.84 | | 0.89 | | - | | - | | 0.80 | | 1.04 | | - | | - |
|  |  | Lower CI | 0.40 | | 0.39 | | - | | - | | 0.38 | | 0.47 | | - | | - |
|  |  | Upper CI | 1.79 | | 2.05 | | - | | - | | 1.70 | | 2.31 | | - | | - |
|  |  | p value | 0.66 | | 0.79 | | - | | - | | 0.56 | | 0.91 | | - | | - |
|  | 4 vs 3 | Odds ratio | 1.07 | | 2.87 | | - | | - | | 1.69 | | 0.63 | | - | | - |
|  |  | Lower CI | 0.49 | | 1.07 | | - | | - | | 0.79 | | 0.28 | | - | | - |
|  |  | Upper CI | 2.35 | | 7.72 | | - | | - | | 3.62 | | 1.40 | | - | | - |
|  |  | p value | 0.86 | | **0.04** | | - | | - | | 0.18 | | 0.26 | | - | | - |
|  | 5 vs 3 | Odds ratio | 0.52 | | 1.41 | | - | | - | | 1.01 | | 1.16 | | - | | - |
|  |  | Lower CI | 0.20 | | 0.55 | | - | | - | | 0.45 | | 0.53 | | - | | - |
|  |  | Upper CI | 1.36 | | 3.61 | | - | | - | | 2.27 | | 2.52 | | - | | - |
|  |  | p value | 0.18 | | 0.47 | | - | | - | | 0.97 | | 0.71 | | - | | - |
| **Enteral** | 1 vs 3 | Odds ratio | - | | 1.46 | | 1.71 | | 1.39 | | - | | 1.13 | | 1.15 | | 1.11 |
|  |  | Lower CI | - | | 0.61 | | 0.79 | | 0.61 | | - | | 0.48 | | 0.53 | | 0.46 |
|  |  | Upper CI | - | | 3.47 | | 3.69 | | 3.16 | | - | | 2.64 | | 2.50 | | 2.64 |
|  |  | p value | - | | 0.39 | | 0.18 | | 0.44 | | - | | 0.79 | | 0.72 | | 0.82 |
|  | 2 vs 3 | Odds ratio | - | | 1.02 | | 1.35 | | 1.15 | | - | | 2.04 | | 1.23 | | 1.15 |
|  |  | Lower CI | - | | 0.47 | | 0.64 | | 0.54 | | - | | 0.94 | | 0.58 | | 0.54 |
|  |  | Upper CI | - | | 2.22 | | 2.85 | | 2.44 | | - | | 4.43 | | 2.61 | | 2.44 |
|  |  | p value | - | | 0.95 | | 0.44 | | 0.72 | | - | | 0.07 | | 0.60 | | 0.71 |
|  | 4 vs 3 | Odds ratio | - | | 0.80 | | 0.47 | | 1.03 | | - | | 1.59 | | 0.67 | | 0.44 |
|  |  | Lower CI | - | | 0.34 | | 0.20 | | 0.45 | | - | | 0.72 | | 0.30 | | 0.19 |
|  |  | Upper CI | - | | 1.87 | | 1.13 | | 2.40 | | - | | 3.53 | | 1.50 | | 1.01 |
|  |  | p value | - | | 0.60 | | 0.09 | | 0.94 | | - | | 0.25 | | 0.33 | | 0.05 |
|  | 5 vs 3 | Odds ratio | - | | 0.83 | | 0.95 | | 0.83 | | - | | 1.33 | | 0.79 | | 0.19 |
|  |  | Lower CI | - | | 0.37 | | 0.34 | | 0.32 | | - | | 0.62 | | 0.31 | | 0.07 |
|  |  | Upper CI | - | | 1.86 | | 2.67 | | 2.16 | | - | | 2.85 | | 1.97 | | 0.48 |
|  |  | p value | - | | 0.66 | | 0.92 | | 0.70 | | - | | 0.47 | | 0.61 | | **<0.01** |
| **Target growth** | | | **Birth to 4 weeks** | | | | | | | | **Birth to 36 weeks corrected age** | | | | | | |
| **Predictor** | **Quintile** | **Value** | **Week 1** | | **Week 2** | | **Week 3** | | **Week 4** | | **Week 1** | | **Week 2** | | **Week 3** | | **Week 4** |
| **Fluid** | | |  | |  | |  | |  | |  | |  | |  | |  |
| **Total** | 1 vs 3 | Odds ratio | 2.00 | | 3.06 | | 1.20 | | 0.72 | | 2.31 | | 1.18 | | 0.37 | | 0.64 |
|  |  | Lower CI | 0.67 | | 1.05 | | 0.43 | | 0.26 | | 0.77 | | 0.41 | | 0.14 | | 0.25 |
|  |  | Upper CI | 6.00 | | 8.89 | | 3.39 | | 2.04 | | 6.93 | | 3.35 | | 0.99 | | 1.67 |
|  |  | p value | 0.22 | | **0.04** | | 0.73 | | 0.54 | | 0.13 | | 0.76 | | **0.05** | | 0.37 |
|  | 2 vs 3 | Odds ratio | 1.35 | | 1.70 | | 1.23 | | 1.20 | | 1.90 | | 1.09 | | 0.53 | | 1.04 |
|  |  | Lower CI | 0.53 | | 0.57 | | 0.47 | | 0.48 | | 0.76 | | 0.40 | | 0.22 | | 0.44 |
|  |  | Upper CI | 3.46 | | 5.07 | | 3.25 | | 3.01 | | 4.73 | | 2.98 | | 1.28 | | 2.47 |
|  |  | p value | 0.54 | | 0.35 | | 0.68 | | 0.70 | | 0.17 | | 0.87 | | 0.16 | | 0.93 |
|  | 4 vs 3 | Odds ratio | 0.75 | | 1.71 | | 1.64 | | 0.99 | | 1.56 | | 1.46 | | 0.71 | | 0.80 |
|  |  | Lower CI | 0.29 | | 0.57 | | 0.60 | | 0.37 | | 0.64 | | 0.56 | | 0.29 | | 0.30 |
|  |  | Upper CI | 1.97 | | 5.13 | | 4.43 | | 2.67 | | 3.77 | | 3.78 | | 1.78 | | 2.12 |
|  |  | p value | 0.56 | | 0.34 | | 0.33 | | 0.98 | | 0.33 | | 0.44 | | 0.47 | | 0.66 |
|  | 5 vs 3 | Odds ratio | 0.36 | | 1.27 | | 1.16 | | 0.68 | | 0.23 | | 1.53 | | 0.81 | | 1.22 |
|  |  | Lower CI | 0.09 | | 0.41 | | 0.41 | | 0.24 | | 0.05 | | 0.56 | | 0.33 | | 0.49 |
|  |  | Upper CI | 1.53 | | 3.97 | | 3.30 | | 1.95 | | 1.18 | | 4.14 | | 1.98 | | 3.08 |
|  |  | p value | 0.17 | | 0.68 | | 0.77 | | 0.47 | | 0.08 | | 0.40 | | 0.65 | | 0.67 |

| **IV** | 1 vs 3 | Odds ratio | 1.20 | 1.31 | 1.15 | - | 0.97 | 0.89 | 1.18 | - |
| --- | --- | --- | --- | --- | --- | --- | --- | --- | --- | --- |
|  |  | Lower CI | 0.44 | 0.46 | 0.47 | - | 0.37 | 0.35 | 0.53 | - |
|  |  | Upper CI | 3.23 | 3.69 | 2.84 | - | 2.53 | 2.26 | 2.61 | - |
|  |  | p value | 0.72 | 0.62 | 0.76 | - | 0.95 | 0.80 | 0.69 | - |
|  | 2 vs 3 | Odds ratio | 0.99 | 1.86 | 1.69 | 0.99 | 1.08 | 1.51 | 0.89 | 0.89 |
|  |  | Lower CI | 0.40 | 0.71 | 0.56 | 0.37 | 0.45 | 0.65 | 0.32 | 0.36 |
|  |  | Upper CI | 2.47 | 4.82 | 5.13 | 2.67 | 2.61 | 3.54 | 2.46 | 2.19 |
|  |  | p value | 0.98 | 0.20 | 0.35 | 0.99 | 0.86 | 0.34 | 0.82 | 0.80 |
|  | 4 vs 3 | Odds ratio | 0.74 | 2.57 | 0.61 | 0.76 | 0.88 | 1.10 | 0.44 | 0.58 |
|  |  | Lower CI | 0.28 | 0.93 | 0.21 | 0.24 | 0.36 | 0.42 | 0.16 | 0.20 |
|  |  | Upper CI | 1.97 | 7.06 | 1.78 | 2.38 | 2.18 | 2.87 | 1.20 | 1.68 |
|  |  | p value | 0.55 | 0.07 | 0.37 | 0.63 | 0.79 | 0.85 | 0.11 | 0.32 |
|  | 5 vs 3 | Odds ratio | 0.48 | 0.72 | 0.82 | 0.53 | 0.47 | 0.68 | 0.20 | 0.19 |
|  |  | Lower CI | 0.13 | 0.21 | 0.26 | 0.15 | 0.14 | 0.23 | 0.05 | 0.05 |
|  |  | Upper CI | 1.73 | 2.46 | 2.62 | 1.82 | 1.53 | 2.03 | 0.77 | 0.77 |
|  |  | p value | 0.26 | 0.60 | 0.74 | 0.31 | 0.21 | 0.48 | **0.02** | **0.02** |
| **Enteral** | 1 vs 3 | Odds ratio | 0.38 | 1.77 | 0.28 | 0.53 | 1.18 | 1.03 | 0.19 | 0.34 |
|  |  | Lower CI | 0.11 | 0.63 | 0.08 | 0.19 | 0.43 | 0.35 | 0.05 | 0.10 |
|  |  | Upper CI | 1.37 | 5.00 | 0.99 | 1.51 | 3.23 | 2.98 | 0.74 | 1.14 |
|  |  | p value | 0.14 | 0.28 | **0.05** | 0.23 | 0.75 | 0.96 | **0.02** | 0.08 |
|  | 2 vs 3 | Odds ratio | 2.33 | 0.91 | 0.36 | 0.66 | 1.66 | 1.61 | 0.38 | 1.05 |
|  |  | Lower CI | 0.93 | 0.32 | 0.14 | 0.25 | 0.67 | 0.62 | 0.14 | 0.42 |
|  |  | Upper CI | 5.82 | 2.58 | 0.98 | 1.77 | 4.11 | 4.22 | 1.05 | 2.62 |
|  |  | p value | 0.07 | 0.87 | **0.05** | 0.41 | 0.27 | 0.33 | 0.06 | 0.92 |
|  | 4 vs 3 | Odds ratio | 1.06 | 1.19 | 0.65 | 0.58 | 1.23 | 1.49 | 1.75 | 1.01 |
|  |  | Lower CI | 0.41 | 0.46 | 0.27 | 0.23 | 0.48 | 0.60 | 0.79 | 0.43 |
|  |  | Upper CI | 2.72 | 3.08 | 1.57 | 1.47 | 3.16 | 3.69 | 3.88 | 2.37 |
|  |  | p value | 0.91 | 0.72 | 0.33 | 0.25 | 0.66 | 0.39 | 0.17 | 0.99 |
|  | 5 vs 3 | Odds ratio | 0.81 | 1.30 | 0.55 | 0.75 | 0.99 | 1.79 | 0.63 | 1.39 |
|  |  | Lower CI | 0.31 | 0.48 | 0.21 | 0.29 | 0.39 | 0.71 | 0.25 | 0.56 |
|  |  | Upper CI | 2.16 | 3.52 | 1.46 | 1.96 | 2.53 | 4.56 | 1.59 | 3.45 |
|  |  | p value | 0.68 | 0.61 | 0.23 | 0.56 | 0.98 | 0.22 | 0.33 | 0.48 |
| **Breastmilk** | 1 vs 3 | Odds ratio | 0.41 | 2.10 | 0.72 | 1.75 | 1.22 | 1.04 | 0.65 | 0.71 |
|  |  | Lower CI | 0.11 | 0.79 | 0.24 | 0.61 | 0.44 | 0.39 | 0.21 | 0.25 |
|  |  | Upper CI | 1.44 | 5.62 | 2.12 | 4.99 | 3.34 | 2.82 | 2.04 | 2.07 |
|  |  | p value | 0.16 | 0.14 | 0.55 | 0.30 | 0.70 | 0.94 | 0.46 | 0.54 |
|  | 2 vs 3 | Odds ratio | 2.13 | 0.51 | 0.54 | 1.39 | 1.59 | 1.14 | 0.68 | 0.96 |
|  |  | Lower CI | 0.86 | 0.16 | 0.20 | 0.48 | 0.65 | 0.45 | 0.23 | 0.36 |
|  |  | Upper CI | 5.28 | 1.56 | 1.47 | 4.03 | 3.93 | 2.93 | 1.96 | 2.57 |
|  |  | p value | 0.10 | 0.24 | 0.23 | 0.55 | 0.31 | 0.78 | 0.47 | 0.93 |
|  | 4 vs 3 | Odds ratio | 1.06 | 0.89 | 0.88 | 1.81 | 1.22 | 0.95 | 2.72 | 1.69 |
|  |  | Lower CI | 0.41 | 0.34 | 0.35 | 0.71 | 0.47 | 0.38 | 1.15 | 0.73 |
|  |  | Upper CI | 2.72 | 2.32 | 2.16 | 4.65 | 3.12 | 2.33 | 6.44 | 3.93 |
|  |  | p value | 0.91 | 0.81 | 0.77 | 0.22 | 0.69 | 0.90 | **0.02** | 0.22 |
|  | 5 vs 3 | Odds ratio | 0.80 | 1.19 | 0.59 | 1.69 | 0.98 | 1.32 | 1.34 | 1.44 |
|  |  | Lower CI | 0.30 | 0.46 | 0.21 | 0.58 | 0.38 | 0.54 | 0.52 | 0.56 |
|  |  | Upper CI | 2.12 | 3.12 | 1.61 | 4.95 | 2.51 | 3.24 | 3.46 | 3.69 |
|  |  | p value | 0.65 | 0.72 | 0.30 | 0.34 | 0.97 | 0.55 | 0.55 | 0.45 |
| **Target growth** | | |  | | | |  | | | |
| **Energy** | | |  |  |  |  |  |  |  |  |
| **Total** | 1 vs 3 | Odds ratio | 0.44 | 1.42 | 0.08 | 0.60 | 0.68 | 0.50 | 0.16 | 0.43 |
|  |  | Lower CI | 0.14 | 0.50 | 0.01 | 0.19 | 0.23 | 0.17 | 0.03 | 0.15 |
|  |  | Upper CI | 1.42 | 4.03 | 0.68 | 1.89 | 2.01 | 1.48 | 0.73 | 1.23 |
|  |  | p value | 0.17 | 0.51 | **0.02** | 0.38 | 0.49 | 0.21 | **0.02** | 0.12 |
|  | 2 vs 3 | Odds ratio | 0.50 | 1.04 | 0.74 | 1.98 | 0.73 | 1.20 | 0.66 | 0.84 |
|  |  | Lower CI | 0.20 | 0.37 | 0.29 | 0.71 | 0.30 | 0.48 | 0.26 | 0.32 |
|  |  | Upper CI | 1.27 | 2.88 | 1.86 | 5.53 | 1.76 | 2.98 | 1.65 | 2.20 |
|  |  | p value | 0.14 | 0.95 | 0.52 | 0.19 | 0.48 | 0.70 | 0.37 | 0.72 |
|  | 4 vs 3 | Odds ratio | 0.41 | 1.10 | 0.62 | 1.43 | 0.54 | 0.69 | 1.12 | 1.43 |
|  |  | Lower CI | 0.16 | 0.39 | 0.25 | 0.56 | 0.23 | 0.26 | 0.47 | 0.62 |
|  |  | Upper CI | 1.07 | 3.08 | 1.56 | 3.66 | 1.27 | 1.84 | 2.62 | 3.33 |
|  |  | p value | 0.07 | 0.85 | 0.31 | 0.45 | 0.16 | 0.46 | 0.80 | 0.41 |
|  | 5 vs 3 | Odds ratio | 0.49 | 1.85 | 1.32 | 1.37 | 0.29 | 2.07 | 1.81 | 0.98 |
|  |  | Lower CI | 0.18 | 0.68 | 0.52 | 0.49 | 0.10 | 0.80 | 0.76 | 0.38 |
|  |  | Upper CI | 1.35 | 5.02 | 3.33 | 3.80 | 0.82 | 5.35 | 4.34 | 2.50 |
|  |  | p value | 0.17 | 0.23 | 0.56 | 0.55 | **0.02** | 0.13 | 0.18 | 0.96 |

| **IV** | 1 vs 3 | Odds ratio | 0.63 | 1.35 | 1.67 | - | 1.31 | 1.25 | 1.56 | - |
| --- | --- | --- | --- | --- | --- | --- | --- | --- | --- | --- |
|  |  | Lower CI | 0.20 | 0.47 | 0.64 | - | 0.49 | 0.48 | 0.69 | - |
|  |  | Upper CI | 1.99 | 3.87 | 4.33 | - | 3.54 | 3.24 | 3.51 | - |
|  |  | p value | 0.43 | 0.58 | 0.30 | - | 0.59 | 0.65 | 0.28 | - |
|  | 2 vs 3 | Odds ratio | 0.56 | 2.61 | 1.96 | 0.90 | 0.40 | 1.92 | 0.61 | 0.67 |
|  |  | Lower CI | 0.22 | 1.00 | 0.50 | 0.29 | 0.15 | 0.80 | 0.14 | 0.23 |
|  |  | Upper CI | 1.47 | 6.83 | 7.73 | 2.80 | 1.09 | 4.60 | 2.61 | 1.98 |
|  |  | p value | 0.24 | 0.05 | 0.33 | 0.86 | 0.07 | 0.14 | 0.50 | 0.47 |
|  | 4 vs 3 | Odds ratio | 0.97 | 1.72 | 0.98 | 0.35 | 0.71 | 1.41 | 0.86 | 0.53 |
|  |  | Lower CI | 0.37 | 0.60 | 0.33 | 0.09 | 0.27 | 0.53 | 0.33 | 0.16 |
|  |  | Upper CI | 2.50 | 4.95 | 2.94 | 1.43 | 1.91 | 3.73 | 2.22 | 1.73 |
|  |  | p value | 0.94 | 0.31 | 0.98 | 0.14 | 0.50 | 0.49 | 0.75 | 0.29 |
|  | 5 vs 3 | Odds ratio | 0.93 | 2.18 | 1.29 | 0.77 | 0.75 | 1.24 | 0.25 | 0.11 |
|  |  | Lower CI | 0.31 | 0.74 | 0.40 | 0.21 | 0.26 | 0.44 | 0.06 | 0.02 |
|  |  | Upper CI | 2.82 | 6.44 | 4.21 | 2.78 | 2.13 | 3.49 | 0.97 | 0.59 |
|  |  | p value | 0.90 | 0.16 | 0.67 | 0.69 | 0.58 | 0.68 | **0.05** | **0.01** |
| **Enteral** | 1 vs 3 | Odds ratio | 0.38 | 1.54 | 0.52 | 0.91 | 1.06 | 1.23 | 0.25 | 0.38 |
|  |  | Lower CI | 0.11 | 0.56 | 0.14 | 0.31 | 0.39 | 0.42 | 0.07 | 0.13 |
|  |  | Upper CI | 1.36 | 4.24 | 1.95 | 2.62 | 2.84 | 3.66 | 0.99 | 1.18 |
|  |  | p value | 0.14 | 0.40 | 0.34 | 0.86 | 0.91 | 0.70 | **0.05** | 0.09 |
|  | 2 vs 3 | Odds ratio | 2.32 | 0.61 | 0.99 | 0.76 | 1.50 | 1.95 | 0.69 | 0.62 |
|  |  | Lower CI | 0.93 | 0.22 | 0.36 | 0.25 | 0.62 | 0.73 | 0.26 | 0.24 |
|  |  | Upper CI | 5.79 | 1.73 | 2.73 | 2.29 | 3.64 | 5.21 | 1.84 | 1.65 |
|  |  | p value | 0.07 | 0.35 | 0.98 | 0.62 | 0.37 | 0.18 | 0.45 | 0.34 |
|  | 4 vs 3 | Odds ratio | 1.06 | 0.88 | 1.35 | 1.49 | 1.01 | 2.03 | 1.57 | 1.49 |
|  |  | Lower CI | 0.41 | 0.34 | 0.53 | 0.60 | 0.40 | 0.81 | 0.67 | 0.65 |
|  |  | Upper CI | 2.73 | 2.26 | 3.43 | 3.72 | 2.58 | 5.05 | 3.68 | 3.40 |
|  |  | p value | 0.90 | 0.79 | 0.53 | 0.39 | 0.98 | 0.13 | 0.30 | 0.34 |
|  | 5 vs 3 | Odds ratio | 0.80 | 1.01 | 1.63 | 1.30 | 0.88 | 2.03 | 1.77 | 0.91 |
|  |  | Lower CI | 0.30 | 0.38 | 0.60 | 0.48 | 0.35 | 0.76 | 0.71 | 0.35 |
|  |  | Upper CI | 2.12 | 2.72 | 4.47 | 3.56 | 2.21 | 5.43 | 4.39 | 2.34 |
|  |  | p value | 0.65 | 0.98 | 0.34 | 0.61 | 0.78 | 0.16 | 0.22 | 0.84 |
| **Target growth** | | |  | | | |  | | | |
| **Protein** | | |  |  |  |  |  |  |  |  |
| **Total** | 1 vs 3 | Odds ratio | 0.63 | 0.72 | 0.54 | 0.16 | 0.76 | 0.52 | 1.65 | 0.53 |
|  |  | Lower CI | 0.17 | 0.24 | 0.14 | 0.04 | 0.26 | 0.17 | 0.54 | 0.17 |
|  |  | Upper CI | 2.35 | 2.15 | 2.13 | 0.71 | 2.16 | 1.59 | 5.10 | 1.68 |
|  |  | p value | 0.50 | 0.55 | 0.38 | **0.02** | 0.60 | 0.25 | 0.38 | 0.28 |
|  | 2 vs 3 | Odds ratio | 0.80 | 0.18 | 1.25 | 0.43 | 1.16 | 0.41 | 1.48 | 0.65 |
|  |  | Lower CI | 0.29 | 0.05 | 0.40 | 0.16 | 0.46 | 0.14 | 0.49 | 0.25 |
|  |  | Upper CI | 2.22 | 0.68 | 3.87 | 1.19 | 2.90 | 1.18 | 4.43 | 1.69 |
|  |  | p value | 0.67 | **0.01** | 0.70 | 0.10 | 0.76 | 0.10 | 0.49 | 0.37 |
|  | 4 vs 3 | Odds ratio | 0.99 | 0.44 | 3.12 | 0.76 | 0.83 | 1.30 | 3.26 | 0.90 |
|  |  | Lower CI | 0.38 | 0.18 | 1.14 | 0.31 | 0.33 | 0.54 | 1.18 | 0.37 |
|  |  | Upper CI | 2.58 | 1.08 | 8.51 | 1.88 | 2.06 | 3.18 | 8.99 | 2.19 |
|  |  | p value | 0.98 | 0.07 | **0.03** | 0.56 | 0.68 | 0.56 | **0.02** | 0.82 |
|  | 5 vs 3 | Odds ratio | 1.93 | 0.86 | 3.30 | 0.75 | 1.15 | 2.14 | 4.00 | 1.22 |
|  |  | Lower CI | 0.74 | 0.32 | 1.16 | 0.30 | 0.45 | 0.77 | 1.41 | 0.48 |
|  |  | Upper CI | 5.00 | 2.33 | 9.41 | 1.90 | 2.90 | 5.94 | 11.30 | 3.07 |
|  |  | p value | 0.18 | 0.77 | **0.03** | 0.55 | 0.77 | 0.14 | **0.01** | 0.68 |
| **IV** | 1 vs 3 | Odds ratio | 0.70 | 0.90 | - | - | 1.50 | 0.95 | - | - |
|  |  | Lower CI | 0.22 | 0.34 | - | - | 0.57 | 0.38 | - | - |
|  |  | Upper CI | 2.22 | 2.38 | - | - | 3.91 | 2.40 | - | - |
|  |  | p value | 0.55 | 0.84 | - | - | 0.41 | 0.92 | - | - |
|  | 2 vs 3 | Odds ratio | 0.83 | 0.63 | - | - | 0.79 | 1.22 | - | - |
|  |  | Lower CI | 0.30 | 0.24 | - | - | 0.30 | 0.51 | - | - |
|  |  | Upper CI | 2.30 | 1.69 | - | - | 2.12 | 2.95 | - | - |
|  |  | p value | 0.73 | 0.36 | - | - | 0.64 | 0.66 | - | - |
|  | 4 vs 3 | Odds ratio | 1.04 | 1.01 | - | - | 1.27 | 0.80 | - | - |
|  |  | Lower CI | 0.41 | 0.37 | - | - | 0.52 | 0.31 | - | - |
|  |  | Upper CI | 2.65 | 2.74 | - | - | 3.12 | 2.11 | - | - |
|  |  | p value | 0.93 | 0.98 | - | - | 0.60 | 0.66 | - | - |
|  | 5 vs 3 | Odds ratio | 1.32 | 0.68 | - | - | 1.31 | 0.83 | - | - |
|  |  | Lower CI | 0.51 | 0.26 | - | - | 0.51 | 0.32 | - | - |
|  |  | Upper CI | 3.43 | 1.81 | - | - | 3.34 | 2.15 | - | - |
|  |  | p value | 0.56 | 0.44 | - | - | 0.58 | 0.71 | - | - |

| **Enteral** | 1 vs 3 | Odds ratio | 0.38 | 1.85 | 0.72 | 0.74 | 1.17 | 1.05 | 0.21 | 0.22 |
| --- | --- | --- | --- | --- | --- | --- | --- | --- | --- | --- |
|  |  | Lower CI | 0.11 | 0.65 | 0.21 | 0.25 | 0.43 | 0.36 | 0.06 | 0.06 |
|  |  | Upper CI | 1.37 | 5.28 | 2.42 | 2.20 | 3.20 | 3.00 | 0.82 | 0.85 |
|  |  | p value | 0.14 | 0.25 | 0.59 | 0.59 | 0.76 | 0.94 | **0.02** | **0.03** |
|  | 2 vs 3 | Odds ratio | 2.33 | 1.21 | 0.75 | 0.52 | 1.67 | 1.72 | 0.50 | 0.76 |
|  |  | Lower CI | 0.93 | 0.43 | 0.26 | 0.16 | 0.68 | 0.66 | 0.18 | 0.28 |
|  |  | Upper CI | 5.82 | 3.39 | 2.18 | 1.71 | 4.13 | 4.48 | 1.36 | 2.06 |
|  |  | p value | 0.07 | 0.72 | 0.60 | 0.28 | 0.26 | 0.27 | 0.17 | 0.59 |
|  | 4 vs 3 | Odds ratio | 1.06 | 1.13 | 1.97 | 1.57 | 1.13 | 1.69 | 1.51 | 1.41 |
|  |  | Lower CI | 0.41 | 0.42 | 0.79 | 0.63 | 0.44 | 0.68 | 0.65 | 0.61 |
|  |  | Upper CI | 2.73 | 3.06 | 4.90 | 3.90 | 2.91 | 4.21 | 3.53 | 3.25 |
|  |  | p value | 0.90 | 0.80 | 0.15 | 0.33 | 0.81 | 0.26 | 0.34 | 0.42 |
|  | 5 vs 3 | Odds ratio | 0.81 | 1.87 | 1.37 | 1.29 | 1.08 | 2.07 | 1.83 | 1.29 |
|  |  | Lower CI | 0.30 | 0.67 | 0.51 | 0.48 | 0.42 | 0.78 | 0.73 | 0.51 |
|  |  | Upper CI | 2.17 | 5.24 | 3.67 |  | 2.74 | 5.49 | 4.56 | 3.24 |
|  |  | p value | 0.68 | 0.23 | 0.53 |  | 0.87 | 0.14 | 0.20 | 0.59 |
| **Target growth** | | |  | | | |  | | | |
| **Fat** | | |  |  |  |  |  |  |  |  |
| **IV** | 1 vs 3 | Odds ratio | 0.74 | 1.07 | - | - | 2.01 | 0.96 | - | - |
|  |  | Lower CI | 0.25 | 0.34 | - | - | 0.69 | 0.36 | - | - |
|  |  | Upper CI | 2.20 | 3.30 | - | - | 5.87 | 2.53 | - | - |
|  |  | p value | 0.59 | 0.91 | - | - | 0.20 | 0.93 | - | - |
|  | 2 vs 3 | Odds ratio | 0.52 | 0.97 | - | - | 2.44 | 1.68 | - | - |
|  |  | Lower CI | 0.16 | 0.32 | - | - | 0.87 | 0.70 | - | - |
|  |  | Upper CI | 1.71 | 2.91 | - | - | 6.86 | 4.00 | - | - |
|  |  | p value | 0.28 | 0.95 | - | - | 0.09 | 0.24 | - | - |
|  | 4 vs 3 | Odds ratio | 1.57 | 1.78 | - | - | 1.15 | 1.40 | - | - |
|  |  | Lower CI | 0.63 | 0.65 | - | - | 0.45 | 0.53 | - | - |
|  |  | Upper CI | 3.90 | 4.86 | - | - | 2.96 | 3.71 | - | - |
|  |  | p value | 0.33 | 0.26 | - | - | 0.77 | 0.50 | - | - |
|  | 5 vs 3 | Odds ratio | 1.29 | 1.52 | - | - | 0.76 | 1.34 | - | - |
|  |  | Lower CI | 0.48 | 0.41 | - | - | 0.23 | 0.45 | - | - |
|  |  | Upper CI | 3.46 | 5.62 | - | - | 2.50 | 4.04 | - | - |
|  |  | p value | 0.61 | 0.53 | - | - | 0.66 | 0.60 | - | - |
| **Enteral** | 1 vs 3 | Odds ratio | - | 0.99 | 1.58 | 0.27 | - | 0.98 | 0.16 | 0.34 |
|  |  | Lower CI | - | 0.35 | 0.57 | 0.07 | - | 0.34 | 0.04 | 0.10 |
|  |  | Upper CI | - | 2.83 | 4.39 | 0.97 | - | 2.82 | 0.63 | 1.16 |
|  |  | p value | - | 0.99 | 0.38 | **0.04** | - | 0.96 | **0.01** | 0.08 |
|  | 2 vs 3 | Odds ratio | - | 2.30 | 0.51 | 0.37 | - | 1.33 | 0.25 | 1.07 |
|  |  | Lower CI | - | 0.90 | 0.17 | 0.14 | - | 0.51 | 0.09 | 0.43 |
|  |  | Upper CI | - | 5.88 | 1.48 | 0.99 | - | 3.45 | 0.74 | 2.69 |
|  |  | p value | - | 0.08 | 0.21 | **0.05** | - | 0.56 | **0.01** | 0.88 |
|  | 4 vs 3 | Odds ratio | - | 1.30 | 1.00 | 0.60 | - | 1.50 | 1.35 | 0.94 |
|  |  | Lower CI | - | 0.47 | 0.40 | 0.24 | - | 0.63 | 0.62 | 0.39 |
|  |  | Upper CI | - | 3.62 | 2.50 | 1.46 | - | 3.61 | 2.94 | 2.24 |
|  |  | p value | - | 0.61 | 0.99 | 0.26 | - | 0.36 | 0.46 | 0.88 |
|  | 5 vs 3 | Odds ratio | - | 2.34 | 1.02 | 0.53 | - | 1.51 | 0.54 | 1.51 |
|  |  | Lower CI | - | 0.67 | 0.39 | 0.20 | - | 0.60 | 0.22 | 0.62 |
|  |  | Upper CI | - | 8.19 | 2.69 | 1.43 | - | 3.84 | 1.38 | 3.71 |
|  |  | p value | - | 0.18 | 0.97 | 0.21 | - | 0.39 | 0.20 | 0.36 |
| **Target growth** | | |  | | | |  | | | |
| **Carbohydrate** | | |  |  |  |  |  |  |  |  |
| **IV** | 1 vs 3 | Odds ratio | 0.73 | 1.02 | - | - | 0.84 | 0.66 | - | - |
|  |  | Lower CI | 0.27 | 0.38 | - | - | 0.33 | 0.26 | - | - |
|  |  | Upper CI | 2.01 | 2.77 | - | - | 2.19 | 1.67 | - | - |
|  |  | p value | 0.54 | 0.97 | - | - | 0.73 | 0.38 | - | - |
|  | 2 vs 3 | Odds ratio | 0.91 | 1.22 | - | - | 0.96 | 1.15 | - | - |
|  |  | Lower CI | 0.37 | 0.48 | - | - | 0.39 | 0.50 | - | - |
|  |  | Upper CI | 2.25 | 3.09 | - | - | 2.35 | 2.67 | - | - |
|  |  | p value | 0.83 | 0.68 | - | - | 0.92 | 0.74 | - | - |
|  | 4 vs 3 | Odds ratio | 1.36 | 0.86 | - | - | 0.49 | 0.71 | - | - |
|  |  | Lower CI | 0.49 | 0.31 | - | - | 0.17 | 0.27 | - | - |
|  |  | Upper CI | 3.77 | 2.40 | - | - | 1.42 | 1.85 | - | - |
|  |  | p value | 0.56 | 0.78 | - | - | 0.19 | 0.49 | - | - |
|  | 5 vs 3 | Odds ratio | 0.82 | 1.49 | - | - | 0.93 | 0.67 | - | - |
|  |  | Lower CI | 0.24 | 0.52 | - | - | 0.31 | 0.24 | - | - |
|  |  | Upper CI | 2.82 | 4.26 | - | - | 2.73 | 1.89 | - | - |
|  |  | p value | 0.76 | 0.46 | - | - | 0.89 | 0.45 | - | - |

| **Enteral** | 1 vs 3 | Odds ratio | - | 1.72 | 0.54 | 0.74 | - | 1.04 | 0.23 | 0.24 |
| --- | --- | --- | --- | --- | --- | --- | --- | --- | --- | --- |
|  |  | Lower CI | - | 0.61 | 0.15 | 0.26 | - | 0.36 | 0.06 | 0.07 |
|  |  | Upper CI | - | 4.83 | 1.97 | 2.08 | - | 2.99 | 0.88 | 0.80 |
|  |  | p value | - | 0.31 | 0.35 | 0.56 | - | 0.95 | **0.03** | **0.02** |
|  | 2 vs 3 | Odds ratio | - | 0.89 | 1.06 | 0.40 | - | 1.62 | 0.62 | 0.52 |
|  |  | Lower CI | - | 0.31 | 0.40 | 0.11 | - | 0.61 | 0.24 | 0.19 |
|  |  | Upper CI | - | 2.56 | 2.86 | 1.37 | - | 4.27 | 1.62 | 1.42 |
|  |  | p value | - | 0.83 | 0.90 | 0.14 | - | 0.33 | 0.33 | 0.20 |
|  | 4 vs 3 | Odds ratio | - | 1.29 | 1.42 | 1.12 | - | 1.85 | 1.32 | 1.06 |
|  |  | Lower CI | - | 0.50 | 0.55 | 0.46 | - | 0.76 | 0.54 | 0.47 |
|  |  | Upper CI | - | 3.32 | 3.66 | 2.75 | - | 4.50 | 3.23 | 2.41 |
|  |  | p value | - | 0.60 | 0.46 | 0.80 | - | 0.18 | 0.54 | 0.89 |
|  | 5 vs 3 | Odds ratio | - | 1.57 | 1.61 | 1.24 | - | 2.01 | 1.82 | 0.84 |
|  |  | Lower CI | - | 0.57 | 0.60 | 0.46 | - | 0.75 | 0.73 | 0.34 |
|  |  | Upper CI | - | 4.33 | 4.34 | 3.30 | - | 5.33 | 4.54 | 2.11 |
|  |  | p value | - | 0.38 | 0.35 | 0.67 | - | 0.16 | 0.20 | 0.71 |
| **Target growth** | | |  | | | |  | | | |
| **Energy:protein ratio** | | |  |  |  |  |  |  |  |  |
| **Total** | 1 vs 3 | Odds ratio | 0.86 | 0.73 | 0.88 | 0.55 | 1.42 | 0.83 | 0.43 | 0.70 |
|  |  | Lower CI | 0.34 | 0.28 | 0.34 | 0.21 | 0.57 | 0.31 | 0.16 | 0.26 |
|  |  | Upper CI | 2.20 | 1.92 | 2.28 | 1.45 | 3.54 | 2.25 | 1.17 | 1.94 |
|  |  | p value | 0.75 | 0.52 | 0.79 | 0.23 | 0.46 | 0.71 | 0.10 | 0.50 |
|  | 2 vs 3 | Odds ratio | 0.56 | 1.23 | 0.41 | 0.81 | 0.64 | 1.98 | 0.59 | 1.28 |
|  |  | Lower CI | 0.21 | 0.48 | 0.14 | 0.31 | 0.25 | 0.81 | 0.22 | 0.48 |
|  |  | Upper CI | 1.44 | 3.14 | 1.17 | 2.09 | 1.68 | 4.85 | 1.58 | 3.38 |
|  |  | p value | 0.23 | 0.66 | 0.10 | 0.66 | 0.36 | 0.13 | 0.30 | 0.62 |
|  | 4 vs 3 | Odds ratio | 0.32 | 0.79 | 1.09 | 0.47 | 0.87 | 1.21 | 0.90 | 0.98 |
|  |  | Lower CI | 0.11 | 0.28 | 0.45 | 0.17 | 0.36 | 0.47 | 0.39 | 0.38 |
|  |  | Upper CI | 0.91 | 2.24 | 2.64 | 1.34 | 2.09 | 3.11 | 2.07 | 2.51 |
|  |  | p value | **0.03** | 0.66 | 0.85 | 0.16 | 0.75 | 0.70 | 0.80 | 0.96 |
|  | 5 vs 3 | Odds ratio | 0.51 | 0.68 | 0.20 | 0.19 | 0.52 | 0.97 | 0.60 | 0.91 |
|  |  | Lower CI | 0.16 | 0.22 | 0.05 | 0.05 | 0.18 | 0.33 | 0.23 | 0.32 |
|  |  | Upper CI | 1.62 | 2.13 | 0.75 | 0.75 | 1.53 | 2.82 | 1.57 | 2.60 |
|  |  | p value | 0.25 | 0.50 | **0.02** | **0.02** | 0.23 | 0.96 | 0.30 | 0.86 |
| **IV** | 1 vs 3 | Odds ratio | 0.93 | 2.71 | - | - | 2.01 | 1.50 | - | - |
|  |  | Lower CI | 0.36 | 0.78 | - | - | 0.79 | 0.51 | - | - |
|  |  | Upper CI | 2.39 | 9.43 | - | - | 5.13 | 4.38 | - | - |
|  |  | p value | 0.88 | 0.12 | - | - | 0.14 | 0.46 | - | - |
|  | 2 vs 3 | Odds ratio | 0.69 | 2.35 | - | - | 0.88 | 1.19 | - | - |
|  |  | Lower CI | 0.28 | 0.74 | - | - | 0.33 | 0.45 | - | - |
|  |  | Upper CI | 1.75 | 7.43 | - | - | 2.29 | 3.15 | - | - |
|  |  | p value | 0.44 | 0.15 | - | - | 0.79 | 0.73 | - | - |
|  | 4 vs 3 | Odds ratio | 0.56 | 7.24 | - | - | 1.41 | 0.41 | - | - |
|  |  | Lower CI | 0.21 | 1.77 | - | - | 0.57 | 0.14 | - | - |
|  |  | Upper CI | 1.51 | 29.63 | - | - | 3.49 | 1.22 | - | - |
|  |  | p value | 0.25 | **0.01** | - | - | 0.46 | 0.11 | - | - |
|  | 5 vs 3 | Odds ratio | 0.49 | 3.03 | - | - | 0.68 | 0.68 | - | - |
|  |  | Lower CI | 0.14 | 0.80 | - | - | 0.22 | 0.24 | - | - |
|  |  | Upper CI | 1.73 | 11.56 | - | - | 2.12 | 1.91 | - | - |
|  |  | p value | 0.27 | 0.10 | - | - | 0.51 | 0.46 | - | - |
| **Enteral** | 1 vs 3 | Odds ratio | - | 1.39 | 0.81 | 1.29 | - | 1.36 | 1.53 | 0.61 |
|  |  | Lower CI | - | 0.46 | 0.32 | 0.47 | - | 0.48 | 0.60 | 0.22 |
|  |  | Upper CI | - | 4.20 | 2.05 | 3.55 | - | 3.89 | 3.93 | 1.70 |
|  |  | p value | - | 0.56 | 0.66 | 0.63 | - | 0.56 | 0.37 | 0.35 |
|  | 2 vs 3 | Odds ratio | - | 1.28 | 0.94 | 1.67 | - | 1.58 | 1.88 | 1.31 |
|  |  | Lower CI | - | 0.49 | 0.38 | 0.67 | - | 0.64 | 0.77 | 0.58 |
|  |  | Upper CI | - | 3.38 | 2.33 | 4.13 | - | 3.93 | 4.58 | 2.96 |
|  |  | p value | - | 0.61 | 0.89 | 0.27 | - | 0.32 | 0.17 | 0.52 |
|  | 4 vs 3 | Odds ratio | - | 0.97 | 0.35 | 0.68 | - | 0.60 | 0.56 | 0.34 |
|  |  | Lower CI | - | 0.31 | 0.11 | 0.22 | - | 0.22 | 0.20 | 0.12 |
|  |  | Upper CI | - | 3.03 | 1.12 | 2.10 | - | 1.65 | 1.63 | 1.01 |
|  |  | p value | - | 0.96 | 0.08 | 0.50 | - | 0.32 | 0.29 | 0.05 |
|  | 5 vs 3 | Odds ratio | - | 1.30 | 0.71 | 0.67 | - | 0.73 | 0.44 | 0.17 |
|  |  | Lower CI | - | 0.47 | 0.19 | 0.19 | - | 0.28 | 0.13 | 0.05 |
|  |  | Upper CI | - | 3.61 | 2.69 | 2.37 | - | 1.90 | 1.54 | 0.63 |
|  |  | p value | - | 0.61 | 0.62 | 0.54 | - | 0.52 | 0.20 | **0.01** |

Data are adjusted OR and 95% confidence interval (CI) for achieving expected or target growth for each quintile of intake in each of the first 4 weeks, where quintile 3 is the referent. Significant p values are shown in bold. A dash indicates insufficient data for analysis in the week. IV, intravenous.

**Supplementary table 2B** Quintiles of nutrient intakes in each of the first four weeks

| Predictor | Route | Quintile | Week 1 | Week 2 | Week 3 | Week 4 |
| --- | --- | --- | --- | --- | --- | --- |
| Fluid (mL.Kg^-1^.d^-1^) | Total | 1 | 64-122 | 120-151 | 103-159 | 98-156 |
|  |  | 2 | 123-133 | 152-161 | 157-165 | 157-165 |
|  |  | 3 | 134-143 | 162-171 | 166-175 | 166-173 |
|  |  | 4 | 144-155 | 172-178 | 176-181 | 174-180 |
|  |  | 5 | 156-213 | 179-215 | 182-212 | 181-220 |
|  | Intravenous | 1 | 45-100 | 0-26 | 0 | 0 |
|  |  | 2 | 101-118 | 27-62 | 0.1-5 | 0.1-1 |
|  |  | 3 | 119-130 | 63-102 | 6-32 | 1-14 |
|  |  | 4 | 131-144 | 103-138 | 33-115 | 15-88 |
|  |  | 5 | 145-210 | 139-210 | 116-212 | 89-177 |
|  | Enteral | 1 | 0-5 | 0-21 | 0-46 | 0-70 |
|  |  | 2 | 6-8 | 22-63 | 47-134 | 71-150 |
|  |  | 3 | 9-15 | 64-103 | 135-160 | 151-165 |
|  |  | 4 | 16-26 | 104-137 | 161-176 | 166-178 |
|  |  | 5 | 27-77 | 138-186 | 177-202 | 179-210 |
|  | Breastmilk | 1 | 0-4 | 0-19 | 0-31 | 0-42 |
|  |  | 2 | 5-8 | 20-59 | 32-114 | 43-125 |
|  |  | 3 | 9-15 | 60-100 | 115-156 | 126-161 |
|  |  | 4 | 16-26 | 101-133 | 157-173 | 162-175 |
|  |  | 5 | 27-77 | 134-186 | 174-202 | 176-210 |
| Energy (Kcal.Kg^-1^.d^-1^) | Total | 1 | 35-68 | 56-91 | 53-100 | 45-106 |
|  |  | 2 | 69-74 | 92-103 | 101-121 | 107-127 |
|  |  | 3 | 75-79 | 104-114 | 122-135 | 128-142 |
|  |  | 4 | 80-85 | 115-128 | 136-151 | 143-154 |
|  |  | 5 | 86-108 | 129-156 | 152-183 | 155-177 |
|  | Intravenous | 1 | 32-56 | 0-15 | 0 | 0 |
|  |  | 2 | 57-62 | 16-35 | 0.1-1.9 | 0.1-1 |
|  |  | 3 | 63-69 | 36-58 | 2-13 | 1-4 |
|  |  | 4 | 70-75 | 59-77 | 14-64 | 5-50 |
|  |  | 5 | 76-88 | 78-104 | 65-100 | 51-100 |
|  | Enteral | 1 | 0-3 | 0-14 | 0-34 | 0-54 |
|  |  | 2 | 4-6 | 15-43 | 35-106 | 55-121 |
|  |  | 3 | 7-10 | 44-78 | 107-132 | 122-141 |
|  |  | 4 | 11-17 | 79-109 | 133-149 | 142-154 |
|  |  | 5 | 18-62 | 110-153 | 150-183 | 155-176 |
| Protein (g.Kg^-1^.d^-1^) | Total | 1 | 0.9-2.7 | 1.4-3.0 | 0.9-2.6 | 0.7-2.5 |
|  |  | 2 | 2.8-3.1 | 3.1-3.3 | 2.7-3.3 | 2.6-3.3 |
|  |  | 3 | 3.2-3.5 | 3.4-3.8 | 3.4-3.8 | 3.4-3.9 |
|  |  | 4 | 3.6-3.9 | 3.9-4.3 | 3.9-4.1 | 4.0-4.2 |
|  |  | 5 | 4-4.9 | 4.4-5.4 | 4.2-5.3 | 4.3-5.8 |
|  | Intravenous | 1 | 0.7-2.4 | 0-0.6 | 0 | 0 |
|  |  | 2 | 2.5-2.8 | 0.7-1.4 | 0.1 | 0.1 |
|  |  | 3 | 2.9-3.2 | 1.5-2.2 | 0.2-0.4 | 0.1 |
|  |  | 4 | 3.3-3.6 | 2.3-2.8 | 0.5-2.3 | 0.2-1.8 |
|  |  | 5 | 3.7-4.8 | 2.9-4 | 2.4-4 | 1.9-3.9 |
|  | Enteral | 1 | 0-0.1 | 0-0.4 | 0-0.7 | 0-1.2 |
|  |  | 2 | 0.2-02. | 0.5-1.2 | 0.8-2.2 | 1.3-2.5 |
|  |  | 3 | 0.3-0.3 | 1.3-2.1 | 2.3-3.3 | 2.6-3.7 |
|  |  | 4 | 0.4-0.5 | 2.2-3.2 | 3.4-4.1 | 3.8-4.2 |
|  |  | 5 | 0.6-2.2 | 3.3-5.1 | 4.2-5.3 | 4.3-5.8 |

| Fat (g.Kg^-1^.d^-1^) | Intravenous | 1 | 0.7-1.7 | 0.0-0.5 | 0 | 0 |
| --- | --- | --- | --- | --- | --- | --- |
|  |  | 2 | 1.8-1.9 | 0.6-1.1 | 0.1 | 0.1 |
|  |  | 3 | 2.0-2.2 | 1.2-1.8 | 0.2-0.2 | 0.1 |
|  |  | 4 | 2.3-2.6 | 1.9-2.6 | 0.3-1.8 | 0.2-1.5 |
|  |  | 5 | 2.7-3.1 | 2.7-3.5 | 1.9-3.7 | 1.6-3.3 |
|  | Enteral | 1 | 0.0-0.1 | 0 -0.7 | 0.0-1.8 | 0-2.7 |
|  |  | 2 | 0.2-0.3 | 0.8-2.1 | 1.9-5.2 | 2.8-5.9 |
|  |  | 3 | 0.4-0.5 | 2.2-3.5 | 5.3-6.2 | 6.0-6.4 |
|  |  | 4 | 0.6-0.8 | 3.6-4.6 | 6.3-6.8 | 6.5-6.9 |
|  |  | 5 | 0.9-2.5 | 4.7-6.2 | 6.9-8.3 | 7.0-9.8 |
| Carbohydrate (g.Kg^-1^.d^-1^) | **Intravenous** | 1 | 3.2-7.7 | 0-2.0 | 0 | 0 |
|  |  | 2 | 7.8-8.9 | 2.1-5.0 | 0.1-0.3 | 0.1 |
|  |  | 3 | 9.0-10.1 | 5.1-8.3 | 0.4-2.6 | 0.2-1.1 |
|  |  | 4 | 10.2-11.4 | 8.4-11.6 | 2.7-9.2 | 1.2-7.3 |
|  |  | 5 | 11.5-14.0 | 11.7-16.4 | 9.3-17.7 | 7.4-16.6 |
|  | Enteral | 1 | 0-0.3 | 0-1.3 | 0-3.0 | 0-4.7 |
|  |  | 2 | 0.4-0.5 | 1.4-4.0 | 3.1-9.5 | 4.8-10.9 |
|  |  | 3 | 0.6-0.9 | 4.1-7.2 | 9.6-12.6 | 11-14 |
|  |  | 4 | 1.0-1.6 | 7.3-10.7 | 12.7-15.0 | 14.1-15.7 |
|  |  | 5 | 1.7-6.4 | 10.8-16.6 | 15.118.9 | 15.8-18.2 |
| Energy:protein ratio  (Kcal.g ^-1^ protein) | Total | 1 | 13-19 | 17-27 | 18-32 | 18-33 |
|  |  | 2 | 20-22 | 28-30 | 33-36 | 34-37 |
|  |  | 3 | 23-25 | 31-31 | 37-38 | 38 |
|  |  | 4 | 26-29 | 32-36 | 39-46 | 39-46 |
|  |  | 5 | 30-706 | 37-267 | 47-361 | 47-376 |
|  | Intravenous | 1 | 10-17 | 10-20 | 9-20 | 12-22 |
|  |  | 2 | 18-21 | 21-23 | 21-25 | 23-26 |
|  |  | 3 | 22-24 | 24-28 | 26-29 | 27-30 |
|  |  | 4 | 25-29 | 29-33 | 30-33 | 31-34 |
|  |  | 5 | 30-135 | 34-164 | 34-205 | 35-55 |
|  | Enteral | 1 | 31-37 | 22-32 | 28-37 | 28-35 |
|  |  | 2 | 38-37 | 33-36 | 38 | 36-38 |
|  |  | 3 | 38-37 | 37-39 | 39-49 | 39-44 |
|  |  | 4 | 38-37 | 40-39 | 50-54 | 45-54 |
|  |  | 5 | 38-53 | 40-73 | 55-57 | 55-72 |
